# Chinmedomics Strategy for Elucidating the Pharmacological Effects and Discovering Bioactive Compounds From Keluoxin Against Diabetic Retinopathy

**DOI:** 10.3389/fphar.2022.728256

**Published:** 2022-03-31

**Authors:** Ling Kong, Ye Sun, Hui Sun, Ai-hua Zhang, Bo Zhang, Nan Ge, Xi-jun Wang

**Affiliations:** National Chinmedomics Research Center, National TCM Key Laboratory of Serum Pharmacochemistry, Functional Metabolomics Laboratory, Department of Pharmaceutical Analysis, Heilongjiang University of Chinese Medicine, Harbin, China

**Keywords:** chinmedomics, pharmacological effects, bioactive compounds, herbal medicine, biomarkers

## Abstract

Keluoxin (KLX) is an active agent in the treatment of diabetic retinopathy (DR). However, its mechanism, targets, and effective constituents against DR are still unclear, which seriously restricts its clinical application. Chinmedomics has the promise of explaining the pharmacological effects of herbal medicines and investigating the effective mechanisms. The research results from electroretinography and electron microscope showed that KLX could reduce retinal dysfunction and pathological changes by the DR mouse model. Based on effectiveness, we discovered 64 blood biomarkers of DR by nontargeted metabolomics analysis, 51 of which returned to average levels after KLX treatment including leukotriene D4 and A4, l-tryptophan, 6-hydroxymelatonin, l-phenylalanine, l-tyrosine, and gamma-linolenic acid (GLA). The metabolic pathways involved were phenylalanine metabolism, steroid hormone biosynthesis, sphingolipid metabolism, etc. Adenosine monophosphate-activated protein kinase (AMPK), extracellular signal-regulated protein kinase1/2 (ERK1/2), phosphatidylinositol-3-kinase (PI3K), and protein 70 S6 kinase (p70 S6K) might be potential targets of KLX against DR. This was related to the mammalian target of rapamycin (mTOR) signaling and AMPK signaling pathways. We applied the chinmedomics strategy, integrating serum pharm-chemistry of traditional Chinese medicine (TCM) with metabolomics, to discover astragaloside IV (AS-IV), emodin, rhein, chrysophanol, and other compounds, which were the core effective constituents of KLX when against DR. Our study was the first to apply the chinmedomics strategy to discover the effective constituents of KLX in the treatment of DR, which fills the gap of unclear effective constituents of KLX. In the next step, the research of effective constituents can be used to optimize prescription preparation, improve the quality standard, and develop an innovative drug.

## Introduction

Diabetic retinopathy (DR) is an eye condition that affects blood vessels in the retina and can cause vision impairment among diabetes patients (Cheung et al., 2010). According to a survey, with a projected more than 600 million people suffering from diabetes by 2040, the prevalence of DR is expected to rapidly rise, which will lead to terrible vision impairment among those patients; therefore, it makes DR an important target for experts and doctors to prevent and treat (GBD 2019 Blindness and Vision Impairment Collaborators and Vision Loss Expert Group of the Global Burden of Disease Study, 2021). In recent years, traditional Chinese medicine (TCM) has become popular because it has a definitive curative effect and has few side effects. Keluoxin (KLX) is a Chinese patent medicine (No. Z20090035) used to treat diabetic nephropathy (DN). A meta-analysis of randomized controlled trials (RCTs) for DN that included 596 participants from 7 trials, in which 302 patients took KLX plus Western medicine and 294 took Western medicine only, showed that the synergy of KLX with Western medicine could improve levels of serum creatinine (sCr), blood urea nitrogen (BUN), α1 and β2 microglobulins, fasting blood glucose (FBG), total cholesterol, serum triglyceride (sTG), and low-density lipoprotein (LDL) and thus treat DN (Bai et al., 2019). The consensus of 23 leading experts in the fields of endocrinology and nephrology in both TCM and Western medicine is that KLX has better effects than only using Western medicine on reducing blood glucose levels, improving lipid metabolism, protecting glomerular-filtration membranes, and improving hemorheology (Specialty Committee of Endocrinology of Chinese Association of Integrative Medicine, 2020). Therefore, KLX mostly solves the problem of microvascular disease. It also has preventive and therapeutic effects on DR, one of the most common microvascular complications of diabetes (Ge et al., 2021). Clinical research shows that it can improve vision and retinal hemorheology and reduce intraocular pressure, the number of capillary hemangiomas, and vascular leakage range (Xiao 2015). Animal and cellular studies have shown that KLX can improve DR (Hu et al., 2014; Shen et al., 2014; Luo et al., 2019). Formononetin (Wu et al., 2016a), specnuezhenide (Wu et al., 2016b), and aloe-emodin (Wu et al., 2016c), the main components of KLX, can inhibit vascular endothelial growth factor (VEGF) secretion, decrease messenger ribonucleic acid (mRNA) expression of VEGF-A and prolyl-4-hydroxylase 2 (PHD-2), and reduce the protein expression of VEGF-A, hypoxia-inducible factor-1*α* (HIF-1*α*), and PHD-2.

However, the underlying mechanism and effective constituents of KLX against DR have not been elucidated. Metabolomics analysis can comprehensively demonstrate the multiple pathways and targets in which TCM plays its therapeutic role (Zhang et al., 2015; Zhang et al., 2017; Li et al., 2018). It is a powerful platform to discover biomarkers, metabolic mechanisms, and potential targets for herbal medicine (Wang et al., 2013; Sun et al., 2014; Zhang et al., 2014; Nan et al., 2016). The theory of serum pharmacochemistry of TCM provides a new means of active component analysis. Chinmedomics, which has already been widely used (Sun et al., 2019; Wang T et al., 2019; Ren et al., 2020; Xiong et al., 2020; Zhao M et al., 2020), integrates metabolomics with serum pharmacochemistry of TCM. It has established the scientific language to explain TCM’s effectiveness, explores overall changes to an organism’s metabolites, and also provides an accurate diagnosis of clinical diseases, in the meantime permitting innovative drug design (Han et al., 2020). In this study, we applied a chinmedomics strategy based on ultra-performance liquid chromatography–mass spectrometry (UPLC-MS) to evaluate the effectiveness and explore effective constituents of KLX against DR.

## Materials and Methods

### Materials and Reagents

KLX was composed of Astragali radix (Dried root of *Astragalus mongholicus* Bunge), Pseudostellariae radix (Dried root tuber of *Pseudostellaria heterophylla* (Miq.) Pax), Ligustri lucidi fructus (Dried mature fruits of *Ligustrum lucidum* W.T.Aiton), Lycii fructus (Dried mature fruits of *Lycium barbarum* L.), Rhei radix et rhizome (Dried root and rhizome of *Rheum officinale* Baill.), Hirudo (Dried body of *Hirudo nipponica* Whitman). It was provided by Chengdu Kanghong Pharmaceutical Co., Ltd., Chengdu, China. UPLC-grade methanol and acetonitrile were purchased from Merck KGaA, Darmstadt, Germany. Distilled water was obtained from Watsons Water, Guangzhou, China. Formic acid was purchased from Tianjin Kemiou Chemical Reagent Co., Ltd., Tianjin, China.

### Study Design

The aims of this study were to explore the potential effective constituents of KLX by applying the chinmedomics strategy based on correlation analysis between blood components and DR biomarkers and to discover the molecular mechanism underlying KLX’s regulatory role against DR. To define the effect of KLX on DR, a model was created by inducing DR in BKS.Cg-Dock7m^+/+^Leprdb/Nju spontaneously diabetic mice. The therapeutic effects of KLX were evaluated by electroretinography (ERG), histopathology, and metabolic profile. To elucidate the relevant mechanism, our metabolomics analysis discovered the blood biomarkers and main metabolic pathways of KLX against DR. Ingenuity Pathway Analysis (IPA) found further potential targets and related signaling pathways. The details of ERG, histopathology analysis, sample handling, and instrument acquisition are described in the Supplementary Material.

### Animals

Seven-week-old male C57BL/KsJ-db/db (db/db) mice and wild-type C57BL/KsJ-db/m (db/m) mice were provided by Nanjing Biomedical Research Institute of Nanjing University (Nanjing, China). Mice were raised at an ambient temperature of 22°C–25°C on a 12-h light/dark cycle and given free access to water and food. They were allowed to adapt to the environment for 1 week before the formal experiment. Then, all animals were divided into three groups: a control group, a model group, and a KLX-treated group. The db/m mice were used in the control group and randomly assigned the db/db mice to either the model group or the KLX-treated group. KLX was orally administered to mice in the KLX-treated group at a dose of 0.780 g/kg (10 ml/kg) 1×/day from 8 weeks of age for 24 consecutive weeks. The mice in the control and model groups were given physiological saline (10 ml/kg) 1×/day from 8 weeks of age for the same time period. The experimental scheme was approved by the Animal Care and Ethics Committee of Heilongjiang University of Chinese Medicine, and all experiments were carried out according to the Declaration of Helsinki.

### Electroretinography

The change of visual electrophysiological was a quantitative index to reflect the degree of damage to the retina’s tertiary neuron and visual-conduction pathway. The flash ERG (fERG) can reflect the structural functions of the retina. Mice were prepared for fERG analysis in a darkroom overnight, after which they were anesthetized with 0.3% pentobarbital sodium (30 mg/kg) and underwent mydriasis via tropicamide eye drops. A drop of 2% lidocaine hydrochloride (13 mg/kg) was dripped onto the corneal surface for topical anesthesia. Dark-adapted (scotopic) responses were assessed firstly. Laser intensity was set to 0.01 cd*s/m^2^ for dark-adapted, 3.0 cd*s/m^2^ for dark-adapted, and 3.0 cd*s/m^2^ for oscillatory potentials (Ops). After mice were exposed to background light for 5 min to fully adapt to the light, light-adapted (photopic) responses were obtained using white flashes. Laser intensity was set to 3.0 cd*s/m^2^. Amplitudes and peak latencies of the a- and b-waves, and OS_1_, OS_2_, OS_3_, and OS of wavelet amplitude of the Ops, were recorded. At the end of fERG, chloramphenicol eye drops were administered to prevent infection.

### Histopathology Analysis

Mice were anesthetized with 0.3% pentobarbital sodium (30 mg/kg) to remove their eyeballs for experiments; at the same time, their blood was collected for metabolomics analysis. For testing the ultrastructures of the retinal capillary vessels and optic cells, the eyes were fixed in 2.5% glutaraldehyde at 4°C. A rectangle of tissue was cut 1.5 mm × 2.0 mm from the retina, washing it in phosphate-buffered saline (PBS) and setting it with osmium tetroxide. Meanwhile, gradient dehydration was performed on it with ethanol and acetone, and then it was embedded with pure epoxy resin Epon 812. Initially, a 1-μm-thin section was cut from the rectangle for optical location, and then this ultra-thin section was prepared. Retinas stained with uranyl acetate and lead citrate were observed under an H-7650 transmission electron microscope (TEM; Hitachi, Tokyo, Japan).

### Metabolomics Analysis

#### Sample Collection and Preparation

After centrifugation at 3,500 rpm for 10 min at 4°C, the upper serum was obtained; after that serum samples were stored at −80°C until UPLC-MS analysis. After they were thawed in an ice water bath, 400 μl of methanol was added to 100 μl of serums for protein precipitation, and then the mixture was vortexed for 30 s and let stand for 10 min. After centrifugation at 13,000 rpm for 10 min at 4°C, 300 μl of supernatant was removed.

#### Instrument Parameters for Metabolomics

Chromatographic separation was performed on a Water Acquity UPLC (Waters Corp., Milford, MA, USA). We used a Waters Acquity UPLC Ethylene Bridged Hybrid (BEH) C_18_ column (2.1 × 100 mm, 1.7 μm) at 40°C. Mobile phases were acetonitrile with 0.1% formic acid (A) and water with 0.1% formic acid (B). The flow rate of the mobile phase was 0.40 ml/min. The injection volume was 5 μl. The gradient program was as follows: 0–2 min, 1%–20% A; 2–4 min, 20%–50% A; 4–7 min, 50%–70% A; 7–9 min, 70%–85% A; 9–11 min, 85–100% A; 11–15.5 min, 100% A; 15.5–16 min, 100%–1% A; and 16–18 min, 1% A.

MS data were acquired by using an AB SCIEX Triple TOF 5600^+^ detector (AB SCIEX, Framingham, MA, USA). Positive- and negative-ionization modes were acquired via DuoSpray electrospray ionization (ESI). Optimal conditions for high-resolution MS analysis were as follows: IonSpray voltage floating (ISVF), 5,000 V (positive) and −4000 V (negative); declustering potential (DP), 100 V (positive) and −100 V (negative); collision energy (CE), 10 V (positive) and −10 V (negative); ion source gases 1 and 2 (GS1 and GS2), 60 psi each; curtain gas (CUR), 25 psi; source temperature (TEM), 600°C; and accumulation time, 0.1 s. By combining dynamic background subtraction (DBS) with information-dependent acquisition (IDA), MS/MS data were acquired. Any time-of-flight (TOF)-MS survey scan peak exceeding 100 cps was selected to acquire dependent scans in IDA, and 10 candidate ions were allowed per cycle. Optimal conditions for high-sensitivity IDA analysis were as follows: CE, 40 V (positive) and −40 V (negative); collision energy spread (CES), 15 V; accumulation time, 0.05 s; and other IDA parameters, same as those for MS. The mass/charge ratio (m/z) range of MS and MS/MS acquisition was 50–1,000 amu. Instrument calibration solutions were purchased from AB SCIEX. MS and MS/MS were automatically adjusted and corrected via external-standard correction method, and every 10 sample injections acquired 1 calibration sample.

### Constituent Analysis of Keluoxin *In Vivo* and *In Vitro*

#### Sample Preparation for Constituent Analysis

Into the KLX sample of Astragali radix, Pseudostellariae radix, Ligustri lucidi fructus, Lycii fructus, Rhei radix et rhizome, and Hirudo, 10 ml of 75% methanol was added, and it was shaken and mixed for 30 s, ultrasonicated for 10 min, and then centrifuged at 13,000 rpm for 10 min. The supernatant was obtained for constituent analysis *in vitro*. The mice were anesthetized with 0.3% pentobarbital sodium (30 mg/kg), and the blood was obtained by removing the eyeballs after oral administration of KLX or physiological saline for 120 min. Then, serum was obtained by centrifugation for 10 min at 3,500 rpm at 4°C after letting the sample rest for 30 min. Phosphoric acid (10 μl) was added to 200 μl of serum, and the mixture was vortexed for 30 s and ultrasonicated for 1 min. The mixed sample was added to a pre-activated Oasis hydrophilic–lipophilic balance (HLB) C18 solid-phase extraction column (1 cc/30 mg, 30 μm, Waters). The column was washed with 1 ml of water and then 1 ml of 5% methanol, after which the solution was discarded. The column continued to elute with 1 ml of methanol. The eluent was collected and dried under vacuum-centrifugal pressure at 35°C. The residue was dissolved with 200 μl of methanol. After centrifugation at 13,000 rpm for 15 min, the supernatant was separated to analyze the constituent using a UPLC-Q/TOF-MS system. The instrument parameters for constituent analysis are described in the Supplementary Methods.

#### Instrument Parameters for Constituent Analysis

To screen KLX sample ingredients *in vivo* and *in vitro*, a UPLC system (Waters) coupled with an MS system (AB SCIEX) was used. The mobile phase was 0.1% formic acid acetonitrile (A) and 0.1% formic acid water (B). The column temperature was 40°C. The gradient program was 0–2 min, 1%–15% A; 2–10 min, 15%–50% A; 10–14.5 min, 50–100% A; 14.5–16.5 min, 100% A; 16.5–17 min, 100%–1% A; and 17–20 min, 1% A. For MS analysis, 5.0 μl was taken. The workstation software for TOF-MS was Analyst TF version 1.6.2 (AB SCIEX). Optimized conditions for positive- and negative-ion modes were as follows: ISVF, 5000 and −4000 V; TEM, 600°C; GS1 and GS2, 60 psi each; CUR, 25 psi; DP, 100 and −100 V; CE (MS), 10 and −10 V; CE (IDA), 40 and −40 V; and CES, 15 V. The total accumulation time was 0.1 s for the TOF-MS survey plus 0.05 s for the IDA product ion to acquire enough MS data points. The m/z range of MS and MS/MS acquisition was 50–1,000 amu.

### Multivariate Statistical Analysis and Data Preprocessing

Raw UPLC-MS data with wiff format were preprocessed using Progenesis QI software version 2.0 (Waters Corp., Milford, MA, USA), including peak recognition, peak alignment, peak picking, and data normalization. The data format selected profile data, and ion mode selected positive- or negative-ionization polarity. Adducts were [M + H]^+^ and [M + Na]^+^ for positive ions; [M − H]^−^ and [M + FA − H]^−^ for negative ions. In the absence of metabolic data, missing metabolites were removed per the modified 80% rule; generally, the median value of samples in a group was used instead of the missing value. Preprocessed data were exported to Waters Umetrics EZinfo software version 3.0 for principal component analysis (PCA) and orthogonal partial least squares discriminant analysis (OPLS-DA). Ions with variable importance in projection (VIP) >1 and *p*-value (by *t*-test) <0.05 were considered potential biomarkers.

### Biomarker Identification and Metabolic Pathway

Candidate ions were selected based on VIP and *t*-test results. By searching the online ChemSpider database (http://www.chemspider.com/) and offline Human Metabolome Database (HMDB; https://hmdb.ca/) in the Progenesis QI software, and then combining the network MS/MS information of these databases, MassBank (https://massbank.eu/) and other MS databases, potential biomarkers were rapidly characterized. For visual analysis of metabolic-interaction pathways, the MetaboAnalyst and Kyoto Encyclopedia of Genes and Genomes (KEGG) databases were used.

### Potential Prediction of Targets in Keluoxin Effect Against Diabetic Retinopathy

IPA is a multi-platform biological-information tool for query and analysis. To analyze interactions among metabolites, enzymes, and proteins, data of blood biomarkers of KLX regulating DR into IPA further were imported. Integrating the metabolic pathway with the IPA and KEGG databases permitted us to explore the critical targets in KLX’s treatment of DR.

### Constituent Analysis and Identification

High-resolution LC/MS data were obtained from Progenesis QI, including retention time, accurate mass, peak intensity, and MS/MS spectra. Chemical structures were further identified and characterized using this software (Waters, United States). Ions with high intensity in the KLX-treated group and low intensity in the model group were selected as the research objects and preliminarily deemed blood transitional ingredients. After ion extraction, the ChemSpider database was used to match fragments acquired by the instrument with theoretical fragments. Next, a chemical-composition list was established, including compound name, molecular formula, accurate mass two-dimensional (2D) structure, and other relevant information, by searching the literature. The above information was imported into Progenesis SDF Studio software (Waters) to develop a laboratory database. By combining information from spectrum databases such as MassBank and the National Institute of Standards and Technology (NIST; https://www.nist.gov/data), the ingredients of KLX *in vivo* and *in vitro* were identified.

### Correlation Analysis Between Biomarkers and Serum Constituents

Given the effect of KLX on DR, the correlation coefficient (*r*) was applied to reveal potential effective constituents by plotting the correlation between metabolite markers with serum constituents that originated from TCMs (PCMS) version 1.0 (registration number of copyrights of computer software: 2015SR164324, China); 0.6 ≤ |*r*| <0.7 represented highly correlation, and 0.7 ≤ |*r*| ≤ 1 represented extremely high correlation.

### Statistical Analysis

All statistical analyses were performed using GraphPad Prism 5 (GraphPad Software, Inc., San Diego, CA, United States). Comparisons between two groups were performed by using an unpaired two-tailed Student’s *t*-test. Data are presented as the means ± SEM, and *p* < 0.05 was considered statistically significant.

## Results

### Assessment of Diabetic Retinopathy Mouse Model

**FIGURE 1:**
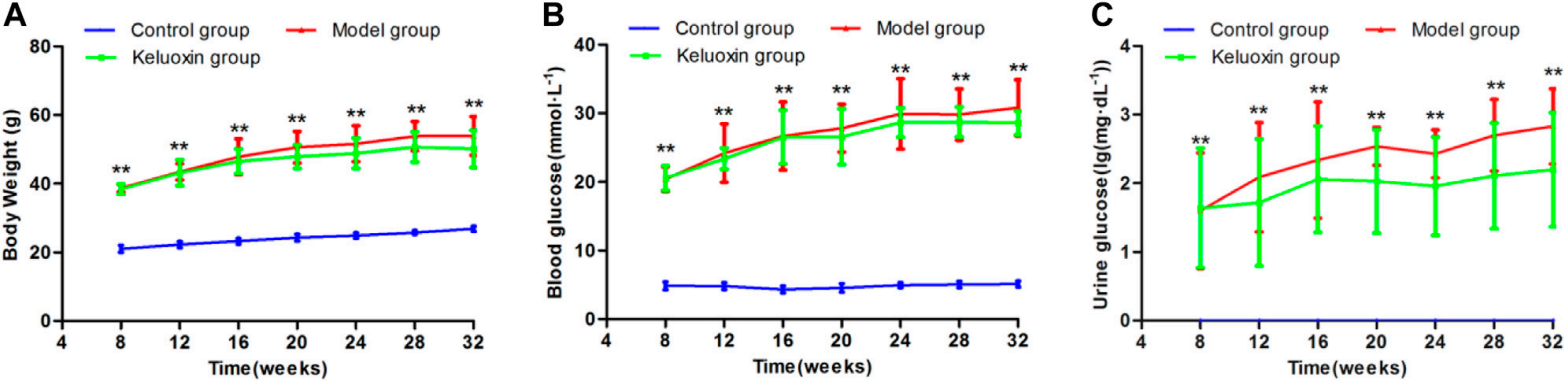
Changes in BW, blood glucose level, and urine glucose level of the control, model, and KLX-treated groups. **(A)** Change in BW during treatment of DR with KLX. **(B)** Change in blood glucose level during treatment of DR with KLX. **(C)** Change in urine glucose level during treatment of DR with KLX. Values are presented as mean ± SEM (*n* = 10 per group). Data were analyzed by Student’s t-test. Model group data were compared with control group data over the same period; **p < 0.01. BW, body weight; KLX, keluoxin; DR, diabetic retinopathy.

Compared with the control group, the body weight (BW) was significantly increased in the model group (Figure 1A). Blood glucose in the model group was significantly higher than that in the control group from 8 weeks of age, at >11.1 mmol/L (Figure 1B). Urine glucose was negative in the control group but positive in the model group (Figure 1C). Based on BW, blood glucose, and urine glucose, we diagnosed these 8-week-old model mice with diabetes.

**FIGURE 2:**
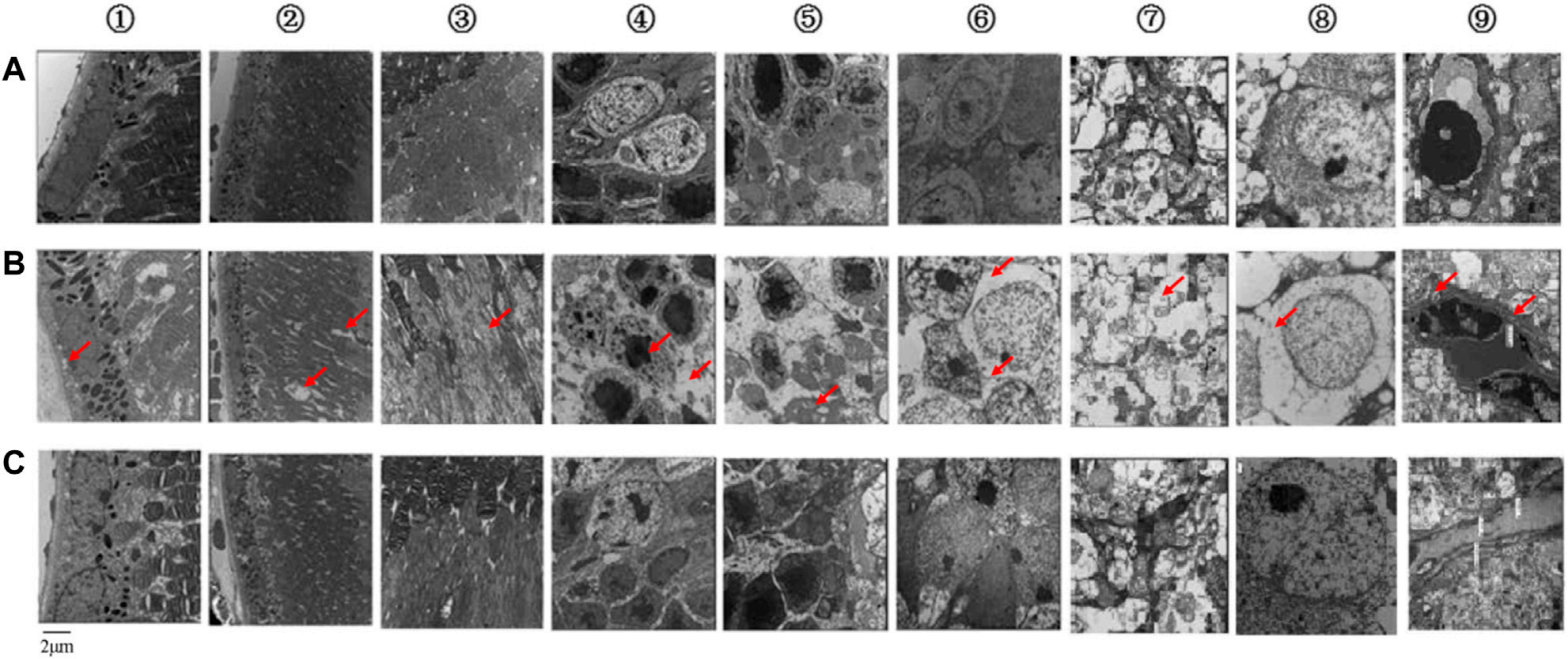
Ultrastructures of each retinal layer and blood vessel as observed by TEM. **(A)** Control group. **(B)** Model group. **(C)** KLX-treated group. KLX alleviated damage to the retinal structure and inhibited thickening of the basement membrane, which prevented progression of DR and reduced damage to visual function. The following are descriptions of results in the KLX-treated group compared with the control and model groups. (A①–C①) RPE cells were arranged regularly, their nuclei were clearer, and the morphology of mitochondria in the cytoplasm was normal. (A②–C②) In the membrane discs of visual cells, structure was relatively complete, with almost no fragmentation or dissolution, relatively neat arrangement, and relatively clear outline. (A③–C③) In the visual cell layer, a few mitochondria showed swelling and vacuolation. (A④–C④) In the ONL, the arrangement of cone and rod cells was more regular, and the extracellular space was narrowed. (A⑤–C⑤) The OPL showed fewer pathological changes of mitochondrial edema and vacuolation. (A⑥–C⑥) In the INL, nuclear chromatin of bipolar cells was evenly distributed, the intercellular space was narrowed, there were more organelles, and the mitochondria were less swollen. (A⑦–C⑦) In the IPL, there were more complete mitochondria, synapses, and synaptic vesicles. (A⑧–C⑧) In the ganglion cells, there were more mitochondria and organelles, less swelling, fewer vacuoles, and clear structure. (A⑨–C⑨) In the retinal capillary vessels, the basement membranes of RCECs and pericytes were thinner, and there were fewer vacuolated mitochondria. TEM, transmission electron microscope; KLX, keluoxin; DR, diabetic retinopathy; RPE, retinal pigment epithelial; ONL, outer nuclear layer; OPL, outer plexiform layer; INL, inner nuclear layer; IPL, inner plexiform layer; RCECs, retinal capillary endothelial cells.

When the mice were 32 weeks old, we observed pathological changes to the ultrastructure caused by diabetes in each layer of the retina. In the control group, retinal pigment epithelial (RPE) cells were arranged regularly, with clear nuclei, mitochondria, and a few pigment granules in the cytoplasm (Figure 2A①). In the model group, the nuclei of RPE cells were not clear. Mitochondria had decreased, and some vacuoles appeared in the cytoplasm (Figure 2B①). In the control group, the membrane discs of the visual cells were intact and arranged neatly, with a clear structure and without fragmentation or dissolution (Figure 2A②). In the model group, these membrane discs were partial and indistinct, with focal dissolution and rupture, widened disc space, disordered arrangement, loose deformation, and mitochondrial degeneration (Figure 2B②). In the control group, the mitochondrial structure was intact in the visual cell layer (Figure 2A③). In the model group, we saw mitochondrial swelling, vacuolation, dissolution, and crista rupture in this layer (Figure 2B③). In the control group, cone and rod cells in the outer nuclear layer (ONL) were arranged regularly, nuclear chromatin was evenly distributed, and extracellular space was tight (Figure 2A④). In the model group, the arrangement of cone and rod cells in the ONL was irregular, the distribution of nuclear chromatin was irregular, and the extracellular space demonstrated edema (Figure 2B④). In the control group, the mitochondrial structure was normal in the outer plexiform layer (OPL; Figure 2A⑤). In the model group, mitochondria showed edema, vacuolation, dissolution, and crista rupture in the OPL (Figure 2B⑤). Horizontal, bipolar, and amacrine cells were successively distributed throughout the inner nuclear layer (INL). In the control group, nuclear chromatin in the INL was evenly distributed, with more organelles, a complete structure, and slight swelling of mitochondria (Figure 2A⑥). In the model group, the cytoplasm of bipolar cells in the INL showed edema, nuclei had condensed, heterochromatin was condensed toward the center, intercellular space was enlarged, organelles were lost, and mitochondria were swollen and vacuolated (Figure 2B⑥). In the control group, the neuronal axons contained a large number of intact mitochondria with slight swelling, and there were more synapses and synaptic vesicles in the inner plexiform layer (IPL; Figure 2A⑦). In the model group, cytoplasm had liquefied and become edematous, mitochondria were swollen and vacuolated, and synapses and synaptic vesicles had decreased in the IPL (Figure 2B⑦). In the control group, ganglion cells were the largest in volume and formed the innermost cells of the retina in a single row. Nuclear chromatin was uniform, while organelles such as polyribosomes and the rough endoplasmic reticulum (ER) structure were clear (Figure 2A⑧). In the model group, the nuclei of ganglion cells were pyknotic, a large number of vacuoles could be seen in the cytoplasm, and the number of organelles was reduced compared with the control group; also, the number of mitochondria decreased, and many of them showed swelling and vacuolation (Figure 2B⑧). In the control group, the thickness of the basement membranes in retinal capillary endothelial cells (RCECs) and pericytes was normal, and the mitochondrial structure of both types of cells was relatively intact (Figure 2A⑨). In the model group, the basement membranes of RCECs and pericytes were thickened locally, and mitochondria in these cells were seriously cavitated, with broken cristae and destruction of the tight junction (Figure 2B⑨). These pathological changes showed that our DR mouse model was successful.

**FIGURE 3:**
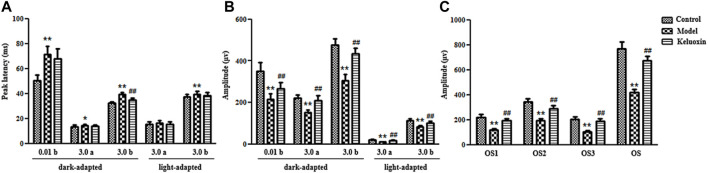
ERGs of the control, model, and KLX-treated groups. **(A)** Peak latencies of b-wave of dark-adapted 0.01 ERG, a-wave of dark-adapted 3.0 ERG, b-wave of dark-adapted 3.0 ERG, a-wave of light-adapted 3.0 ERG, and b-wave of light-adapted 3.0 ERG. **(B)** Amplitudes of b-wave of dark-adapted 0.01 ERG, a-wave of dark-adapted 3.0 ERG, b-wave of dark-adapted 3.0 ERG, a-wave of light-adapted 3.0 ERG, and b-wave of light-adapted 3.0 ERG. **(C)** Amplitude of Ops of dark-adapted 3.0 ERG. Values are presented as mean ± SEM (*n* = 10 per group). Data were analyzed by Student’s *t*-test. Model group was compared with control group; *p < 0.05, **p < 0.01. KLX-treated group was compared with model group data; ^#^ *p* < 0.05. ERG, electroretinography; KLX, keluoxin; Ops, oscillatory potentials.

We evaluated retinal function in the mice using fERG (Figure 3), and the results showed that peak latency was prolonged, and the amplitude was decreased in the model group compared with the control group. The abnormal b-wave of dark-adapted 0.01 ERG meant dysfunction of the rod system, especially bipolar cells connected to rod cells. The abnormal a-wave of dark-adapted 3.0 ERG meant dysfunction of the mixed rod-and-cone system, especially rod and cone cells. The abnormal b-wave of dark-adapted 3.0 ERG also meant dysfunction of the mixed rod-and-cone system, especially bipolar cells connected to rod and cone cells. The abnormal a-wave of light-adapted 3.0 ERG meant dysfunction of the cone system, especially cone cells. The abnormal b-wave of light-adapted 3.0 ERG meant dysfunction of the cone system, especially bipolar cells connected to cone cells. Ops of dark-adapted 3.0 ERG showed that optical-signal (OS) amplitudes OS1, OS2, OS3, and OS were decreased in the model group compared with the control group, which meant abnormal retinal microcirculation. Therefore, the abnormal function of retinal nerve cells showed that our DR mouse model was successfully established when the mice were 32 weeks old.

### Therapeutic Effects of Keluoxins on Diabetic Retinopathy Model Mice

Mice in the KLX-treated group were given KLX for 24 consecutive weeks from the age of 8 weeks. After KLX treatment, BW and levels of both blood glucose and urine glucose were decreased as compared with the model group, which proved that symptoms of diabetes were alleviated by KLX intervention (Figure 1). Retinal-ultrastructure results (Figure 2) for the KLX-treated group showed that the nuclei of RPE cells were clearer and the morphology of cytoplasmic mitochondria was normal as compared with the model group (Figure 2C①). There was almost no fragmentation or dissolution in the partial-membrane discs of the visual cells, whose structure was relatively intact, with a rather neat arrangement and a clear outline (Figure 2C②). A few mitochondria showed swelling and vacuolation in the visual cell layer (Figure 2C③). The arrangement of cone and rod cells in the ONL was more regular, and the extracellular space was narrowed (Figure 2C④). Pathological changes of mitochondrial edema and vacuolation in the OPL were fewer than in the model group (Figure 2C⑤). The nuclear chromatin of bipolar cells in the INL was evenly distributed, intercellular space was narrowed, there were more organelles, and mitochondria were less swollen (Figure 2C⑥). The IPL had great numbers of intact mitochondria and synapses and synaptic vesicles (Figure 2C⑦). The number of mitochondria increased, their swelling and vacuolation decreased, organelles increased, and the structure was clear (Figure 2C⑧). The basement membranes of RCECs and pericytes became thinner, and the number of vacuolated mitochondria decreased (Figure 2C⑨). These results showed that pathological changes of DR could be significantly improved by treatment with KLX.

Our fERG results showed that KLX could significantly shorten peak latency and increase amplitude in the KLX-treated group compared with the model group (Figure 3). The b-wave of dark-adapted 0.01 ERG showed that KLX could improve the function of the rod system, especially of bipolar cells connected to rod cells. The a-wave of dark-adapted 3.0 ERG showed that KLX could improve the function of the mixed rod-and-cone system, especially of rod and cone cells. The b-wave of dark-adapted 3.0 ERG showed that KLX could improve the function of the mixed rod-and-cone system, especially of bipolar cells connected to rod and cone cells. The a-wave of light-adapted 3.0 ERG showed that KLX could improve the function of the cone system, especially of cone cells. The b-wave of light-adapted 3.0 ERG showed that KLX could improve the function of the cone system, especially of bipolar cells connected to cone cells. Ops of dark-adapted 3.0 ERG showed that KLX increased amplitude by a significant degree in the treatment group compared with the model group, which proved that KLX could improve abnormal retinal microcirculation. Therefore, fERG results demonstrated that KLX could improve the function of retinal nerve cells in DR mice.

### Metabolomics Analysis and Biomarker Identification

**FIGURE 4:**
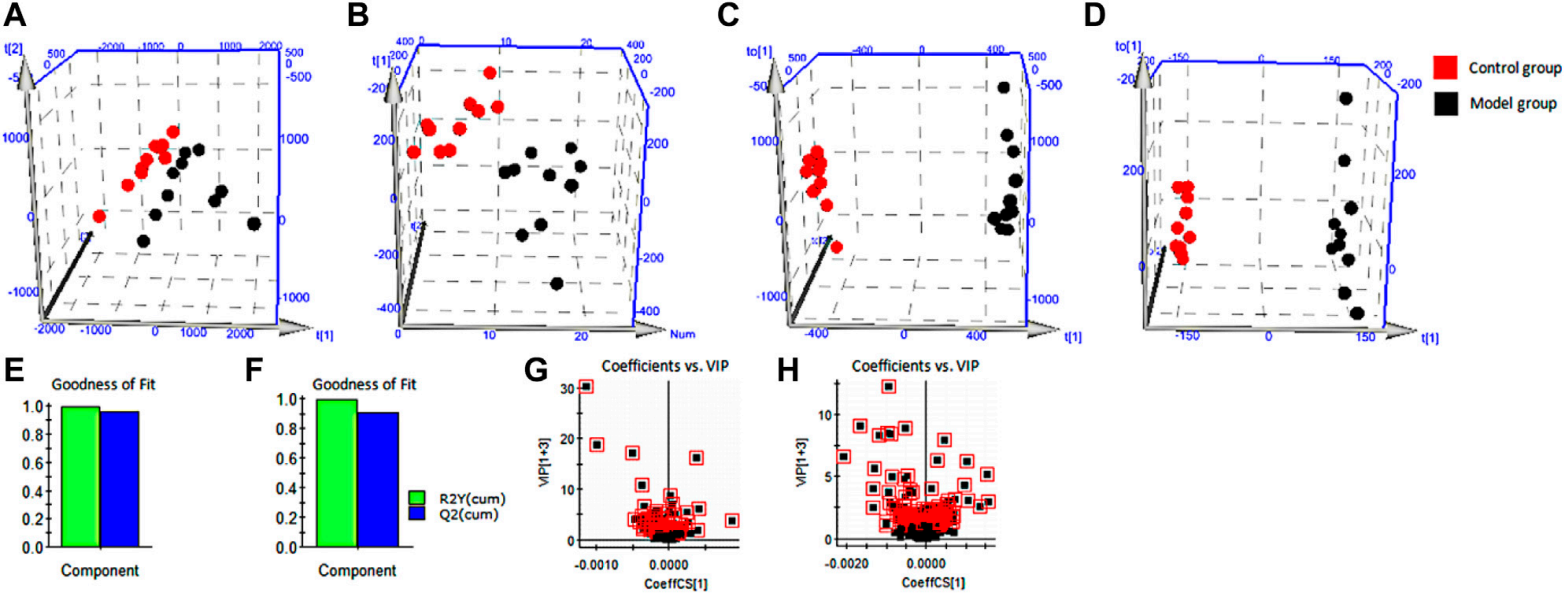
Multivariate statistical analysis. **(A)** PCA score plots for the control group and the model group in positive ion mode. **(B)** PCA score plots for the control group and the model group in negative ion mode. **(C)** OPLS-DA score plots for the control group and the model group in positive ion mode. **(D)** OPLS-DA score plots for the control group and the model group in negative ion mode. **(E)** Evaluation of stable and reliable parameters of OPLS-DA model in positive ion mode. **(F)** Evaluation of stable and reliable parameters of OPLS-DA model in negative ion mode. **(G)** Serum biomarkers in the VIP between the control group and the model group in positive ion mode. **(H)** Serum biomarkers in the VIP between the control group and the model group in negative ion mode. PCA, principal component analysis; OPLS-DA, orthogonal partial least squares discriminant analysis; VIP, variable importance in projection.

We processed blood samples from the control and model groups using the blood metabolomics samples method (Supplementary Material) and acquired metabolomics data by an optimized UPLC-MS condition (Supplementary Material). The base peak ion (BPI) chromatogram of each sample showed blood metabolism profile information (Supplementary Figure S1). Furthermore, we used PCA (Figures 4A,B) and OPLS-DA (Figures 4C,D) to accurately reflect small between-group differences, whose results indicated that the blood metabolism profiles changed significantly. R^2^X, R^2^Y, and Q^2^ were respectively 86.33%, 99.64%, and 96.17% in the positive-ion mode and 53.50%, 99.75%, and 90.37% in the negative-ion mode (Figures 4E,F). This demonstrated that the multidimensional statistical model was stable and reliable. Furthermore, we obtained the VIP value of each metabolite via OPLS-DA and used it to screen biomarkers (Figures 4G,H) such as arachidonic acid, cortisol, and biliverdin (Supplementary Figure S2). Using the abovementioned analytical methods, we successfully identified a total of 64 blood biomarkers (Supplementary Table S1). The histogram (Supplementary Figure S3) and the heatmap of hierarchical-clustering analysis (Supplementary Figure S4) show that levels of 38 biomarkers increased and those of 26 biomarkers decreased in the model group.

### Protective Effect of Keluoxins Against Diabetic Retinopathy Based on Metabolomics

**FIGURE 5:**
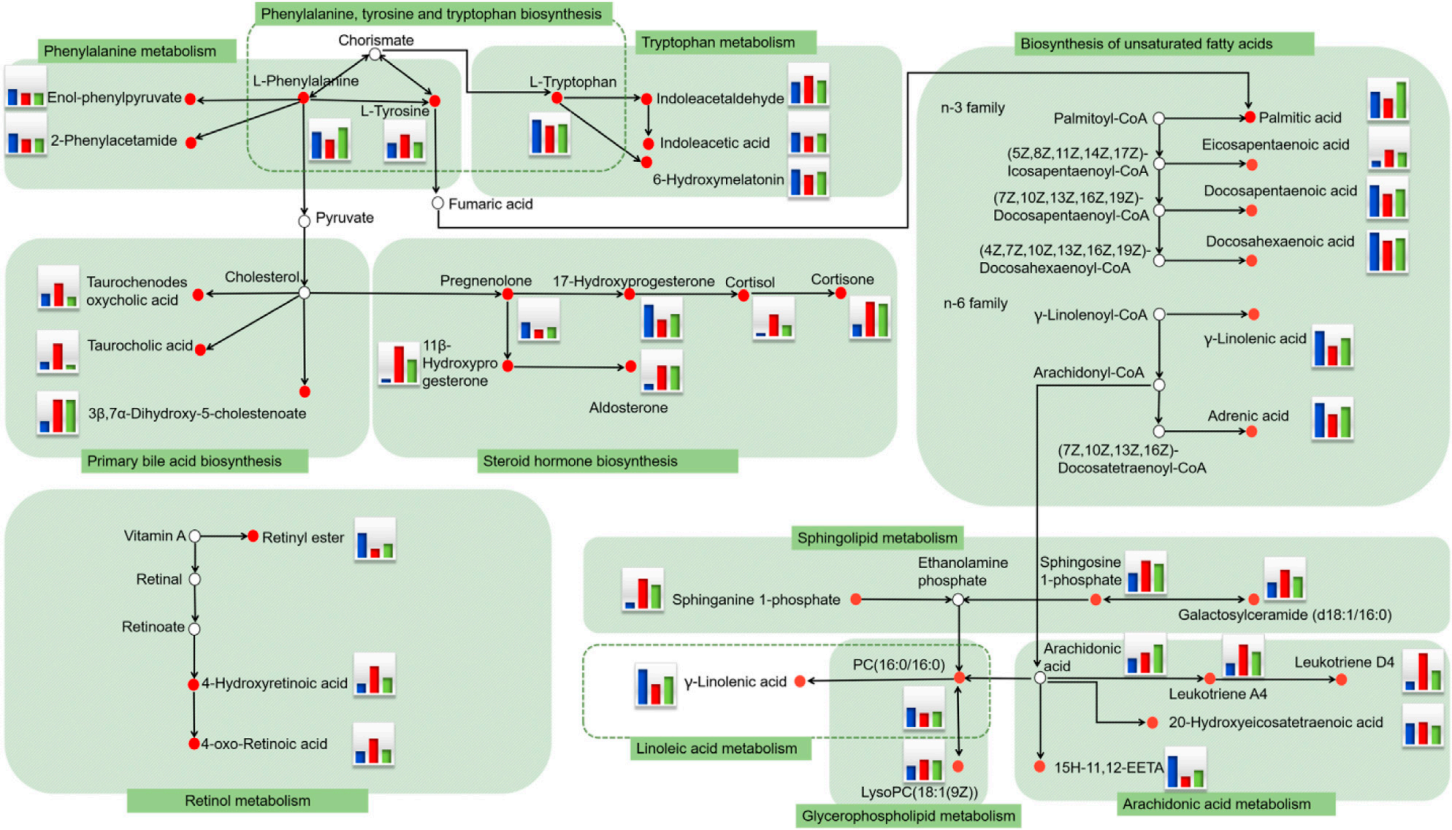
Serum metabolic network of potential DR biomarkers after oral administration of KLX based on the KEGG pathway database. The small red circle represents the biomarker detected in the study. The histogram represents the level of metabolites in the blood. Blue represents the control group, red represents the model group, and green represents the KLX-treated group. DR, diabetic retinopathy; KLX, keluoxin; KEGG, Kyoto Encyclopedia of Genes and Genomes.

We used PCA to analyze expression levels of blood biomarkers in each group, in order to directly reflect differences in metabolic profile. PCA results showed significant metabolic differences among the three experimental groups. The KLX-treated group was more similar to the control group from a vector position, indicating that KLX had a noticeable regulatory effect on the abnormal blood metabolic profiles of DR mice (Supplementary Figure S5). Of the 64 blood biomarkers of DR, KLX could reverse 51 to normal levels. The levels of 29 biomarkers differed to a statistically significant degree between the model group and the KLX-treated group (Supplementary Figure S6). Biomarkers with significant changes mainly include leukotriene D4 and A4 (LTD4 and LTA4), cortisol, 11β-hydroxyprogesterone (11β-OHP), and 15(*S*)-hydroxy-11,12-epoxyeicosatrienoic acid (15H-11,12-EETA). For metabolic-network analysis, we used the pathway analysis tool MetaboAnalyst version 3.0 to synthetically analyze the 51 biomarkers of KLX regulation. We found 25 metabolic pathways related to KLX’s effect on DR (Supplementary Table S2). Six core pathways had *p* < 0.05 and impact >0 associated with this effect: phenylalanine–tyrosine–tryptophan biosynthesis, phenylalanine metabolism, steroid hormone biosynthesis, sphingolipid metabolism, tryptophan metabolism, and arachidonic acid metabolism (Supplementary Figure S7). Then we mapped the metabolic network to clarify the metabolic mechanism by which KLX treated DR (Figure 5). Through key metabolic pathways, we discovered 15 biomarkers—l-tyrosine, l-tryptophan, indoleacetaldehyde, indoleacetic acid, l-phenylalanine, 6-hydroxymelatonin (6-Ω), LTD4, LTA4, cortisol, 11β-OHP, sphingosine 1-phosphate (S1P), 20-hydroxyeico-satetraenoic acid (20-HETE), gamma-linolenic acid (GLA), galactosylceramide (d18:1/16:0), and 15H-11,12-EETA—that we deemed core biomarkers of KLX against DR.

### Potential Targets of Keluoxins Against Diabetic Retinopathy Mice

**FIGURE 6:**
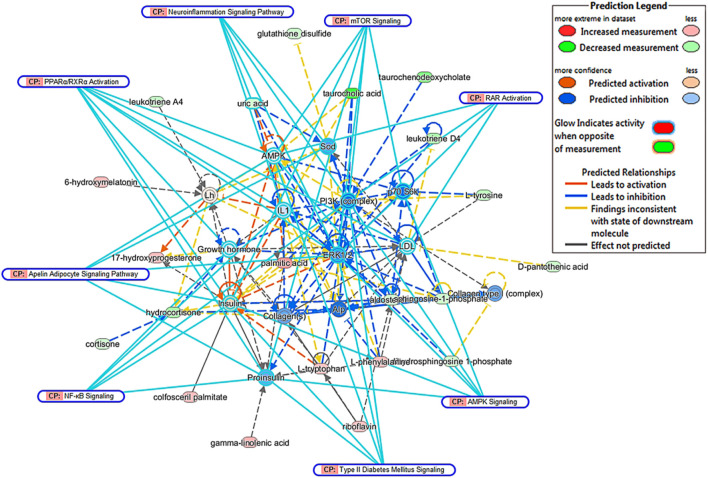
Comprehensive network of blood biomarkers of KLX regulating DR based on the IPA omics platform. Red ellipse indicates increase in metabolite intensity regulated by KLX. Green ellipse indicates decrease in metabolite intensity regulated by KLX. Yellow ellipse indicates molecules activated. Blue ellipse indicates molecules inhibited. Yellow line represents the activation relationship. Blue line represents inhibition relationship. Continuous line represents direct relationships. Dotted line represents indirect relationships. KLX, keluoxin; DR, diabetic retinopathy; IPA, Ingenuity Pathway Analysis.

**FIGURE 7:**
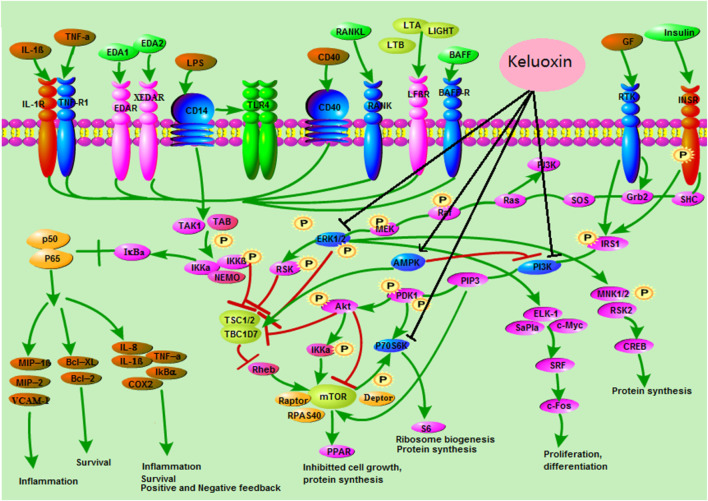
Core targets and signaling pathways of KLX’s regulation of DR. KLX could inhibit ERK1/2, PI3K, and p70 S6K and activate MAPK to influence the MAPK signal transduction, mTORC1 signaling, and NF-κB signaling pathways, which delayed the progression of DR. KLX, keluoxin; DR, diabetic retinopathy.

To find the metabolic mechanism by which KLX treated DR, we used the IPA omics-platform (Ingenuity, Redwood City, CA, United States) to find the key bioinformatics network. IPA biological-network analysis uncovered 12 biomarkers associated with the pathways by which KLX treated DR: aldosterone, S1P, LTD4, l-tryptophan, palmitic acid, l-phenylalanine, taurocholic acid, l-tyrosine, 6-Ω, LTA4, GLA, and 17-hydroxyprogesterone (17-OPH; Figure 6). There were eight canonical pathways related to the treatment of DR via KLX: mammalian target of rapamycin (mTOR) signaling, adenosine monophosphate (AMP)-activated protein kinase (AMPK) signaling, neuroinflammation signaling, peroxisome proliferator-activated receptor alpha (PPAR-α)/retinoid X receptor alpha (RXR-α) activation, apelin adipocyte signaling, type 2 diabetes mellitus (T2DM) signaling, nuclear factor κ-light-chain-enhancer of activated B cells (NF-κB) signaling, and retinoic acid receptor (RAR) activation. The corresponding core targets were AMPK, extracellular signal-regulated protein kinase1/2 (ERK1/2), phosphatidylinositol-3-kinase (PI3K), and protein 70 S6 kinase (p70 S6K; Figure 7).

### Effective Constituents of Keluoxins Against Diabetic Retinopathy

**FIGURE 8:**
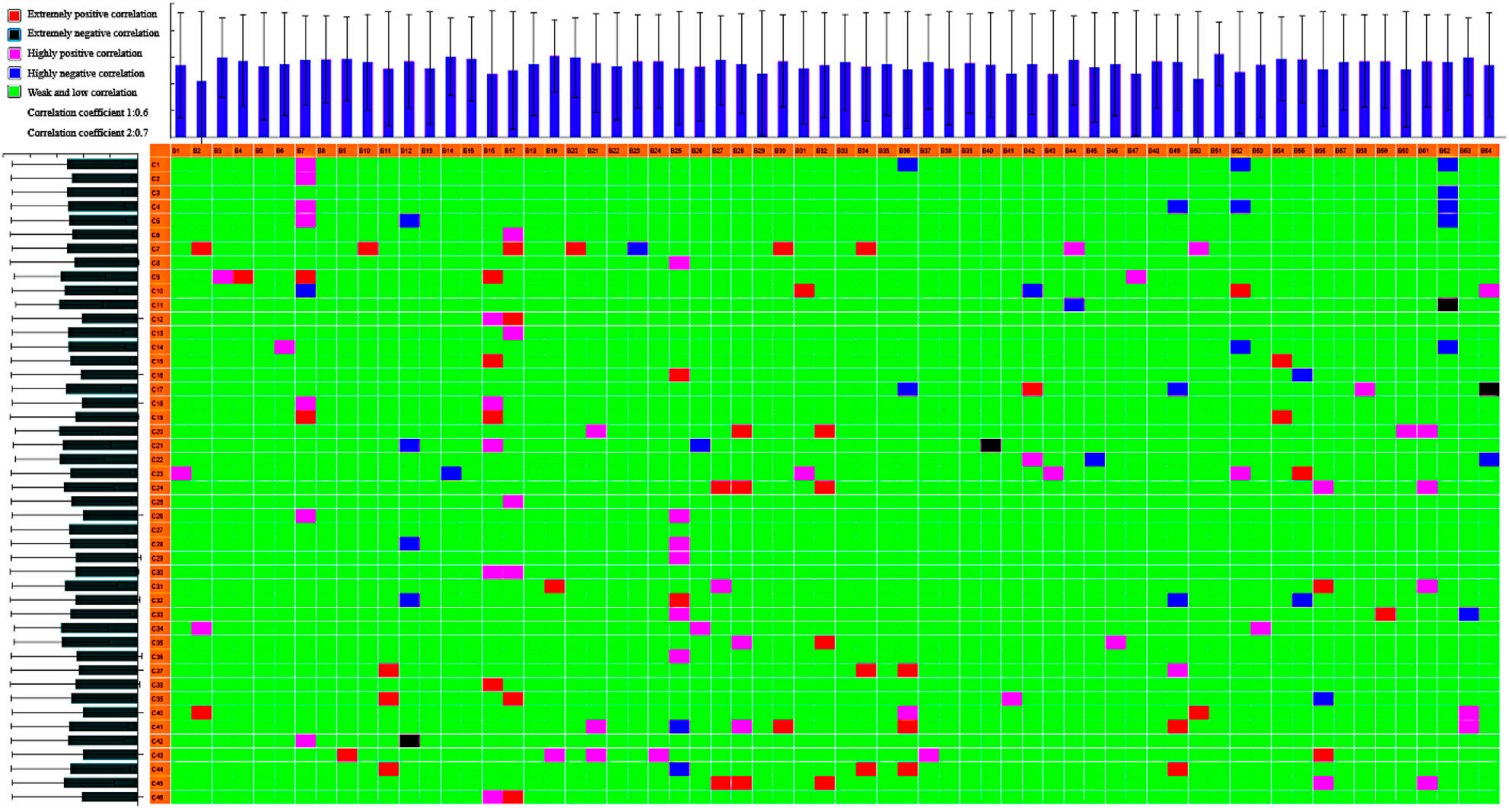
Heatmap of correlations between biomarkers and serum constituents in KLX. B1–B64 show the biomarker (listed in Supplementary Table S1); C1–C45 show the serum component (listed in Supplementary Table S5). R1, 0.6; R2, 0.7. Extremely positive correlation; extremely negative correlation; highly positive correlation; highly negative correlation; weak/low correlation. KLX, keluoxin.

We used UPLC-quadrupole/TOF-MS (UPLC-Q/TOF-MS) with DBS triggering IDA of tandem MS for rapid discovery and comprehensive characterization of multiple KLX constituents. Molecular formulas of compounds were determined according to the accurate mass. Then, the actual spectra with m/z information and theoretical spectra generated by the database were matched and scored, and the literature was perused for metabolite information. We identified 212 compounds of KLX, including 140 positive ions (Supplementary Table S3) and 72 negative ions (Supplementary Table S4). Seventy ingredients were from Astragali radix, 30 from Pseudostellariae radix, 55 from Ligustri lucidi fructus, 36 from Lycii fructus, 104 from Rhei radix et rhizome, and 13 from Hirudo. Some components existed in more than one TCM simultaneously. We identified 46 blood transitional ingredients of KLX, which included isoformononetin, ononin, biochanin A, raffinose, 10-hydroxyoleuropein, 3-glucogallic acid, emodin, rhein, and chrysophanol (Supplementary Table S5). Correlation analysis of these blood transitional components and endogenous biomarkers using our chinmedomics strategy found that 11 components were the effective constituents of KLX against DR (Figure 8): emodin, rhein, astragaloside IV (AS-IV), chrysophanol, chrysophanol-8-*O*-β-d-glucopyranoside, aloe-emodin, aloin, biochanin A, 10-hydroxyoleuropein, 3,4′,7-trihydroxyflavone, and 3′-*O*-methyl-(−)-epicatechin 7-*O*-glucuronide (3′ME7G).

## Discussion

We have established a DR model in C57BL/KsJ-db/db mice with spontaneous diabetes and treated them with KLX. KLX could reduce BW as well as blood glucose and urine glucose levels of these mice, shorten peak latency, significantly increase dark-adapted and light-adapted amplitudes, and improve the function of the rod-and-cone system and retinal microcirculation. The retinal ultrastructure showed that KLX could inhibit the thickening of the basement membrane and damage to the retinal structure. The average metabolic process of cells requires a continuous energy supply, and mitochondria are the primary sites of oxidative phosphorylation and adenosine triphosphate (ATP) synthesis in cells. AMPK and mTOR regulate the mitochondrial function and energy balance in cells. In our study, we saw different degrees of mitochondrial damage to the retinas of DR mice. KLX might improve mitochondrial function and retinal damage by regulating AMPK/mTOR.

Metabolomics strategy has been used for discovering biomarkers (Liang et al., 2014; Liang et al., 2015; Liang et al., 2016; Liang et al., 2017; Zhang et al., 2016), evaluating the pharmacological effects (Qiu et al., 2017; Zhang et al., 2018; Zhang A. H. et al., 2019), and revealing the metabolic mechanisms and potential targets for natural products (Zhang A.-H. et al., 2019; Qiu et al., 2020; Zhang et al., 2020). In this work, by assessing biomarkers of DR regulated by KLX in metabolomics, we further locked six core metabolic pathways, including phenylalanine–tyrosine–tryptophan biosynthesis, phenylalanine metabolism, steroid hormone biosynthesis, sphingolipid metabolism, tryptophan metabolism, and arachidonic acid metabolism. Twelve biomarkers involved in these pathways were closely related to the KLX effect on DR. Arachidonic acid metabolism plays an important role in T2DM and its complications, its metabolic pathways *in vivo* include cyclooxygenase (COX), lipoxygenase (LOX), and cytochrome P450 (CYP450) (Wang T. et al., 2019). LOX-derived eicosanoids, including LTs and HETEs, and COX-derived metabolites are important factors causative to oxidative stress and retinal microvascular dysfunction (Wang M. H et al., 2020). The relationship between EETs and insulin resistance (IR) shows that EETs can stimulate insulin secretion of islet cells, improve glucose homeostasis, inhibit apoptosis of islet cells, and play a role in regulating blood glucose (Xu et al., 2010). 20-HETE is a bioactive lipid mediator with potent effects on the vasculature, and its levels of plasma and urine are elevated in hypertension, obesity, and metabolic syndrome (Rocic and Schwartzman 2018). ERK1/2 of MAPK is a component of 20-HETE regulated ischemia-induced angiogenesis (Zhou et al., 2018). The biomarker panel consisting of 12-HETE and 2-piperidone can be used for DR diagnosis (Xuan et al., 2020). KLX can partly regulate arachidonic acid metabolism by decreasing LTA4, LTD4, and 20-HETE levels and increasing 15H-11,12-EETA (HEETA) levels to reduce inflammation and inhibit endothelial cell proliferation. In the catabolism of tryptophan, a variety of physiologically active molecules are generated that are of great significance to the nervous system, growth and development, and various chronic and malignant diseases. The concept of DR as a microvascular disease has evolved, and neurodegeneration plays a significant role (Simo et al., 2018). Retinal neurodegeneration may precede the established clinical and morphometric vascular changes (Sohn et al., 2016). The imbalance of tryptophan metabolism has a serious effect on DR and reduces the integrity of the retinal neuron unit. Tryptophan is a potential marker of DR progression in T2D patients (Yun et al., 2020) and DR is mainly associated with tryptophan metabolism (Wang X. et al., 2020). Abnormal levels of tryptophan are highly correlated with T2DM and its complications (Gong et al., 2019). KLX could regulate tryptophan metabolism by increasing levels of l-tryptophan, indoleacetic acid, and 6-Ω and decreasing the level of indole acetaldehyde to protect the nervous system. *In vivo*, most l-phenylalanine is oxidized to tyrosine by phenylalanine hydroxylase. l-Phenylalanine synthesizes important neurotransmitters and hormones together with l-tyrosine, which is involved in glucose and lipid metabolism. Phenylalanine stimulates the release of glucagon-like peptide-1 (GLP-1) and peptide tyrosine–tyrosine (PYY), decreases plasma ghrelin, stimulates the release of insulin, improves glucose tolerance in rats, and affects the development of diabetes (Alamshah et al., 2017). In Chinese adults, tyrosine > 46 μmol/L was associated with increased odds of T2DM. In Chinese adults, plasma tyrosine levels > 46 μmol/L were related to markedly increased odds of T2DM (Li et al., 2019). KLX could regulate phenylalanine metabolism by increasing levels of l-phenylalanine and decreasing those of l-tyrosine to regulate DR. Hypothalmic–pituitary–adrenal activity is enhanced in diabetic patients with chronic complications, and cortisol level is significantly related to DR and neuropathy (Chiodini et al., 2007). Aldosterone synthase inhibition may be a potential treatment for retinal neovascularization (Deliyanti et al., 2012). KLX could regulate the steroid biosynthesis pathway by decreasing cortisol and aldosterone levels to control DR. Unsaturated fatty acids are involved in many metabolic processes; for instance, ω-6 and ω-3 have anti-inflammatory effects in cardiovascular disease and metabolic syndrome (Tortosa-Caparros et al., 2017). A propensity score matching-based case–control study shows elevated five *n*-6 polyunsaturated fatty acids including GLA, which were independent protective factors of DR (Li et al., 2020). The sectional study of the KAMOGAWA-DM cohort study shows low circulating dihomo-gamma-linolenic acid negatively associated with DR (Okamura et al., 2021). ω-3 fatty acids can prevent DR (Behl and Kotwani 2017), and docosahexaenoic acid (DHA) alleviates retinal oxidative stress and inflammation in retinal diseases (Saenz de Viteri et al., 2020; Suzumura et al., 2020). KLX could regulate the biosynthesis pathway of unsaturated fatty acids by increasing GLA and DHA levels to protect nerves and reduce inflammation.

The potential targets of KLX against DR as predicted by IPA were AMPK, ERK1/2, p70 S6K, and PI3K. The ERK-related intracellular signal transduction pathway is considered the classic MAPK signal transduction pathway. Receptor tyrosine kinase (RTK) and certain growth factors (GFs) can activate the ERK signal transduction pathway. GFs binding to specific receptors of the cell membrane can cause the receptors to form a dimer, and the dimerization receptor can activate its own RTK. The phosphorylated tyrosine on the receptor binds to the Src Homology 2 (SH2) of the cell membrane’s GF receptor-bound protein 2 (Grb2). The SH3 of Grb2 binds to Son of Sevenless (SOS) of the guanosine monophosphate (GMP) exchange factor, which dissociates the guanosine diphosphate (GDP) of small guanine protein-Rac and binds guanosine triphosphate (GTP), thus activating Ras. Activated Ras further binds to the N-terminal of Raf-1 to activate Raf-1, which in turn can phosphorylate two regulatory serines of mitogen-activated protein kinase kinases 1 and 2 (MEK1/MEk2), thus activating MEKs. MEKs, which are bispecific kinases, can phosphorylate serine/threonine and tyrosine to ultimately activate ERK1 and ERK2 in a highly selective manner. ERKs receive upstream cascade reaction signals and can transfer them into the nucleus. KLX inhibited ERK1/2 of the MAPK signal transduction pathway to delay the progression of DR. Activation of PI3K was involved in the substrate close to the inner side of its plasma membrane. GF and signal transduction complexes, including fibroblast GF (FGF), VEGF, hepatocyte GF (HGF), angiopoietin (Ang1), and insulin, could initiate activation of PI3K. These factors activated RTK, which led to autophosphorylation. The phosphorylated residue on the receptor provided a docking site for the PI3Kp85 subunit. When insulin activated insulin receptor (INSR), insulin receptor substrate (IRS) promoted the binding of PI3K. Activation of PI3K produced the second messenger phosphatidylinositol (3,4,5)-trisphosphate (PtdIns(3,4,5)P3(PIP3) on the plasma membrane; PIP3 bound to protein kinase B (Akt) and protein kinase phosphoinositide-dependent kinase1 (PDK1) and promoted the activation of Akt by phosphorylating its Ser308. Finally, Akt activated mTOR complex 1 (mTORC1). The downstream effectors of mTORC1 were mainly S6K and PPAR. mTORC1 was affected by amino acids, oxygen, energy levels, and GFs, which mainly promote protein synthesis, fat production, energy metabolism, inhibition of autophagy, and lysosome formation. The phosphorylation of tuberous sclerosis complex-1 or 2 (TSC1/2) by Akt could prevent negative regulation of Ras homolog enriched in the brain (Rheb), which enriched Rheb to activate mTORC1. These effects could activate protein translation and enhance cell growth. KLX inhibited PI3K and p70 S6K of mTORC1 to delay the progression of DR.

AMPK is an evolutionarily conserved serine/threonine kinase that is a major regulator of energy homeostasis. Activation of AMPK to reduce oxidative stress and regulate metabolic homeostasis in retinal neurons or glia can slow retinal cell death and induce protection (Xu et al., 2018). AMPK activator promotes neuroprotection and vision protection, stabilizing ATP levels by preserving cytochrome *c* oxidase activity (Kawashima et al., 2020). LKB1 and AMPK play a role in rod photoreceptors, and its loss leads to abnormal axonal contraction, postsynaptic dendrite elongation, and heterotopic synapse formation (Samuel et al., 2014). ERKs, including ERK1 and ERK2, are the key to transmitting signals from surface receptors to the nucleus. ERK, a member of the MAPK family, is activated by MAPK kinase (MAPKK). ERK1/2 signal transduction can regulate GFs, cytokines, and hormones, which is the main way to control gene expression. ERK1/2 signaling is not limited to the nucleus and also can target the substrate outside the nucleus to control metabolism, mitochondrial fission, and cell survival. Inhibiting the ERK1/2–NF-κB signaling pathway mediated by high glucose can reduce retinal inflammation caused by microglia and alleviate the symptoms of DR (Zhang et al., 2019). Activation of the VEGF-A/ERK/PLA2 Axis mediates early glucose-induced damage in retinal endothelial cells (Giurdanella et al., 2020). A small-molecule inhibitor of vascular permeability and angiogenesis interacts with MEK1 and suppresses a pERK-FosB/ΔFosB-VCAM-1 axis (Li et al., 2020). P70 S6K is the first signal transduction element inhibited by mTOR in mammalian cells, as well as a downstream effector of mTORC1 (Kelly et al., 2019); it participates in many physiological processes, including cell survival, protein synthesis, and mRNA splicing, by phosphorylating various substrates (Tetsuo et al., 2019). S6K1 absence leads to hypoinsulinemia, glucose intolerance, and β-cell disorder (Um et al., 2004). PI3K δ becomes a new target for DR therapy (Wu et al., 2020). PI3Kβ regulates pericyte proliferation and maturation to govern vascular remodeling (Figueiredo et al., 2020). Regulating ROCK1/PTEN/PI3K/Akt/VEGF pathway can inhibit the proliferation of high glucose-induced human RCECs (Zhou Q et al., 2020).

To further reveal the constituents of KLX that were effective against DR, we screened 11 components using our chinmedomics strategy. These had a wide range of pharmacological effects. Emodin ameliorates cisplatin-induced nephrotoxicity through modulating the AMPK/mTOR signaling pathways and activating autophagy (Liu et al., 2016); it improves glucose metabolism and regulates IR by downregulation of miR-20b and thereby upregulation of SMAD7 (Xiao et al., 2019). Rhein could inhibit autophagy by regulating AMPK-dependent mTOR signaling pathways and Erk and p38 MAPK signaling pathway (Tu et al., 2017); it protects pancreatic β-cells against apoptosis by blocking hyperglycemia-induced dynamin-related protein 1 (Drp1) expression and stabilizing mitochondrial morphology (Liu et al., 2013). AS-IV protects RPE cells of diabetic rats from apoptosis by decreasing p-PI3K/PI3K, p-AKT/AKT, and p-p70S6K1/p70S6K1 and increasing miR-128 expression (Wang T. et al., 2020). It protects high glucose-induced endothelial dysfunction via inhibiting the P2X7R-dependent p38 MAPK signaling pathway (Leng et al., 2020). Aloe-emodin and aloin have antiglycosylation and antiradical properties (Froldi et al., 2019). Aloin inhibits swelling of retinal Müller cells, which is associated with retinal edema and neuronal degeneration (Jung and Kim 2018). The protection of aloin on oxygen–glucose deprivation (OGD) induced apoptosis by inhibiting OGD-induced OS and caspase activity and protecting mitochondrial function in PC12 cells (Chang et al., 2016). Biochanin A protects neurons by inhibiting microglia activation and neuronal apoptosis (Sarfraz et al., 2020) and enhances PI3K/Akt/mTOR signaling and beclin-1 to protect nigral dopaminergic neurons (El-Sherbeeny et al., 2020). It affects carbohydrate metabolism, β-cell function, insulin sensitivity, and IR in the treatment of diabetes mellitus and its complications (Ahmed et al., 2020). 10-Hydroxyl-oleuropein, the active glycoside, protects the red blood cell membranes from resisting hemolysis (Ouyang et al., 2003). 3′,4′,7-Trihydroxyflavone prevents apoptotic neuronal cell death via the MAPKs and PI3K/Akt pathways (Kwon et al., 2015). 3′ME7G, a metabolite of (−)-epicatechin in blood, retains the antioxidative activity (Natsume et al., 2008). With pharmacological effects such as anti-OS, anti-inflammation, neuroprotection, and inhibition of vascular proliferation, these components should be the effective constituents of KLX against DR.

In this study, we used metabolomics technology to characterize metabolic profiles of DR mice at the level of endogenous small-molecule metabolism and to find biomarkers. Then, we used these profiles and biomarkers as parameters for evaluating the overall biological effect of KLX against DR. At the same time, we pinpointed the active ingredients responsible for KLX’s effect against DR by the serum pharmacochemistry method. Endogenous biomarkers were correlated with exogenous ingredients to clarify the effective constituents and mechanism of KLX. The blood metabolic pathways involved in KLX’s treatment of DR were phenylalanine–tyrosine–tryptophan biosynthesis, phenylalanine metabolism, steroid hormone biosynthesis, sphingolipid metabolism, tryptophan metabolism, and arachidonic acid metabolism; the main pathways were mTOR signaling, AMPK signaling, PPAR-α/RXR-α activation, and NF-κB signaling. Core blood biomarkers were LTD4, LTA4, l-tryptophan, 6-Ω, l-phenylalanine, l-tyrosine, and GLA; core targets were AMPK, ERK1/2, PI3K, and p70 S6K. Correlation analysis showed that emodin, rhein, AS-IV, chrysophanol, chrysophanol-8-*O*-β-d-glucopyranoside, aloe-emodin, aloin, biochanin A, 10-hydroxyoleuropein, 3,4′,7-trihydroxyflavone, and 3′ME7G were highly correlated with the effect of KLX. However, the activities and related pathways of these compounds need further experimental analysis and verification. The effectiveness, mechanism, and effective constituents of KLX against DR as shown by our chinmedomics strategy could provide a scientific basis for new indications of KLX.

## Data Availability

The original contributions presented in the study are included in the article/Supplementary Material, Further inquiries can be directed to the corresponding author.
