# Supplementary material for: Chinmedomics Strategy for Elucidating the Pharmacological Effects and Discovering Bioactive Compounds From Keluoxin Against Diabetic Retinopathy
Source: Front Pharmacol. 2022 Mar 31;13:728256. doi: 10.3389/fphar.2022.728256 (PMC9008273; doi:10.3389/fphar.2022.728256)
Supplement: Supplementary file 1 [file DataSheet1.docx]

Supplementary Materials

Fig. S1. UPLC-Q/TOF-MS BPI of serum samples from mice in the control and model groups.

Fig. S2.MS/MS spectral information and fragmentation pathway process.

Fig. S3. Content change in serum biomarkers from mice in the control and model groups.

Fig. S4.Heatmap of serum biomarkers from mice in the control and model groups.

Fig. S5. PCA score plots for the control, model and KLX-treated groups.

Fig. S6. Content changesin serum biomarkers from mice in the control, model and KLX-treated groups.

Fig. S7. Key metabolic pathways of potential DR biomarkers in mouse serum after oral administration of KLX based on MetPA analysis.

Table S1. Potential blood biomarkers of DR model mice based on UPLC-Q/TOF-MS analysis.

Table S2. Critical metabolic pathways of potential DR blood biomarkers after oral administration of KLX based on MetPA analysis.

Table S3. Components of KLX detected by UPLC-Q/TOF-MS in positive-ion mode.

Table S4. Components of KLX detected by UPLC-Q/TOF-MS in negative-ion mode.

Table S5. Blood transitional components of KLX detected by UPLC-Q/TOF-MS in positive- and negative-ion modes.


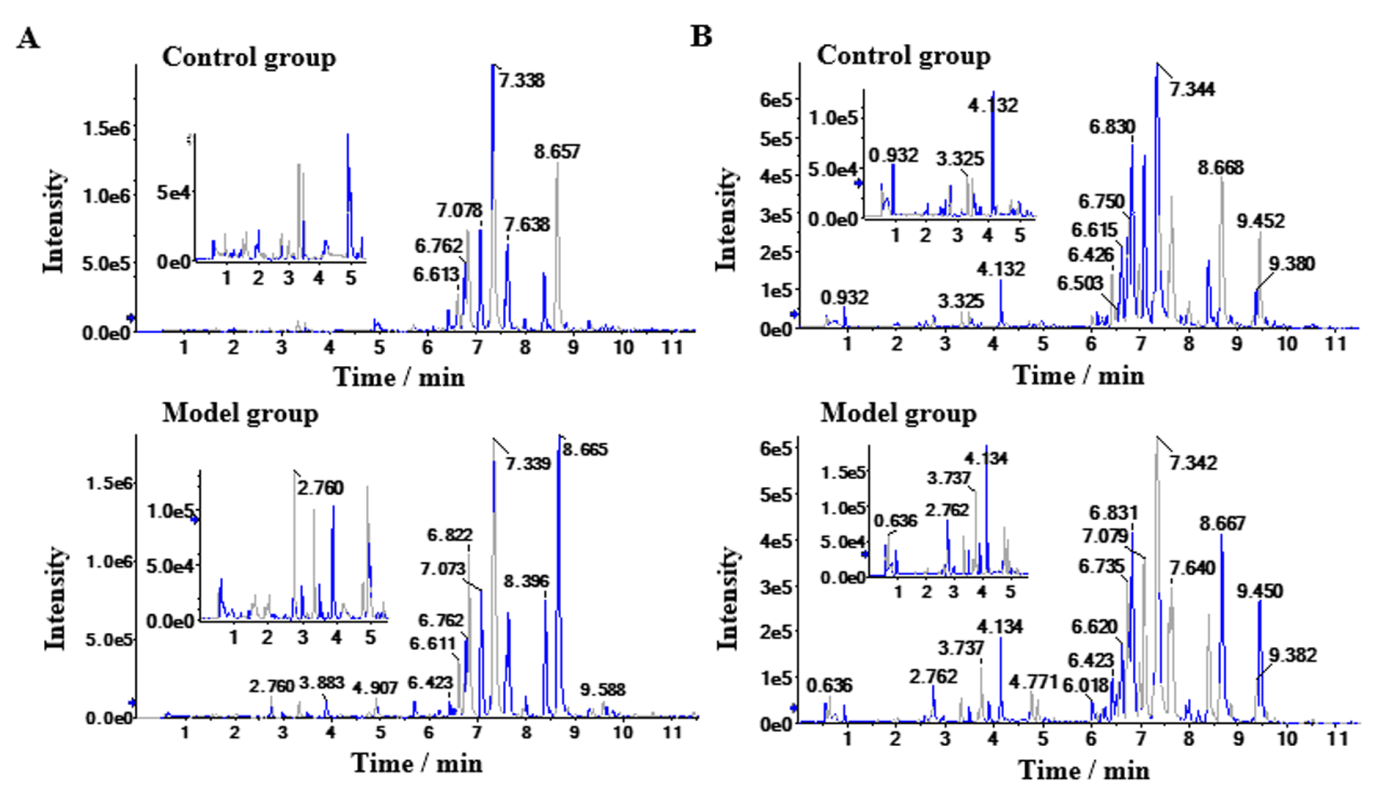


**Fig. S1. UPLC-Q/TOF-MS BPI of serum samples from mice in the control and model groups.**

(A)Positive-ion mode. (B) negative-ion mode.


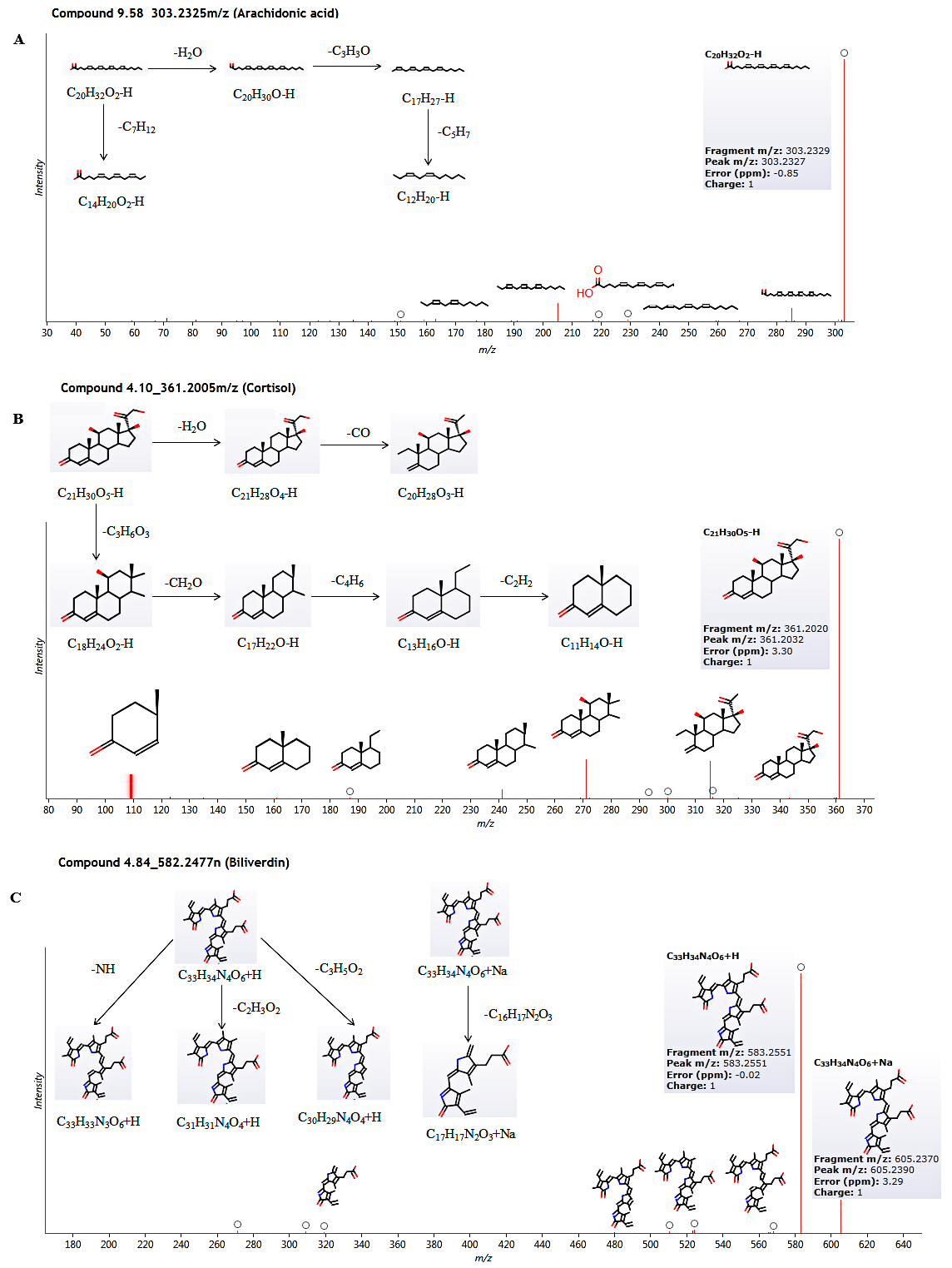


**Fig. S2.MS/MS spectral information and fragmentation pathway process.**

(A) arachidonic acid. (B) cortisol. (C) biliverdin.


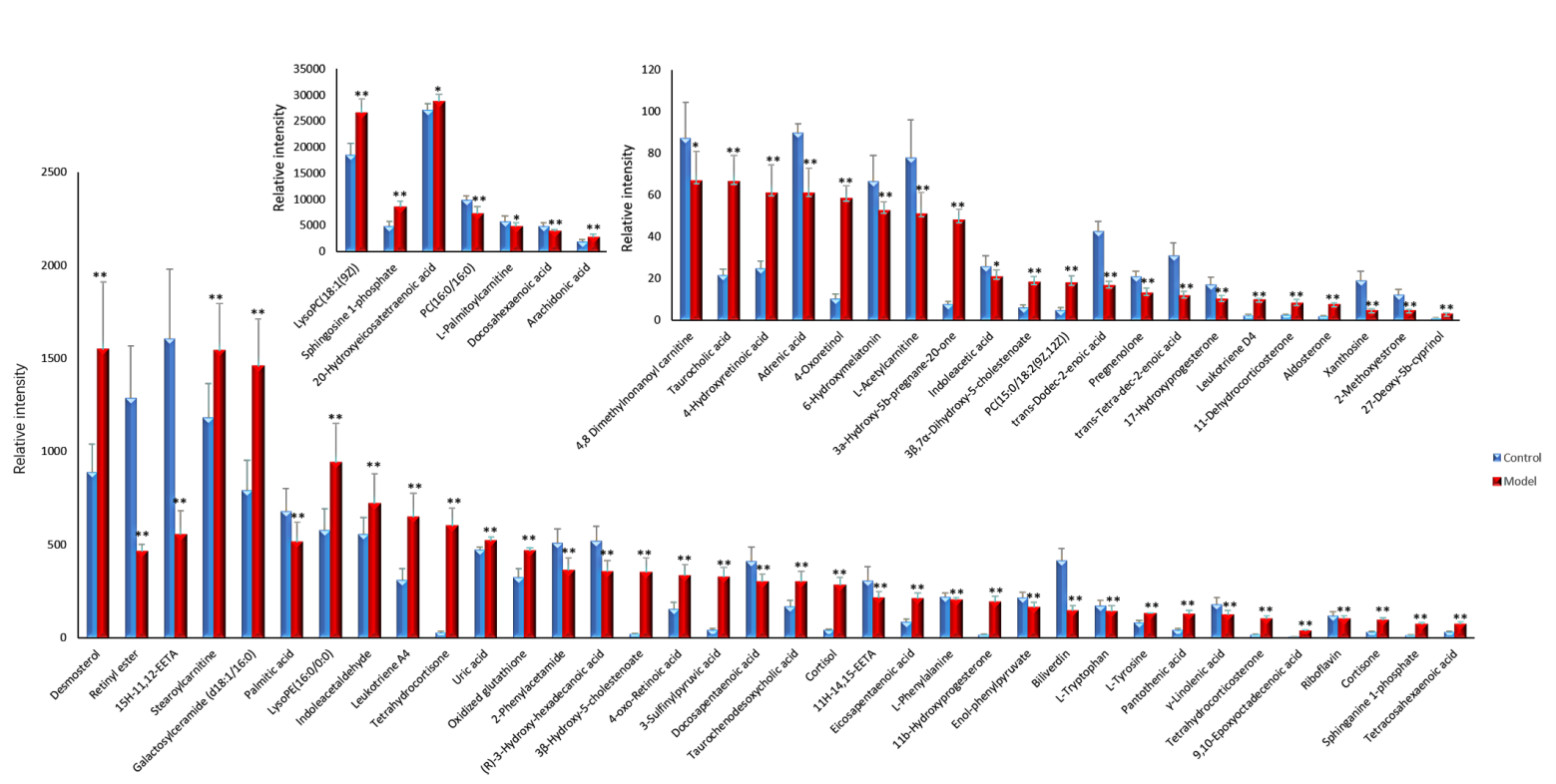


**Fig. S3. Content change in serum biomarkers from mice in the control and model groups.**

Values are presented as mean ±SEM. (n=10 per group). Data were analyzed by Student’s *t*-test. Model group data were compared with control group data; *P < 0.05, **P < 0.01.

**
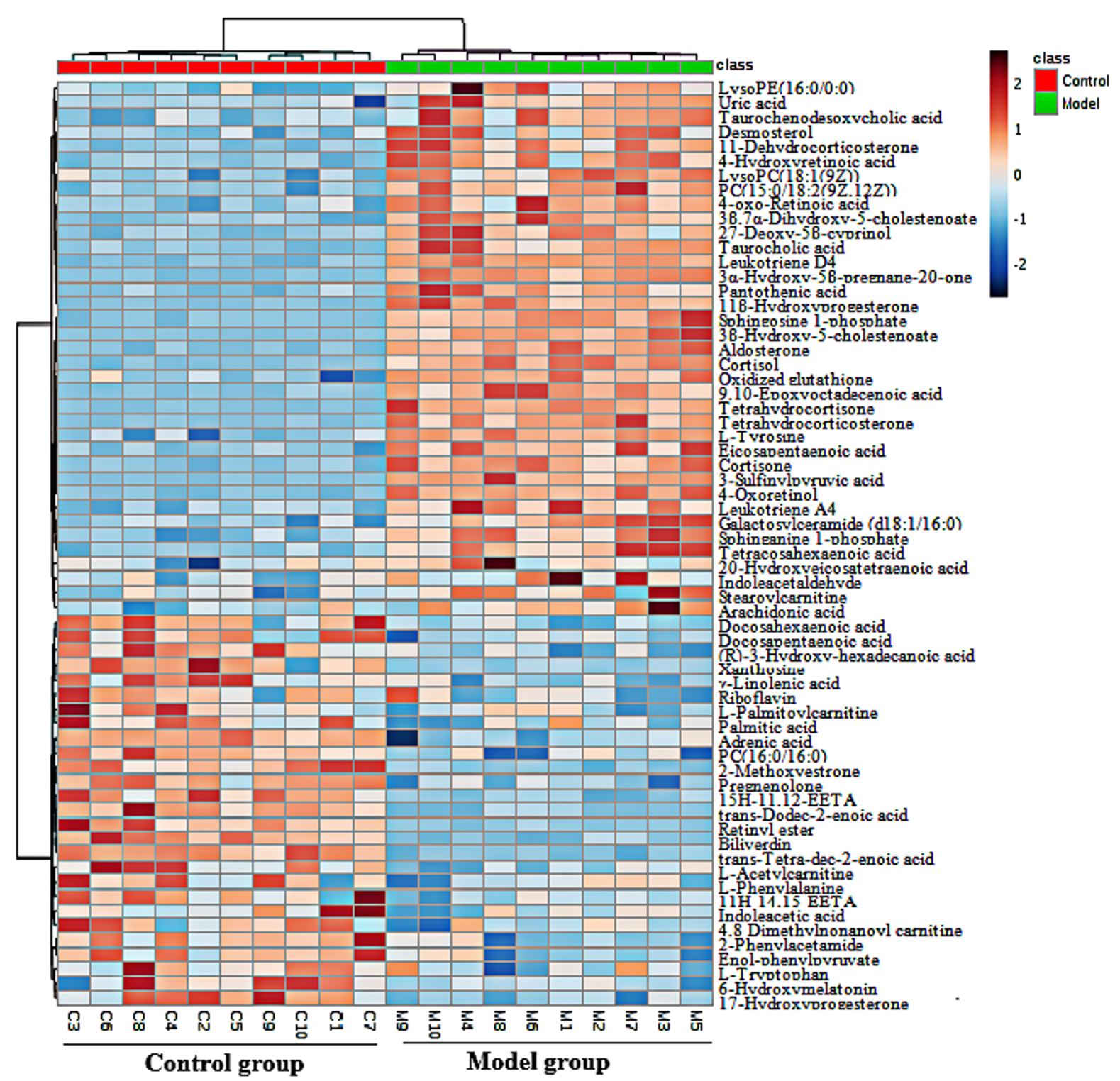
**

**Fig. S4. Heatmap of serum biomarkers from mice in the control and model groups.**

Expression of DR biomarkers was analyzed by hierarchical clustering. The same group samples appeared in the same clusters, and metabolites at the similar levels gathered in the same clusters. Red represents the upregulated metabolites; blue represents downregulated metabolites. The darker color, the more significant difference. Levelsof 38 biomarkers increased, while those of 26 biomarkers decreased.

**
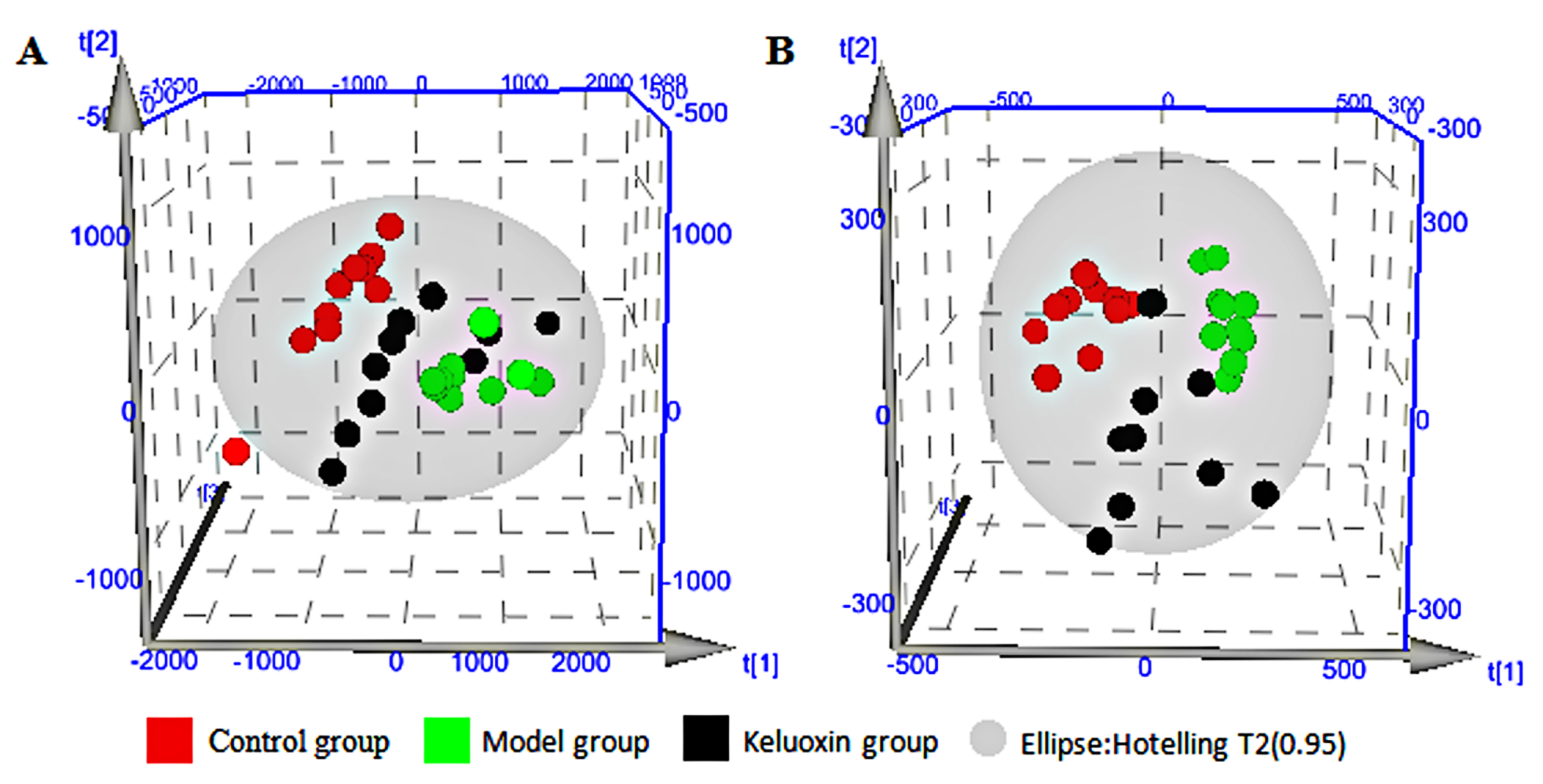
**

**Fig. S5. PCA score plots for the control, model and KLX-treated groups.**

(A) Positive-ion mode. (B) negative-ion mode.

**
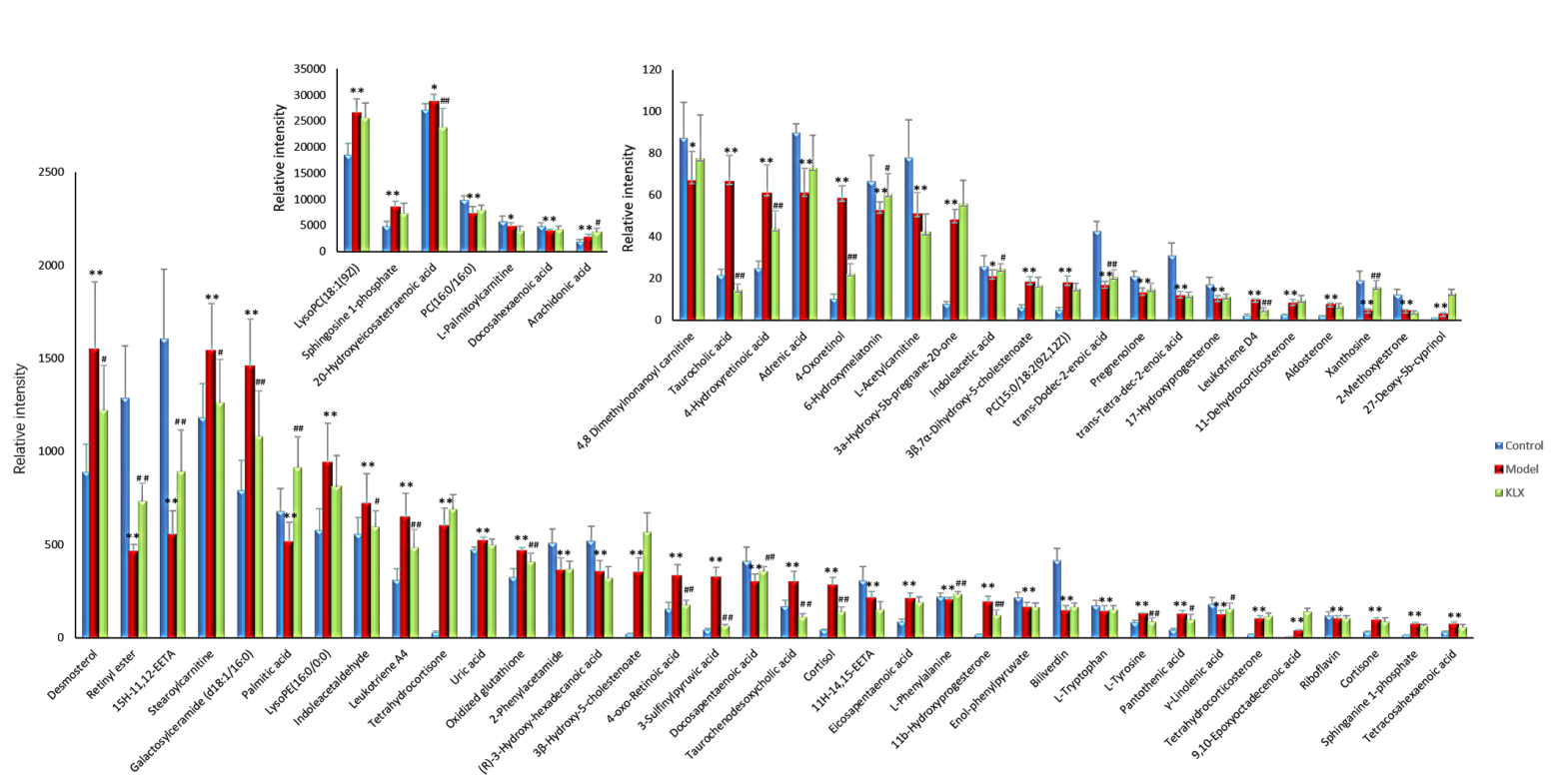
**

**Fig. S6. Content changesin serum biomarkers from mice in the control, model and KLX-treated groups.**

Values are presented as mean ±SEM. (n=10 per group). Data were analyzed by Student’s *t*test. Model group data were compared with control group data; *P < 0.05, **P < 0.01. KLX-treated group data were compared with model group data; ^#^P < 0.05, ^# #^P < 0.01.

**
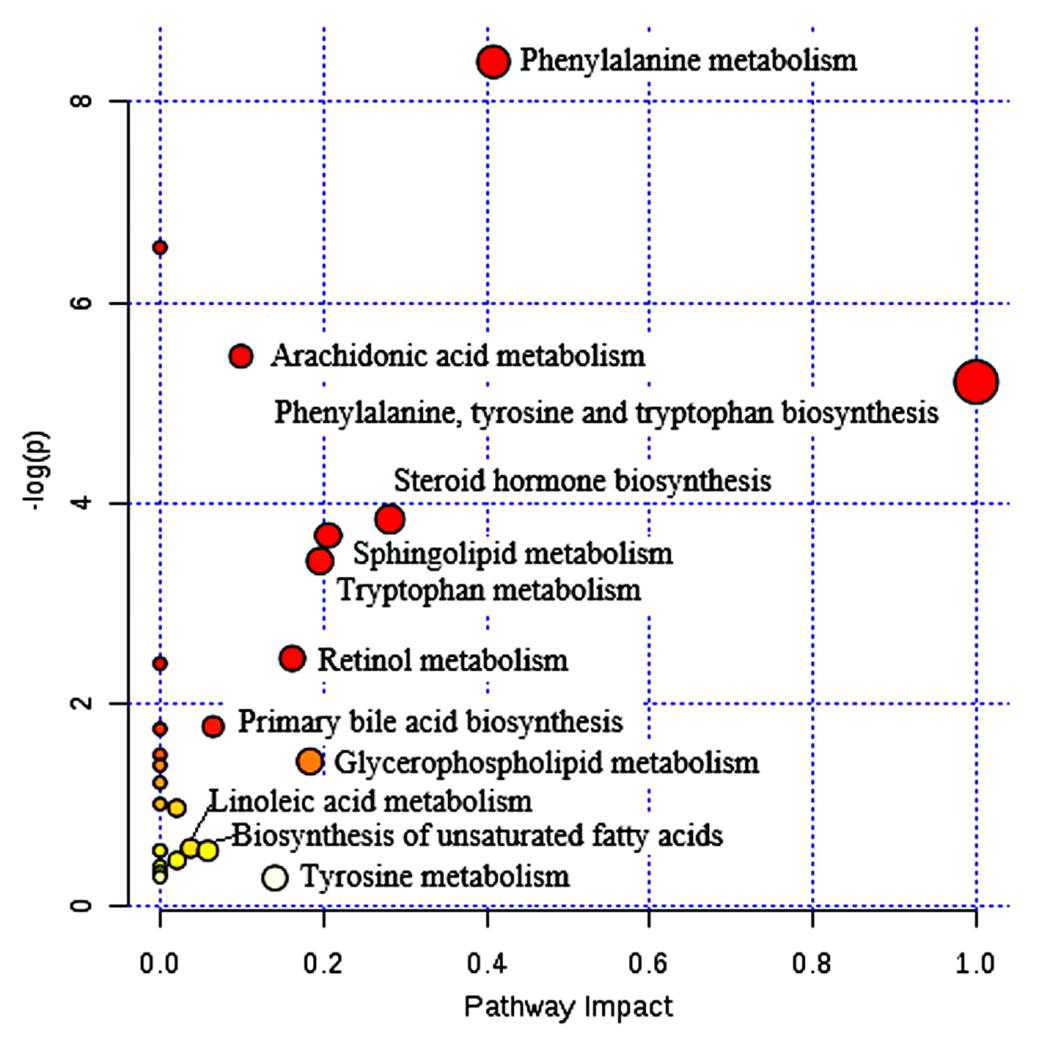
**

**Fig. S7. Key metabolic pathways of potential DR biomarkers in mouse serum after oral administration of KLX based on MetPA analysis.**

Core pathways were selected based on*P*< 0.05 and impact > 0. Phenylalanine-tyrosine-tryptophan biosynthesis (*P* = 0.01; impact = 1.00); phenylalanine metabolism (*P* = 0.0002; impact = 0.41); steroid hormone biosynthesis (*P* = 0.02; impact = 0.28); sphingolipid metabolism (*P* = 0.03; impact = 0.21); tryptophan metabolism (*P* = 0.03; impact = 0.20); arachidonic acid metabolism (*P* = 0.001; impact = 0.10).

**Table S1. Potential blood biomarkers of DR model mice based on UPLC-Q/TOF-MS analysis.**

| **NO.** | **Rt/min** | **m/z** | **Adduct** | **Fomula** | **Error/ppm** | **Compound** | **Mass Fragments** | **Trend** | **Structure** |
| --- | --- | --- | --- | --- | --- | --- | --- | --- | --- |
| 1 | 0.95 | 169.0351 | M+H | C_5_H_4_N_4_O_3_ | -3.37 | Uric acid | 169.0368[C5H4N4O3+H]+(7.53)  153.0154[C5H3N3O3-e]+(-9.51)  127.0365[C4H3N3O2+H+H]+(-9.51)  124.0147[C4H3N3O2-e-H]+(4.56)  96.0201[C3H3N3O-e-H]+(8.56) | ↑ | 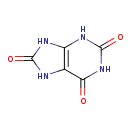 |
| 2 | 1.20 | 173.96 | M+Na | C_3_H_3_O_5_S | 4.67 | 3-Sulfinylpyruvic acid | 156.9574[C_3_H_2_O_4_S+Na]^+^(4.75)  155.9496[C_3_H_2_O_4_S+Na-H]^+^(4.90)  85.9443[O2S+Na-H]+(11.90)  68.9953[CHO2+Na+H]+(8.73) | ↑ | 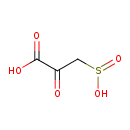 |
| 3 | 1.21 | 136.0751 | M+H | C_8_H_9_NO | -4.41 | 2-Phenylacetamide | 119.0481[C8H7O-e]+(-8.74)  91.0555[C7H7-e]^+^(13.92)  55.0190[C3HO+H+H]+(20.93) | ↓ | 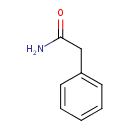 |
| 4 | 1.21 | 165.054 | M+H | C_9_H_8_O_3_ | -3.75 | Enol-phenylpyruvate | 103.0540[C8H5+H+H]+(-2.30)  92.0615[C_7_H_6_+H+H]^+^(-5.92)  91.0554[C_7_H_6_+H]^+^(12.22)  78.0467[C6H5+H]+(4.73) | ↓ | 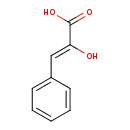 |
| 5 | 1.21 | 182.0805 | M+H | C_9_H_11_NO_3_ | -3.77 | L-Tyrosine | 147.0446[C9H7O2-e]+(3.43)  119.0478[C8H7O-e]+(-11.17)  107.0505[C7H7O-e]+(12.66)  91.0539[C_7_H_6_+H]^+^(-3.61) | ↑ | 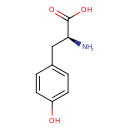 |
| 6 | 1.21 | 613.1605 | M+H | C_20_H_32_N_6_O_12_S_2_ | 2.01 | Oxidized glutathione | 613.1561[C20H32N6O12S2+H]+(-5.23)  595.1279[C20H29N5O12S2-e]^+^(5.07)  484.1178[C15H24N5O9S2+H+H]+(2.38)  231.0427[C8H12N2O4S-e-H]+(-3.28)  130.0496[C5H8NO3-e]+(-2.00) | ↑ | 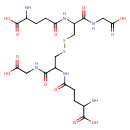 |
| 7 | 1.42 | 307.0659 | M+Na | C_10_H_12_N_4_O_6_ | 3.38 | Xanthosine | 307.0663[C10H12N4O6+Na]+(4.34)  178.0349[C6H8N2O3+Na-H]^+^(-0.39)  177.0294[C8H6N2O3-e-H]+(-0.44)  175.0250[C7H4N4O2-e-H]+(-0.24) | ↓ | 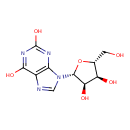 |
| 8 | 1.44 | 160.0752 | M+H | C_10_H_9_NO | -3.33 | Indoleacetaldehyde | 158.0587[C10H9NO-e-H]+(-8.74)  133.0637[C9H7O+H+H]^+^(-7.82)  117.0558[C8H6N+H]+(-12.54)  116.0485[C8H6N-e]+(-7.80)  77.0392[C6H4+H]+(7.60) | ↑ | 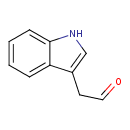 |
| 9 | 1.45 | 176.0698 | M+H | C_10_H_9_NO_2_ | -4.77 | Indoleacetic acid | 158.0594[C10H8NO-e]+(-3.82)  129.0569[C9H7N-e]^+^(-2.49)  118.0667[C8H6N+H+H]+(13.78) | ↓ | 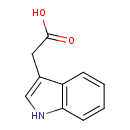 |
| 10 | 1.62 | 166.0855 | M+H | C_9_H_11_NO_2_ | -4.39 | L-Phenylalanine | 120.0809[C8H10N-e]+(1.32)  118.0638[C8H9N-e-H]^+^(-11.86)  93.0697[C7H7+H+H]+(-1.46)  79.0537[C6H6+H]+(-6.77) | ↓ | 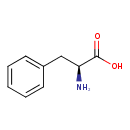 |
| 11 | 1.72 | 220.1176 | M+H | C_9_H_17_NO_5_ | -1.60 | Pantothenic acid | 242.1019[C9H17NO5+Na]+(8.11)  153.0511[C6H11O3+Na-H]^+^(-7.31)  142.0877[C7H12NO2-e]+(10.25)  103.0742[C5H11O2-e]+(-10.77) | ↑ | 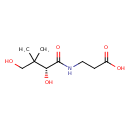 |
| 12 | 2.02 | 205.0964 | M+H | C_11_H_12_N_2_O_2_ | -3.46 | L-Tryptophan | 159.0915[C10H11N2-e]+(-1.20)  144.0824[C10H8N+H+H]^+^(11.14)  132.0808[C9H8N+H+H]+(-5.17)  118.0651[C8H7N+H]+(-0.05)  77.0392[C6H4+H]+(8.59) | ↓ | 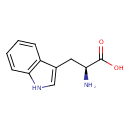 |
| 13 | 2.28 | 293.1145 | M+FA-H | C_13_H_16_N_2_O_3_ | 0.63 | 6-Hydroxymelatonin | 293.1140[C_13_H_16_N_2_O_3_+FA-H]^-^(-1.19)  164.0363[C_7_H_5_NO+FA-H]^-^(6.08)  121.0295[C_7_H_6_O_2_+e-H]^-^(-0.06) | ↓ | 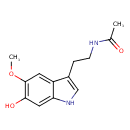 |
| 14 | 2.49 | 377.1459 | M+H | C_17_H_20_N_4_O_6_ | 0.86 | Riboflavin | 377.1443[C17H20N4O6+H]+(-3.34)  359.1358[C17H19N4O5-e]^+^(2.37) | ↓ | 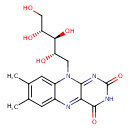 |
| 15 | 3.02 | 202.1093 | M-H | C_9_H_17_NO_4_ | 4.12 | L-Acetylcarnitine | 202.1074[C_9_H_17_NO_4_-H]^-^(-5.53)  158.1173[C_8_H_17_NO_2_-H]^-^(-8.81) | ↓ | 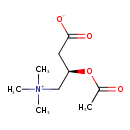 |
| 16 | 3.72 | 538.2812 | M+Na | C_26_H_45_NO_7_S | 0.61 | Taurocholic acid | 538.2828[C26H45NO7S+Na]+(3.45)  520.2700[C26H43NO6S+Na]^+^(-0.58)  502.2616[C26H42NO5S+Na-H]+(3.80)  413.2634[C24H39O4+Na-H]+(-6.76) | ↑ | 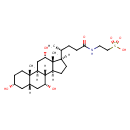 |
| 17 | 3.93 | 541.2599 | M+FA-H | C_25_H_40_N_2_O_6_S | 1.96 | Leukotriene D4 | 495.2548[C_25_H_38_N_2_O_4_S+FA-H]^-^(-2.80)  365.2336[C_20_H_31_O_3_+FA-H+H]^-^(0.85)  113.0234[C_4_H_5_O+FA-H-H]^-^(-8.81) | ↑ | 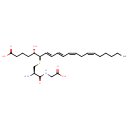 |
| 18 | 3.97 | 383.2042 | M+Na | C_21_H_28_O_5_ | -2.43 | Aldosterone | 383.1844[C21H28O5+Na]+(3.83)  343.1895[C21H27O4-e]^+^(-2.62) | ↑ | 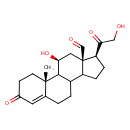 |
| 19 | 4.10 | 361.2005 | M-H | C_21_H_30_O5 | -4.24 | Cortisol | 361.2032[C_21_H_30_O_5_-H]^-^(3.30)  343.1892[C_21_H_28_O_4_-H]^-^(-6.74)  271.1694[C_18_H_24_O_2_-H]^-^(-3.39)  241.1606[C_17_H_22_O-H]^-^(3.28)  161.0977[C_11_H_14_O-H]^-^(2.69) | ↑ | 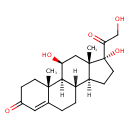 |
| 20 | 4.27 | 359.1851 | M-H | C_21_H_28_O_5_ | -3.55 | Cortisone | 359.1884[C_21_H_28_O_5_-H]^-^(5.63)  313.1794[C_20_H_26_O_3_-H]^-^(-4.92)  256.1471[C_17_H_20_O_2_+e]^-^(1.08)  189.0924[C_12_H_14_O_2_-H]^-^(1.66)  169.0864[C_9_H_14_O_3_+e-H]^-^(-3.68) | ↑ | 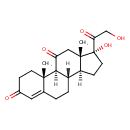 |
| 21 | 4.39 | 363.2165 | M-H | C_21_H_32_O_5_ | -3.34 | Tetrahydrocortisone | 315.1982[C_20_H_27_O_3_+e]^-^(5.18)  273.1858[C_18_H_26_O_2_-H]^-^(-0.85)  243.1743[C_17_H_24_O-H]^-^(-4.55)  193.1243[C_12_H_18_O_2_-H]^-^(4.29)  151.0761[C_9_H_12_O_2_-H]^-^(-1.97) | ↑ | 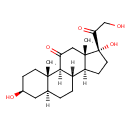 |
| 22 | 4.50 | 333.2061 | M+H | C_20_H_28_O_4_ | 0.30 | 11β-Hydroxyprogesterone | 269.1900[C19H25O-e]+(-0.04)  175.1104[C12H14O+H]^+^(-7.96)  161.0972[C11H14O-e-H]+(7.40) | ↑ | 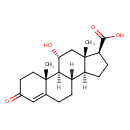 |
| 23 | 4.57 | 301.217 | M+H | C_20_H_28_O_2_ | 2.59 | 4-Oxoretinol | 301.2166[C20H28O2+H]+(1.45)  197.1336[C15H16+H]+(5.50)  147.1177[C11H13+H+H]+(5.33)  133.0999[C10H12+H]+(-9.71)  93.0698[C7H8+H]+(-0.89) | ↑ | 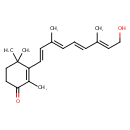 |
| 24 | 4.71 | 500.3032 | M+H | C_26_H_45_NO_6_S | -1.57 | Taurochenodesoxycholic acid | 522.2831[C_26_H_45_NO_6_S+Na]+(-5.63)  486.2671[C_26_H_42_NO_4_S+Na-H]^+^(4.67)  354.1747[C_18_H_29_NO_4_S-e-H]+(3.76) | ↑ | 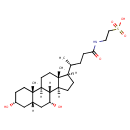 |
| 25 | 4.84 | 583.255 | M+H | C_33_H_34_N_4_O_6_ | -0.16 | Biliverdin | 605.2390[C33H34N4O6+Na]+(3.29)  583.2551[C33H34N4O6+H]^+^(-0.02)  568.2410[C33H33N3O6+H]+(-5.54)  524.2385[C31H31N4O4+H]+(-6.32)  510.2228[C30H29N4O4+H]+(-6.60) | ↓ | 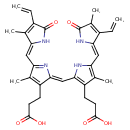 |
| 26 | 4.85 | 243.1603 | M+FA-H | C_12_H_22_O_2_ | 0.39 | trans-Dodec-2-enoic acid | 243.1590[C_12_H_22_O_2_+FA-H]^-^(-4.96)  225.1492[C_12_H_20_O+FA-H]^-^(-1.58)  181.1589[C_12_H_21_O+e]^-^(-4.57) | ↓ | 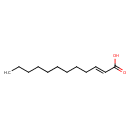 |
| 27 | 4.89 | 335.2211 | M-H | C_20_H_32_O_4_ | -4.99 | 15H-11,12-EETA | 318.2212[C_20_H_31_O_3_-H]^-^(3.57)  208.1114[C_12_H_17_O_3_-H]^-^(4.31)  179.1060[C_11_H_16_O_2_-H]^-^(-9.78)  155.1080[C_9_H_16_O_2_-H]^-^(1.69)  107.0873[C_8_H_13_-H-H]^-^(6.56) | ↓ | 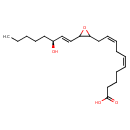 |
| 28 | 4.89 | 343.1902 | M-H | C_21_H_28_O_4_ | -3.63 | 11-Dehydrocorticosterone | 343.1889[C_21_H_28_O_4_-H]^-^(-1.67)  286.1555[C_18_H_24_O_3_-H-H]^-^(-6.77)  283.1688[C_19_H_24_O_2_-H]^-^(-5.51)  273.1860[C_18_H_24_O_2_+e+H]^-^(-0.05)  121.0671[C_8_H_10_O-H]^-^(9.97) | ↑ | 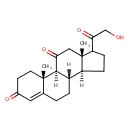 |
| 29 | 5.11 | 349.2372 | M-H | C_21_H_34_O_4_ | -3.47 | Tetrahydrocorticosterone | 349.2357[C_21_H_34_O_4_-H]^-^(-7.75)  288.2115[C_19_H_28_O_2_+e]^-^(7.28)  269.1910[C_19_H_26_O-H]^-^(-0.31) | ↑ | 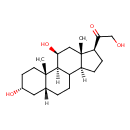 |
| 30 | 5.17 | 315.1956 | M+H | C_20_H_26_O_3_ | 0.29 | 4-oxo-Retinoic acid | 270.1629[C18H21O2+H]+(5.39)  259.1683[C17H22O2+H]^+^(-3.75)  254.1667[C18H22O-e]+(0.53)  198.1392[C15H16+H+H]+(-5.25)  171.1168[C13H14+H]+(-0.41) | ↑ | 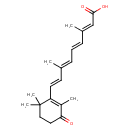 |
| 31 | 5.40 | 330.264 | M+H | C_18_H_35_NO_4_ | 0.47 | 4,8 Dimethylnonanoyl carnitine | 330.2642[C18H35NO4+H]+(0.95)  71.0863[C5H11-e]^+^(10.58) | ↓ | 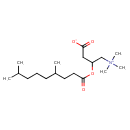 |
| 32 | 5.45 | 433.3313 | M+H | C_27_H_44_O_4_ | 0.06 | 3β,7α-Dihydroxy-5-cholestenoate | 398.3176[C27H41O2+H]+(-1.03)  369.3177[C26H41O-e]^+^(7.03) | ↑ | 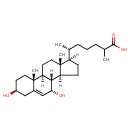 |
| 33 | 5.52 | 301.1809 | M+H | C_19_H_24_O_3_ | 3.45 | 2-Methoxyestrone | 255.1725[C18H22O+H]+(-7.17)  131.0856[C10H11-e]^+^(0.50)  119.0851[C9H9+H+H]+(-3.69) | ↓ | 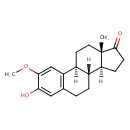 |
| 34 | 5.61 | 317.2473 | M+H | C_21_H_32_O_2_ | -0.72 | Pregnenolone | 159.1155[C12H16-e-H]+(-8.24)  123.0800[C8H12O-e-H]^+^(-3.71)  93.0601[C7H9-e]+(-7.75) | ↓ | 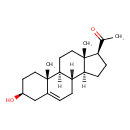 |
| 35 | 5.61 | 459.3447 | M+Na | C_27_H_48_O_4_ | 0.40 | 27-Deoxy-5β-cyprinol | 219.1719[C13H23O+Na+H]+(-0.14)  105.0673[C6H11+Na-H]^+^(-2.04) | ↑ | 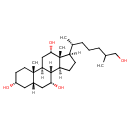 |
| 36 | 5.69 | 271.1912 | M+FA-H | C_14_H_26_O_2_ | -1.22 | trans-Tetra-dec-2-enoic acid | 253.1829[C_14_H_24_O+FA-H]^-^(7.98)  209.1892[C_14_H_25_O+e]^-^(-8.94) | ↓ | 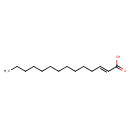 |
| 37 | 6.01 | 378.2399 | M-H | C_18_H_38_NO_5_P | -4.20 | Sphingosine 1-phosphate | 378.2396[C_18_H_38_NO_5_P-H]^-^(-5.10)  362.2482[C_18_H_37_NO_4_P+e]^-^(4.64)  96.9692[H_2_O_4_P+e]^-^(-4.70) | ↑ | 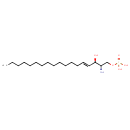 |
| 38 | 6.01 | 380.2566 | M+H | C_18_H_38_NO_5_P | 1.50 | Sphingosine 1-phosphate | 402.2362[C18H38NO5P+Na]+(-4.51)  380.2551[C18H38NO5P+H]^+^(-2.58)  284.2946[C18H36NO+H+H]+(-0.45)  266.2834[C18H34N+H+H]+(-3.11)  264.2695[C18H34N-e]+(3.58) | ↑ | 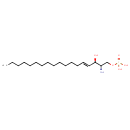 |
| 39 | 6.16 | 335.2214 | M-H | C_20_H_32_O_4_ | -4.07 | 11H-14,15-EETA | 335.2208[C_20_H_32_O_4_-H]^-^(-6.05)  195.1014[C_11_H_17_O_3_-H-H]^-^(-6.46)  181.1240[C_11_H_19_O_2_-H-H]^-^(2.97)  177.0921[C_11_H_15_O_2_-H-H]^-^(0.05)  165.0933[C_10_H_15_O_2_-H-H]^-^(7.36) | ↓ | 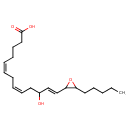 |
| 40 | 6.28 | 404.2553 | M+Na | C_18_H_40_NO_5_P | 4.40 | Sphinganine 1-phosphate | 382.2706[C18H40NO5P+H]+(-2.74)  284.2930[C18H38NO-e]+(-6.16)  285.3011[C18H38NO+H]+(-5.33)  120.9648[H2O4P+Na+H]+(-11.12) | ↑ | 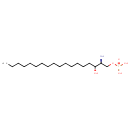 |
| 41 | 6.39 | 317.2117 | M+H | C_20_H_28_O_3_ | 1.78 | 4-Hydroxyretinoic acid | 317.2131[C20H28O3+H]+(6.30)  271.2042[C19H27O-e]^+^(-5.38)  173.1335[C13H16+H]+(5.64)  161.1311[C12H15+H+H]+(-8.52)  133.1023[C10H12+H]+(8.17) | ↑ | 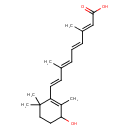 |
| 42 | 7.02 | 454.2929 | M+H | C_21_H_44_NO_7_P | 0.28 | LysoPE(16:0/0:0) | 454.2945[C21H44NO7P+H]+(3.85)  314.2839[C19H37O3+H]^+^(7.51)  313.2733[C19H37O3-e]+(-1.49)  113.1319[C8H17-e]+(-5.24) | ↑ | 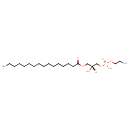 |
| 43 | 7.22 | 400.343 | M+H | C_23_H_45_NO_4_ | 2.15 | L-Palmitoylcarnitine | 257.2464[C16H31O2+H+H]+(-4.19)  239.2363[C16H31O-e]+(-2.92)  71.0860[C5H11-e]+(6.29)  60.0807[C3H9N+H]+(-0.77) | ↓ | 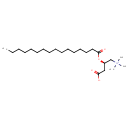 |
| 44 | 7.38 | 319.2269 | M-H | C_20_H_32_O_3_ | -2.99 | 20-Hydroxyeicosatetraenoic acid | 319.2257[C_20_H_32_O_3_-H]^-^(-6.53)  302.2236[C_20_H_31_O_2_-H]^-^(-5.13)  229.1949[C_17_H_25_+e]^-^(-5.31)  215.1800[C_16_H_23_+e]^-^(-2.51)  179.1069[C_11_H_16_O_2_-H]^-^(-4.41) | ↑ | 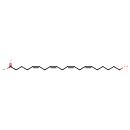 |
| 45 | 7.39 | 522.3568 | M+H | C_26_H_52_NO_7_P | 2.65 | LysoPC(18:1(9Z)) | 544.3383[C26H52NO7P+Na]+(1.83)  527.3311[C26H51NO6P+Na]^+^(-6.65)  485.2634[C23H43O7P+Na]+(-0.81)  361.2715[C21H39O3+Na-H]+(0.37)  184.0725[C5H13NO4P+H+H]+(-4.16)) | ↑ | 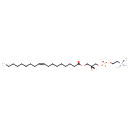 |
| 46 | 7.56 | 317.2109 | M-H | C_20_H_30_O_3_ | -4.17 | Leukotriene A4 | 301.2167[C_20_H_29_O_2_+e]^-^(-2.14)  231.1752[C_16_H_23_O+e]^-^(-0.94)  203.1805[C_15_H_22_+e+H]^-^(-0.48)  187.0783[C_12_H_13_O_2_-H-H]^-^(10.07)  135.1182[C_10_H_17_-H-H]^-^(2.34) | ↑ | 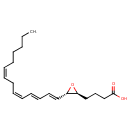 |
| 47 | 7.93 | 295.2276 | M-H | C_18_H_32_O_3_ | -1.06 | 9,10-Epoxyoctadecenoic acid | 277.2176[C_18_H_30_O_2_-H]^-^(0.96)  182.1687[C_12_H_21_O+e+H]^-^(5.78)  171.1026[C_9_H_16_O_3_-H]^-^(-0.23)  137.0970[C_9_H_14_O-H]^-^(-1.41) | ↑ | 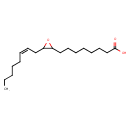 |
| 48 | 7.98 | 417.3375 | M+H | C_27_H_44_O_3_ | 2.83 | 3β-Hydroxy-5-cholestenoate | 417.3370[C27H44O3+H]+(1.82)  381.3174[C27H41O-e]^+^(5.77)  273.2211[C19H29O-e]+(-0.67)  175.1497[C13H18+H]+(8.81)  105.0698[C8H10-e-H]+(-0.62) | ↑ | 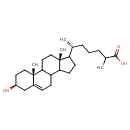 |
| 49 | 8.14 | 428.375 | M+H | C_25_H_49_NO_4_ | 3.57 | Stearoylcarnitine | 428.3748[C25H49NO4+H]+(2.99)  267.2684[C18H35O-e]^+^(0.45) | ↑ | 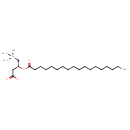 |
| 50 | 8.21 | 331.2253 | M+H | C_21_H_30_O_3_ | -4.55 | 17-Hydroxyprogesterone | 107.0849[C8H11-e]+(-5.80)  93.0690[C7H9-e]+(-8.65) | ↓ | 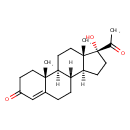 |
| 51 | 8.90 | 301.2165 | M-H | C_20_H_30_O_2_ | -2.66 | Retinyl ester | 257.2281[C_19_H_29_+e]^-^(2.72)  217.1234[C_14_H_18_O_2_-H]^-^(-3.63)  215.1793[C_16_H_25_-H-H]^-^(-5.73) | ↓ | 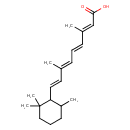 |
| 52 | 9.12 | 303.2328 | M+H | C_20_H_30_O_2_ | 3.23 | Eicosapentaenoic acid | 201.1643[C15H22-e-H]+(2.61)  175.1486[C13H19-e]+(2.33)  137.1324[C10H15+H+H]+(-0.55)  95.0856[C7H11-e]+(0.05) | ↑ | 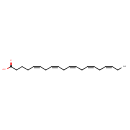 |
| 53 | 9.12 | 277.2174 | M-H | C_18_H_30_O_2_ | 0.46 | γ-Linolenic acid | 233.2285[C_17_H_29_+e]^-^(4.58)  147.0823[C_10_H_13_O-H-H]^-^(5.01) | ↓ | 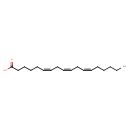 |
| 54 | 9.15 | 271.2277 | M-H | C_16_H_32_O_3_ | -0.63 | (R)-3-Hydroxy-hexadecanoic acid | 225.2206[C_15_H_30_O-H]^-^(-8.05)  223.1711[C_14_H_25_O_2_-H-H]^-^(3.67)  84.0205[C_4_H_5_O_2_-H]^-^(-14.65) | ↓ | 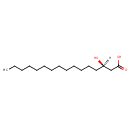 |
| 55 | 9.36 | 329.2485 | M+H | C_22_H_32_O_2_ | 3.17 | Docosahexaenoic acid | 657.4855[C22H32O2+H+M]+(-3.40)  329.2487[C22H32O2+H]+(3.58)  201.1638[C15H22-e-H]+(0.29)  163.1489[C12H18+H]+(4.32)  149.1324[C11H17-e]+(-0.46) | ↓ | 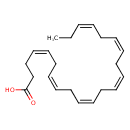 |
| 56 | 9.58 | 303.2325 | M-H | C_20_H_32_O_2_ | -1.52 | Arachidonic acid | 303.2327[C_20_H_32_O_2_-H]^-^(-0.85)  285.2210[C_20_H_30_O-H]^-^(-4.96)  229.1972[C_17_H_27_-H-H]^-^(4.21)  219.1377[C_14_H_20_O_2_-H]^-^(-5.92)  163.1490[C_12_H_20_-H]^-^(-1.26) | ↑ | 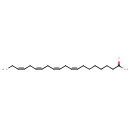 |
| 57 | 9.71 | 329.248 | M-H | C_22_H_34_O_2_ | -1.89 | Docosapentaenoic acid | 313.2162[C_21_H_31_O_2_-H-H]^-^(-3.73)  285.2601[C_21_H_33_+e]^-^(4.85)  259.1716[C_17_H_25_O_2_-H-H]^-^(4.81)  149.1338[C_11_H_17_+e]^-^(1.92)  121.1030[C_9_H_14_-H]^-^(5.69) | ↓ | 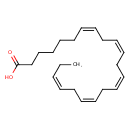 |
| 58 | 10.06 | 357.2799 | M+H | C_24_H_36_O_2_ | 3.12 | Tetracosahexaenoic acid | 357.2787[C24H36O2+H]+(-0.17)  175.1490[C13H19-e]+(4.60)  163.1467[C12H18+H]+(-9.16)  135.1175[C10H15-e]+(4.52) | ↑ | 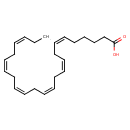 |
| 59 | 10.21 | 744.5514 | M+H | C_41_H_78_NO_8_P | -3.15 | PC(15:0/18:2(9Z,12Z)) | 744.5494[C41H78NO8P+H]+(-5.91)  184.0716[C5H13NO4P+H+H]+(-9.14) | ↑ | 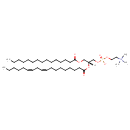 |
| 60 | 10.33 | 333.2796 | M+H | C_22_H_36_O_2_ | 2.39 | Adrenic acid | 333.2802[C22H36O2+H]+(4.31)  163.1480[C12H20-e-H]+(-1.05) | ↓ | 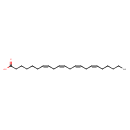 |
| 61 | 10.39 | 255.2334 | M-H | C_16_H_32_O_2_ | 1.61 | Palmitic acid | 255.2342[C_16_H_32_O_2_-H]^-^(4.85)  236.2137[C_16_H_30_O_20_-H-H]^-^(-3.42) | ↓ | 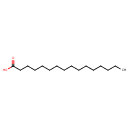 |
| 62 | 10.83 | 319.2642 | M+H | C_21_H_34_O_2_ | 3.38 | 3α-Hydroxy-5β-pregnane-20-one | 287.2371[C20H30O+H]+(0.51)  177.1620[C13H20+H]+(-10.27)  161.1329[C12H18-e-H]+(2.81)  147.1171[C11H16-e-H]+(1.56) | ↑ | 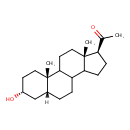 |
| 63 | 13.14 | 722.5563 | M+Na | C_40_H_77_NO_8_ | 3.43 | Galactosylceramide (d18:1/16:0) | 700.5703[C40H77NO8+H]+(-2.66)  683.5699[C40H76NO7+H]+(0.69)  560.4990[C34H66NO3+Na+H]+(-4.21)  521.5182[C34H66NO2+H]+(3.02)  490.4949[C33H62NO+H+H]+(-6.81) | ↑ | 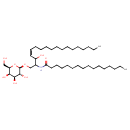 |
| 64 | 13.58 | 385.3481 | M+H | C_27_H_44_O | 4.12 | Desmosterol | 385.3488[C27H44O+H]+(6.15)  353.3193[C26H40+H]+(-2.75)  161.1330[C12H16+H]+(3.41)  147.1175[C11H14+H]+(4.50) | ↑ | 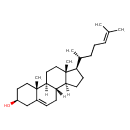 |
| 65 | 16.47 | 734.571 | M+H | C_40_H_80_NO_8_P | 2.13 | PC(16:0/16:0) | 552.5120[C35H67O4+H]+(1.53)  479.3344[C24H49NO6P+H]+(-5.29)  184.0748[C5H13NO4P+H+H]+(8.52) | ↓ | 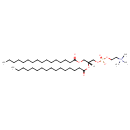 |

↑: Increased blood biomarker content; ↓: decreased blood biomarker content.

**Table S2. Critical metabolic pathways of potential DR blood biomarkers after oral administration of KLX based on MetPA analysis.**

| **NO.** | **pathway name** | **Total** | **Hits** | **Raw p** | **-log(p)** | **FDR** | **Impact** |
| --- | --- | --- | --- | --- | --- | --- | --- |
| 1 | Phenylalanine, tyrosine and tryptophan biosynthesis | 4 | 2 | 0.01 | 5.21 | 0.11 | 1.00 |
| 2 | Phenylalanine metabolism | 11 | 4 | 0.0002 | 8.39 | 0.02 | 0.41 |
| 3 | Steroid hormone biosynthesis | 72 | 6 | 0.02 | 3.86 | 0.34 | 0.28 |
| 4 | Sphingolipid metabolism | 21 | 3 | 0.03 | 3.68 | 0.34 | 0.21 |
| 5 | Tryptophan metabolism | 40 | 4 | 0.03 | 3.42 | 0.38 | 0.20 |
| 6 | Glycerophospholipid metabolism | 30 | 2 | 0.24 | 1.43 | 1.00 | 0.18 |
| 7 | Retinol metabolism | 16 | 2 | 0.09 | 2.46 | 0.82 | 0.16 |
| 8 | Tyrosine metabolism | 44 | 1 | 0.76 | 0.28 | 1.00 | 0.14 |
| 9 | Arachidonic acid metabolism | 36 | 5 | 0.001 | 5.47 | 0.11 | 0.10 |
| 10 | Primary bile acid biosynthesis | 46 | 3 | 0.17 | 1.78 | 1.00 | 0.06 |
| 11 | Porphyrin and chlorophyll metabolism | 27 | 1 | 0.58 | 0.55 | 1.00 | 0.06 |
| 12 | Glutathione metabolism | 26 | 1 | 0.56 | 0.57 | 1.00 | 0.04 |
| 13 | Purine metabolism | 68 | 2 | 0.63 | 0.45 | 1.00 | 0.02 |
| 14 | Pantothenate and CoA biosynthesis | 15 | 1 | 0.38 | 0.97 | 1.00 | 0.02 |
| 15 | Biosynthesis of unsaturated fatty acids | 42 | 6 | 0 | 6.55 | 0.06 | 0 |
| 16 | Ubiquinone and other terpenoid-quinone biosynthesis | 3 | 1 | 0.09 | 2.40 | 0.82 | 0 |
| 17 | Linoleic acid metabolism | 6 | 1 | 0.17 | 1.76 | 1.00 | 0 |
| 18 | Taurine and hypotaurine metabolism | 8 | 1 | 0.22 | 1.50 | 1.00 | 0 |
| 19 | alpha-Linolenic acid metabolism | 9 | 1 | 0.25 | 1.40 | 1.00 | 0 |
| 20 | Riboflavin metabolism | 11 | 1 | 0.29 | 1.22 | 1.00 | 0 |
| 21 | Aminoacyl-tRNA biosynthesis | 69 | 3 | 0.36 | 1.01 | 1.00 | 0 |
| 22 | Fatty acid elongation in mitochondria | 27 | 1 | 0.58 | 0.55 | 1.00 | 0 |
| 23 | Cysteine and methionine metabolism | 27 | 1 | 0.58 | 0.55 | 1.00 | 0 |
| 24 | Steroid biosynthesis | 35 | 1 | 0.67 | 0.40 | 1.00 | 0 |
| 25 | Fatty acid metabolism | 39 | 1 | 0.71 | 0.34 | 1.00 | 0 |
| 26 | Fatty acid biosynthesis | 43 | 1 | 0.75 | 0.29 | 1.00 | 0 |

**Table S3. Components of KLX detected by UPLC-Q/TOF-MS in positive-ion mode.**

| **NO.** | **Rt / min** | **m/z** | **Adducts** | **Fomula** | **Error / ppm** | **Compouds Name** | **Mass Fragments** | **Source** |
| --- | --- | --- | --- | --- | --- | --- | --- | --- |
| 1 | 0.42 | 198.1229 | M+H | C_9_H_15_N_3_O_2_ | -4.19 | L-Histidine trimethylbetaine | 198.1230[C_9_H_15_N_3_O_2_+H]+(-3.56)  154.1354[C_8_H_15_N_3_+H]+(10.03)  139.0493 [C_6_H_6_N_2_O_2_+H]+(-6.29) | F |
| 2 | 0.44 | 499.2252 | M+H | C_18_H_34_N_4_O_12_ | 1.28 | N2-Maltulosylarginine | 481.2149[C_18_H_33_N_4_O_11_-e]+(1.77)  419.2163[C_17_H_29_N_4_O_8_+H+H]+(6.28)  175.1183[C_6_H_13_N_4_O_2_+H+H]+(-4.03) | B, D, E |
| 3 | 0.44 | 342.1391 | M+H | C_12_H_23_NO_10_ | -1.15 | Lactosamine | 342.1448[C_12_H_23_NO_10_+H]+(15.58)  306.1136[C_12_H_20_NO_8_-e]+(-15.57)  240.0865[C_8_H_15_O_8_+H]+(10.39)  144.0660[C_6_H_10_NO_3_-e]+(3.39) | D, E |
| 4 | 0.44 | 324.1290 | M+Na | C_16_H_19_N_3_O_3_ | -9.58 | Tryptophyl-Proline | 324.1284[C_16_H_19_N_3_O_3_+Na]+(-10.59)  308.1131[C_16_H_17_N_2_O_3_+Na]+(4.09)  221.0886[C_9_H_13_N_2_O_3_+Na+H]+(-4.65)  127.0393[C_7_H_6_N+Na]+(0.92) | D, E |
| 5 | 0.44 | 295.1132 | 2M+H | C_5_H_9_NO_4_ | -1.23 | L-Glutamic acid | 277.1036[C_5_H_8_NO_3_-e+M]+(2.05)  259.0899[C_5_H_6_NO_2_-e+M]+(-10.17)  231.0960[C_4_H_6_NO-e+M]+(-6.56)  133.0737[C_5_H_9_NO_3_+H+H]+(2.20) | C, D |
| 6 | 0.45 | 248.1125 | M+H | C_10_H_17_NO_6_ | -1.59 | Linamarin | 248.1127[C_10_H_17_NO_6_+H]+(-0.64)  128.0683[C_6_H_9_NO_2_+H]+(-17.43)  98.0606[C_5_H_7_NO+H]+(5.65)  70.0662[C_4_H_6_N+H+H]+(15.82) | A, B, E |
| 7 | 0.45 | 118.0856 | M+H | C_5_H_11_NO_2_ | -5.55 | Betaine | 102.0557[C_4_H_8_NO_2_-e]+(7.13)  72.0786[C_4_H_10_N-e]+(-29.35) | D |
| 8 | 0.46 | 278.1233 | M+H | C_11_H_19_NO_7_ | -0.54 | N-(1-Deoxy-1-fructosyl)proline | 278.1270[C_11_H_19_NO_7_+H]+(12.50)  196.0961[C_10_H_13_NO_3_+H]+(-3.53) | D, E |
| 9 | 0.50 | 527.1592 | M+Na | C_18_H_32_O_16_ | 1.82 | alpha-D-Glucopyranosyl-(1->6)-alpha-D-glucopyranosyl-(1->2)-D-glucose | 527.1580[C_18_H_32_O_16_+Na]+(-0.38)  437.1207[C_15_H_26_O_13_+Na]+(-13.39)  365.1118[C_14_H_21_O_11_-e]+(10.78)  203.0570[C_8_H_11_O_6_-e]+(9.71) | A, B, D |
| 10 | 0.70 | 280.1388 | M+H | C_11_H_21_NO_7_ | -1.00 | N-(1-Deoxy-1-fructosyl)valine | 262.1293[C_11_H_20_NO_6_-e]+(2.87)  216.1255[C_10_H_18_NO_4_-e]+(11.49)  130.0853[C_6_H_12_NO_2_-e]+(-6.99)  112.0396[C_5_H_7_NO_2_-e-H]+(2.32) | D, E |
| 11 | 0.76 | 152.0698 | M+H | C_8_H_9_NO_2_ | -5.58 | 2-Phenylglycine | 137.0469[C_7_H_8_NO_2_-e]+(-2.13)  134.0611[C_8_H_8_NO-e]+(8.13)  110.0363[C_6_H_4_O_2_+H+H]+(7.36)  107.0736[C_7_H_8_N+H]+(6.26) | C |
| 12 | 0.96 | 344.1346 | M+H | C_15_H_21_NO_8_ | 1.77 | N-(1-Deoxy-1-fructosyl)tyrosine | 344.1334[C_15_H_21_NO_8_+H]+(-1.79)  327.1314[C_15_H_20_NO_7_+H]+(0.40)  262.1064[C_14_H_15_NO_4_+H]+(-3.67) | B, D, E |
| 13 | 1.04 | 238.1070 | M+Na | C_10_H_17_NO_4_ | 9.21 | 7-hydroxyoct-2-enoylglycine | 238.1062[C_10_H_17_NO_4_+Na]+(5.09)  192.0653[C_8_H_12_NO_3_+Na]+(11.15)  106.0759[C_6_H_10_+Na+H]+(5.65) | C |
| 14 | 1.10 | 152.0562 | M+H | C_5_H_5_N_5_O | -2.91 | Guanine | 135.0309[C_5_H_3_N_4_O-e]+(5.88)  110.0340[C_4_H_3_N_3_O+H]+(-7.65)  93.0078[C_4_H_2_N_2_O-e-H]+(-5.55) | A, B, E |
| 15 | 1.13 | 276.1444 | M+H | C_12_H_21_NO_6_ | 0.84 | Glutarylcarnitine | 276.1444[C_12_H_21_NO_6_+H]+(0.84)  259.1372[C_12_H_20_NO_5_+H]+(-16.09)  212.1308 [C_11_H_19_NO_3_-e-H]+(12.41)  69.0333[C_4_H_5_O-e]+(-2.16) | A, B, C, D, E |
| 16 | 1.13 | 294.1548 | M+H | C_12_H_23_NO_7_ | 0.23 | N-(1-Deoxy-1-fructosyl)leucine | 259.1417[C_12_H_20_NO_5_+H]+(13.00)  161.0694[C_6_H_10_NO_4_+H]+(7.15)  132.0991[C_6_H_12_NO_2_+H+H]+(-20.86) | B, C, D, E |
| 17 | 1.22 | 210.1119 | M+Na | C_9_H_17_NO_3_ | 9.62 | N-Heptanoylglycine | 195.0853[C_8_H_14_NO_3_+Na]+(-6.79)  192.1025[C_9_H_15_NO_2_+Na]+(15.32)  164.0695[C_7_H_10_NO_2_+Na+H]+(7.63)  152.1084[C_9_H_15_NO-e-H]+(9.81) | C |
| 18 | 1.28 | 351.1765 | 2M+H | C_7_H_13_NO_4_ | 0.89 | N-Carboxyethyl-g-aminobutyric acid | 351.1759[C_7_H_13_NO_4_+H+M]+(-1.03)  334.1709[C_7_H_12_NO_3_+H+M]+(-7.50)  315.1555[C_7_H_10_NO_2_-e+M]+(1.39)  175.0856[C_7_H_13_NO_4_-e]+(9.25) | D, F |
| 19 | 1.34 | 390.1404 | M+Na | C_14_H_25_NO_10_ | 9.02 | 2-Acetamido-2-deoxy-6-O-a-L-fucopyranosyl-D-glucose | 390.1352[C_14_H_25_NO_10_+Na]+(-4.61)  372.1270[C_14_H_23_NO_9_+Na]+(1.33)  342.1168[C_13_H_21_NO_8_+Na]+(2.63)  246.0960[C_10_H_15_NO_6_-e+H]+(-5.18) | B, D |
| 20 | 1.45 | 328.1397 | M+H | C_15_H_21_NO_7_ | 2.00 | N-(1-Deoxy-1-fructosyl)phenylalanine | 328.1375[C_15_H_21_NO_7_+H]+(-4.67)  311.1322[C_15_H_20_NO_6_+H]+(-13.51)  246.1142[C_14_H_15_NO_3_+H]+(6.99)  120.0800[C_8_H_9_N+H]+(-6.64) | B, E |
| 21 | 1.85 | 367.1507 | M+H | C_17_H_22_N_2_O_7_ | 1.99 | Semilepidinoside B | 367.1504[C_17_H_22_N_2_O_7_+H]+(1.21)  350.1441[C_17_H_21_N_2_O_6_+H]+(-8.97)  276.1255[C_15_H_16_NO_4_+H+H]+(8.85)  188.0665[C_8_H_10_O_5_+H+H]+(-7.09) | A, B, D, E |
| 22 | 2.21 | 291.0862 | M+H | C_15_H_14_O_6_ | -0.40 | cis-3 and trans-2-Hexenyl propionate | 249.0765[C_13_H_12_O_5_+H]+(3.00)  165.0538[C_9_H_8_O_3_+H]+(-4.78)  139.0382[C_7_H_6_O_3_+H]+(-5.75)  123.0435[C_7_H_6_O_2_+H]+(-4.72) | E |
| 23 | 2.32 | 441.2862 | M+Na | C_22_H_42_O_7_ | 9.40 | Palmitoyl glucuronide | 441.2799[C_22_H_42_O_7_+Na]+(-5.47)  153.0127[C_5_H_6_O_4_+Na]+(-20.43)  121.0245[C_5_H_5_O_2_+Na+H]+(-12.96) | F |
| 24 | 2.48 | 433.1128 | M+H | C_21_H_20_O_10_ | -0.30 | Emodin-6-O-beta-D-glucopyranoside | 397.0952[C_21_H_17_O_8_-e]+(8.57)  271.0581[C_15_H_9_O_5_+H+H]+(-7.38)  253.0497[C_15_H_9_O_4_-e]+(0.40) | E |
| 25 | 2.62 | 595.1667 | M+H | C_27_H_30_O_15_ | 1.64 | Apigenin 7-[galactosyl-(1->4)-mannoside] | 595.1584[C_27_H_30_O_15_+H]+(-12.42)  523.1378[C_24_H_25_O_13_+H+H]+(-13.04)  475.1057[C_19_H_24_O_14_-e-H]+(-5.47)  295.0566[C_17_H_10_O_5_+H]+(-11.75) | B, C |
| 26 | 2.66 | 409.1119 | M+Na | C_17_H_22_O_10_ | 3.48 | 1-O-Sinapoylglucose | 409.1074[C_17_H_22_O_10_+Na]+(-7.57)  247.0601[C_11_H_11_O_5_+Na+H]+(9.71)  185.0411[C_6_H_11_O_5_+Na-H]+(-5.10) | E |
| 27 | 2.85 | 579.1509 | M+H | C_30_H_26_O_12_ | 2.12 | ent-Epicatechin-(4alpha->6)-ent-epicatechin | 579.1536[C_30_H_26_O_12_+H]+(6.71)  427.1059[C_22_H_18_O_9_+H]+(8.31)  291.0883[C_15_H_13_O_6_+H+H]+(7.01)  271.0606[C_15_H_11_O_5_-e]+(2.05) | E |
| 28 | 2.90 | 565.1567 | M+H | C_26_H_28_O_14_ | 2.62 | Kaempferol 3-arabinofuranoside 7-rhamnofuranoside | 587.1369[C_26_H_28_O_14_+Na]+(-0.37)  565.1599[C_26_H_28_O_14_+H]+(8.38)  481.1165[C_25_H_22_O_10_-e-H]+(7.41)  349.0939[C_17_H_15_O_8_+H+H]+(6.00) | B |
| 29 | 3.10 | 773.2167 | M+H | C_33_H_40_O_21_ | 4.22 | Quercetin 3-rutinoside 7-galactoside | 773.2127[C_33_H_40_O_21_+H]+(-0.99)  612.1712[C_27_H_30_O_16_+H+H]+(4.45)  465.1074[C_21_H_20_O_12_+H]+(9.99)  303.0708[C_12_H_16_O_9_-e-H]+(-0.93) | D |
| 30 | 3.18 | 180.1011 | M+H | C_10_H_13_NO_2_ | -4.38 | Phenprobamate | 180.1033[C_10_H_13_NO_2_+H]+(8.17)  162.0927[C_10_H_12_NO-e-H]+(8.21) | C, D |
| 31 | 3.18 | 162.0907 | M+H | C_10_H_11_NO | -4.27 | Tryptophanol | 162.0916[C_10_H_11_NO+H]+(-1.56)  132.0804[C_9_H_8_N+H+H]+(-3.09)  77.0388[C_6_H_4_+H]+(3.54) | D |
| 32 | 3.24 | 611.1637 | M+H | C_27_H_30_O_16_ | 4.91 | Rutin | 611.1580[C_27_H_30_O_16_+H]+(-4.35)  465.0969[C_21_H_20_O_12_+H]+(-12.65)  303.0487[C_15_H_10_O_7_+H]+(-4.09)  71.0491[C_4_H_6_O+H]+(-0.39) | A, C, D |
| 33 | 3.25 | 303.0499 | M+H | C_15_H_10_O_7_ | -0.01 | Quercetin | 303.0517[C_15_H_10_O_7_+H]+(5.78)  229.0505[C_13_H_7_O_4_+H+H]+(4.19)  153.0165[C_7_H_4_O_4_+H]+(-11.34) | C, D |
| 34 | 3.26 | 883.1765 | M+H | C_44_H_34_O_20_ | 5.54 | 3,3'-Digalloylprocyanidin B2 | 883.1802[C_44_H_34_O_20_+H]+(9.81)  563.1163[C_29_H_22_O_12_+H]+(-3.74)  287.0547[C_15_H_10_O_6_+H]+(-0.79)  247.0612[C_13_H_10_O_5_+H]+(4.75) | E |
| 35 | 3.31 | 725.2282 | M+H | C_33_H_40_O_18_ | -0.75 | 3,3',4'-Trihydroxyflavone 3-O-[a-L-rhamnopyranosyl-(1->2)[a-L-rhamnopyranosyl-(1->6)]-b-D-glucopyranoside] | 725.2223[C_33_H_40_O_18_+H]+(-8.94)  563.1735[C_27_H_29_O_13_+H+H]+(-4.29)  531.1555[C_26_H_26_O_12_+H]+(11.00)  395.0789[C_21_H_15_O_8_-e]+(6.93) | C |
| 36 | 3.32 | 271.0599 | M+H | C_15_H_10_O_5_ | -0.62 | 3,4',7-Trihydroxyflavone | 271.0577[C_15_H_10_O_5_+H]+(-8.85)  225.0538[C_14_H_8_O_3_+H]+(-3.44)  197.0584[C_13_H_7_O_2_+H+H]+(-6.42)  77.0376[C_6_H_3_+H+H]+(-12.71) | E |
| 37 | 3.34 | 447.1294 | M+Na | C_20_H_24_O_10_ | 7.61 | (1'x,2S)-2-(1,2-Dihydroxy-1-methylethyl)-2,3-dihydro-7H-furo[3,2-g][1]benzopyran-7-one 2'-glucoside | 447.1215[C_20_H_24_O_10_+Na]+(-10.29)  285.0695[C_14_H_13_O_5_+Na+H]+(-13.34)  253.0488[C_13_H_11_O_4_+Na-H]+(6.63)  213.0545[C_13_H_9_O_3_-e]+(-0.76) | A |
| 38 | 3.35 | 193.0488 | M+H | C_10_H_8_O_4_ | -4.03 | Scopoletin | 178.0233[C_9_H_5_O_4_+H]+(-15.77)  149.0226[C_8_H_5_O_3_-e]+(-5.26)  133.0273[C_8_H_5_O_2_-e]+(-9.01) | D |
| 39 | 3.39 | 443.0973 | M+H | C_22_H_18_O_10_ | 0.05 | (-)-Epicatechin gallate | 443.1022[C_22_H_18_O_10_+H]-(11.12)  317.0648[C_16_H_13_O_7_-e]+(-2.31)  255.0648[C_15_H_11_O_4_-e]+(-1.54)  123.0449[C_7_H_6_O_2_+H]-(6.50) | E |
| 40 | 3.46 | 579.1680 | M+Na | C_25_H_32_O_14_ | -0.83 | 10-Hydroxyoleuropein | 579.1690[C_25_H_32_O_14_+Na]+(1.01)  433.1180[C_21_H_22_O_10_-e-H]+(11.54)  399.1058[C_19_H_21_O_8_+Na-H]+(1.89)  301.0702[C_14_H_15_O_6_+Na-H]+(6.44) | C |
| 41 | 3.48 | 463.1246 | M+H | C_22_H_22_O_11_ | 2.34 | Leptosin | 463.1283[C_22_H_22_O_11_+H]+(10.34)  301.0707[C_16_H_12_O_6_+H]+(0.01)  259.0622[C_14_H_10_O_5_+H]+(8.06)  217.0483[C_12_H_7_O_4_+H+H]+(-5.91) | E |
| 42 | 3.62 | 419.1344 | M+Na | C_19_H_24_O_9_ | 8.06 | Aloesol 7-glucoside | 419.1340[C_19_H_24_O_9_+Na]+(6.64)  401.1256[C_19_H_22_O_8_+Na]+(12.11)  217.0481[C_10_H_11_O_4_+Na-H]+(4.63) | E |
| 43 | 3.66 | 369.1175 | M+Na | C_15_H_22_O_9_ | 5.59 | 1-(3-Hydroxy-4-Methoxyphenyl)-1,2-ethanediol 3'-O-b-D-glucoside | 369.1174[C_15_H_22_O_9_+Na]+(4.82)  295.0823[C_12_H_16_O_7_+Na]+(11.91)  207.0609[C_9_H_11_O_4_+Na+H]+(-9.14)  147.0414[C_7_H_8_O_2_+Na]+(-1.52) | C |
| 44 | 3.79 | 625.1782 | M+H | C_28_H_32_O_16_ | 3.08 | Isorhamnetin 3-O-[b-D-glucopyranosyl-(1->2)-a-L-rhamnopyranoside] | 625.1790[C_28_H_32_O_16_+H]+(4.28)  479.0877[C_21_H_20_O_13_-e]+(11.81)  317.0620[C_16_H_11_O_7_+H]+(-11.24)  129.0549[C_6_H_7_O_3_+H]+(2.63) | D |
| 45 | 3.94 | 225.0750 | M+H | C_11_H_12_O_5_ | -3.55 | Sinapic acid | 225.0744[C_11_H_12_O_5_+H]+(-6.11)  183.0628[C_9_H_9_O_4_+H+H]+(-12.95)  151.0412[C_8_H_6_O_3_+H]+(15.02) | C |
| 46 | 3.95 | 165.0534 | M+H | C_9_H_8_O_3_ | -7.55 | cis-p-Coumaric acid | 165.0583[C_9_H_8_O_3_+H]+(22.31)  137.0625[C_8_H_7_O_2_+H+H]+(20.27)  105.0356[C_7_H_4_O+H]+(20.13)  94.0419[C_6_H_5_O_3_+H]+(6.72) | C |
| 47 | 3.95 | 207.0645 | M+H | C_11_H_10_O_4_ | -3.28 | Citropten | 207.0670[C_11_H_10_O_4_+H]+(8.89)  179.0718[C_10_H_10_O_3_+H]+(8.55)  175.0393[C_10_H_7_O_3_-e]+(1.77) | C |
| 48 | 4.05 | 315.0497 | M+H | C_16_H_10_O_7_ | -0.71 | Laccaic acid D | 315.0485[C_16_H_10_O_7_+H]+(-4.71)  241.0496[C_14_H_7_O_4_+H+H]+(0.12)  199.0394[C_12_H_5_O_3_+H+H]+(1.89) | E |
| 49 | 4.35 | 313.0704 | M+Na | C_15_H_14_O_6_ | 7.54 | (+)-Epicatechin | 313.0724[C_15_H_14_O_6_+Na]+(13.21)  295.0577[C_15_H_12_O_5_+Na]+(-0.04)  270.0538[C_13_H_11_O_5_+Na]+(14.63) | E |
| 50 | 4.41 | 191.0697 | M+H | C_11_H_10_O_3_ | -3.24 | 7-Hydroxy-2,5-dimethyl-4H-1-benzopyran-4-one | 189.0562[C_11_H_10_O_3_-e-H]+(8.26)  176.0475[C_10_H_7_O_3_+H]+(3.85)  161.0597[C_10_H_9_O_2_-e]+(-0.08) | E |
| 51 | 4.46 | 233.0804 | M+H | C_13_H_12_O_4_ | -1.68 | Cassiachromone | 233.0830[C_13_H_12_O_4_+H]+(9.08)  200.0497[C_12_H_7_O_3_+H]+(14.68)  187.0722[C_12_H_10_O_2_+H]+(-17.20) | E |
| 52 | 4.46 | 287.0537 | M+H | C_15_H_10_O_6_ | -4.54 | Citreorosein | 287.0562[C_15_H_10_O_6_+H]+(4.20)  241.0480[C_14_H_8_O_4_+H]+(-6.46)  165.0172[C_8_H_4_O_4_+H]+(-6.45) | E |
| 53 | 4.65 | 275.0910 | M+H | C_15_H_14_O_5_ | -1.54 | (Z)-4-Methoxy-3,3',5,5'-tetrahydroxystilbene | 257.0843[C_15_H_13_O_4_-e]+(13.26)  157.0637[C_11_H_7_O+H+H]+(-6.92)  131.0484[C_9_H_5_O+H+H]+(-6.03) | E |
| 54 | 4.91 | 433.1119 | M+H | C_21_H_20_O_10_ | -2.40 | Anthraglycoside B | 415.1009[C_21_H_19_O_9_-e]+(-3.59)  397.0966[C_21_H_17_O_8_-e]+(12.18)  271.0595[C_15_H_9_O_5_+H+H]+(-2.20)  253.0487[C_15_H_9_O_4_-e]+(-3.52) | A, E |
| 55 | 5.01 | 481.1355 | M+H | C_22_H_24_O_12_ | 3.04 | 4'-O-Methyl-(-)-epicatechin 3'-O-glucuronide | 503.1178[C_22_H_24_O_12_+Na]+(3.59)  481.1311[C_22_H_24_O_12_+H]+(-6.12)  233.0814[C_13_H_12_O_4_+H]+(2.07)  215.0670[C_13_H_10_O_3_+H]+(-15.44) | E |
| 56 | 5.27 | 445.1129 | M+Na | C_20_H_22_O_10_ | 5.71 | Rumexoside | 445.1185[C_20_H_22_O_10_+Na]+(-4.49)  427.0952[C_20_H_20_O_9_+Na]+(-10.95)  153.0166[C_5_H_6_O_4_+Na]+(5.20) | E |
| 57 | 5.31 | 301.1075 | M+H | C_17_H_16_O_5_ | 1.57 | Methylnissolin | 301.1033[C_17_H_16_O_5_+H]+(-12.54)  197.0575[C_13_H_8_O_2_+H]+(-10.99)  106.0417[C_7_H_4_O+H+H]+(3.73) | A |
| 58 | 5.37 | 285.0759 | M+H | C_16_H_12_O_5_ | 0.40 | Glycitein | 285.0754[C_16_H_12_O_5_+H]+(-1.29)  270.0504[C_15_H_9_O_5_+H]+(-6.78)  213.0547[C_13_H_8_O_3_+H]+(0.52) | A |
| 59 | 5.41 | 517.1344 | M+H | C_25_H_24_O_12_ | 0.76 | Formononetin 7-(6''-malonylglucoside) | 517.1321[C_25_H_24_O_12_+H]+(-3.74)  269.0807[C_16_H_11_O_4_+H+H]+(-0.59)  226.0642[C_14_H_10_O_3_-e]+(7.84) | A |
| 60 | 5.45 | 893.3143 | 2M+H | C_23_H_26_O_9_ | -9.33 | Osmanthuside A | 875.3145[C_23_H_25_O_8_-e+M]+(2.76)  843.2825[C_22_H_21_O_7_-e+M]+(-3.92)  207.0642[C_11_H_10_O_4_+H]+(-4.67)  165.0533[C_9_H_7_O_3_+H+H]+(-7.55) | C |
| 61 | 5.60 | 315.0864 | M+H | C_17_H_14_O_6_ | 0.34 | 4',7-Dihydroxy-2',5-dimethoxyisoflavone | 315.0879[C_17_H_14_O_6_+H]+(5.22)  300.0637[C_16_H_11_O_6_+H]+(2.71)  243.0654[C_14_H_9_O_4_+H+H]+(0.79)  134.0360[C_8_H_5_O_2_+H]+(-1.98) | A |
| 62 | 5.65 | 271.0597 | M+H | C_15_H_10_O_5_ | -1.32 | 3'-Hydroxydaidzein | 271.0624[C_15_H_10_O_5_+H]+(8.60)  241.0538[C_14_H_9_O_4_-e]+(17.76)  229.0529[C_13_H_8_O_4_+H]+(14.39) | E |
| 63 | 5.68 | 441.1167 | M+Na | C_21_H_22_O_9_ | 2.60 | Aloin | 423.1046[C_21_H_20_O_8_+Na]+(-1.10)  321.0737[C_17_H_14_O_5_+Na]+(1.28)  185.0419[C_6_H_11_O_5_+Na-H]+(-0.40) | E |
| 64 | 5.68 | 257.0804 | M+H | C_15_H_12_O_4_ | -1.69 | Emodinanthranol | 257.0824[C_15_H_12_O_4_+H]+(5.80)  229.0878[C_14_H_12_O_3_+H]+(8.26)  211.0774[C_14_H_10_O_2_+H]+(9.48)  199.0406[C_12_H_6_O_3_+H]+(8.10) | E |
| 65 | 5.69 | 247.0963 | M+H | C_14_H_14_O_4_ | -0.72 | Torachrysone | 247.0938[C_14_H_14_O_4_+H]+(-10.73)  214.0623[C_13_H_9_O_3_+H]+(-0.75)  186.0712[C_12_H_9_O_2_+H]+(19.38)  147.0469[C_9_H_6_O_2_+H]+(19.24) | E |
| 66 | 5.69 | 409.1503 | M+H | C_20_H_24_O_9_ | 2.39 | Torachrysone 8-glucoside | 409.1498[C_20_H_24_O_9_+H]+(1.23)  391.1360[C_20_H_23_O_8_-e]+(-6.91)  247.0981[C_14_H_13_O_4_+H+H]+(6.44)  205.0861[C_12_H_12_O_3_+H]+(1.26) | E |
| 67 | 5.70 | 593.1875 | M+Na | C_26_H_34_O_14_ | 5.99 | 2''-Methoxy-(S)-oleuropein | 575.1700[C_26_H_32_O_13_+Na]+(-6.15)  491.1540[C_22_H_28_O_11_+Na]+(3.26)  351.1074[C_15_H_19_O_8_+Na+H]+(6.84)  151.0374[C_6_H_7_O_3_+Na+H]+(5.82) | C |
| 68 | 5.70 | 611.1967 | M+H | C_28_H_34_O_15_ | -0.54 | Hesperidin | 561.1671[C_27_H_28_O_13_+H]+(12.19)  277.0677[C_14_H_11_O_6_+H+H]+(-10.68)  193.0490[C_10_H_10_O_4_-e-H]+(-2.88)  165.0549[C_9_H_10_O_3_-e-H]+(1.33) | C |
| 69 | 5.74 | 439.1007 | M+Na | C_21_H_20_O_9_ | 1.90 | Chrysophanein | 439.0971[C_21_H_20_O_9_+Na]+(-6.48)  277.0476[C_15_H_9_O_4_+Na+H]+(1.87)  185.0425[C_6_H_11_O_5_+Na-H]+(2.81) | E |
| 70 | 5.74 | 543.1488 | M+H | C_27_H_26_O_12_ | -1.62 | Resveratrol 4'-(2-galloylglucoside) | 543.1501[C_27_H_26_O_12_+H]+(0.75)  252.0631[C_12_H_10_O_6_+H+H]+(0.87)  131.0503[C_9_H_5_O+H+H]+(8.64) | C |
| 71 | 5.79 | 455.0955 | M+Na | C_21_H_20_O_10_ | 1.46 | Kaempferol 3-O-alpha-L-rhamnofuranoside | 455.0919[C_21_H_20_O_10_+Na]+(-6.51)  293.0428[C_15_H_9_O_5_+Na+H]+(2.71)  185.0423[C_8_H_9_O_5_-e]+(-11.72) | E |
| 72 | 5.90 | 439.1009 | M+Na | C_21_H_20_O_9_ | 5.42 | Daidzin | 439.0994[C_21_H_20_O_9_+Na]+(-1.13)  417.1123[C_21_H_20_O_9_+H]+(-13.60)  277.0473[C_15_H_9_O_4_+Na+H]+(0.66) | A, E |
| 73 | 6.00 | 445.1137 | M+H | C_22_H_20_O_10_ | 1.75 | Betavulgarin xyloside | 445.1189[C_22_H_20_O_10_+H]+(13.59)  427.1023[C_22_H_19_O_9_-e]+(-0.06)  285.0760[C_16_H_11_O_5_+H+H]+(0.96)  270.0496[C_15_H_10_O_5_-e]+(-9.96) | A, E |
| 74 | 6.17 | 495.1498 | M+Na | C_21_H_28_O_12_ | 5.22 | 5'-((Z)-Feruloyl) 3-(2'-methylarabinosylxylose) | 495.1518[C_21_H_28_O_12_+Na]+(9.04)  477.1415[C_21_H_26_O_11_+Na]+(10.04)  459.1293[C_21_H_25_O_10_+Na-H]+(6.86)  417.1135[C_19_H_22_O_9_+Na]+(-5.02) | E |
| 75 | 6.17 | 247.0960 | M+H | C_14_H_14_O_4_ | -2.13 | Aegelinol | 247.0940[C_14_H_14_O_4_+H]+(-10.05)  214.0649[C_13_H_9_O_3_+H]+(11.16)  128.0645[C_10_H_7_+H]+(18.56) | E |
| 76 | 6.22 | 317.1018 | M+H | C_17_H_16_O_6_ | -0.46 | Artocarpanone A | 317.1064[C_17_H_16_O_6_+H]+(13.90)  289.1056[C_16_H_16_O_5_+H]+(-5.24)  163.0397[C_9_H_6_O_3_+H]+(4.27)  107.0487[C_7_H_5_O+H+H]+(-4.82) | A |
| 77 | 6.36 | 495.1513 | M+Na | C_21_H_28_O_12_ | 8.38 | b-D-fructosyl-a-D-(6-O-(E))-feruloylglucoside | 477.1387[C_21_H_26_O_11_+Na]+(4.09)  459.1454[C_20_H_27_O_12_-e]+(-9.38)  205.0872[C_12_H_12_O_3_-e+H]+(6.03)  127.0368[C_4_H_8_O_3_+Na]+(1.87) | E |
| 78 | 6.41 | 469.1102 | M+Na | C_22_H_22_O_10_ | -0.77 | Physcionin | 365.0982[C_21_H_17_O_6_-e]+(-10.18)  347.0717[C_17_H_14_O_8_-e+H]+(-12.69)  295.0836[C_14_H_14_O_7_-e+H]+(8.12)  185.0461[C_8_H_10_O_5_-e-H]+(8.97) | E |
| 79 | 6.93 | 411.1535 | 2M+H | C_11_H_11_NO_3_ | -3.85 | Cinnamoylglycine | 351.1351[C_9_H_8_NO-e+M]+(3.21)  188.0707[C_11_H_10_NO-e]+(12.07)  170.0604[C_11_H_9_NO-e-H]+(2.02) | C |
| 80 | 7.03 | 333.2642 | M+H | C_18_H_36_O_5_ | 1.96 | 9,10,13-Trihydroxystearic acid | 333.2635[C_18_H_36_O_5_+H]+(-0.17)  315.2566[C_18_H_35_O_4_-e]+(11.55)  297.2431[C_18_H_33_O_3_-e]+(2.19) | C |
| 81 | 7.13 | 429.1188 | M+Na | C_20_H_22_O_9_ | 7.93 | Astringin(Piceatannol-3'-O-D-glucopyranoside) | 429.1181[C_20_H_22_O_9_+Na]+(5.71)  385.0854[C_18_H_18_O_8_+Na]+(-10.55)  313.0681[C_17_H_14_O_6_-e-H]+(-8.30) | B, C, D |
| 82 | 7.28 | 295.2269 | M+Na | C_16_H_32_O_3_ | 9.32 | 12-Hydroxyhexadecanoic acid | 295.2215[C_16_H_32_O_3_+Na]+(-9.46)  278.2207[C_16_H_31_O_2_+Na]+(-3.30)  231.2076[C_15_H_29_+Na-H]+(-3.00) | A, B, C, D, E |
| 83 | 7.44 | 167.0693 | M+H | C_9_H_10_O_3_ | -6.12 | 3-Hydroxy-1-(4-hydroxyphenyl)-1-propanone | 167.0712[C_9_H_10_O_3_+H]+(5.24)  134.0362[C_8_H_7_O_2_-e-H]+(-0.20)  120.0196[C_7_H_5_O_2_-e-H]+(-8.05) | A |
| 84 | 7.69 | 269.0812 | M+H | C_16_H_12_O_4_ | 1.40 | 8-Hydroxy-1-methoxy-3-methylanthraquinone | 269.0811[C_16_H_12_O_4_+H]+(1.00)  253.0519[C_15_H_9_O_4_-e]+(9.26)  237.0571[C_15_H_9_O_3_-e]+(10.52)  209.0617[C_14_H_7_O_2_+H+H]+(9.78) | E |
| 85 | 7.72 | 303.1225 | M+H | C_17_H_18_O_5_ | -0.61 | (R)-3',7-Dihydroxy-2',4'-dimethoxyisoflavan | 303.1225[C_17_H_18_O_5_+H]+(-0.49)  167.0698[C_9_H_10_O_3_+H]+(-2.89)  123.0436[C_7_H_6_O_2_+H]+(-3.86) | A |
| 86 | 8.00 | 455.3527 | M+H | C_30_H_46_O_3_ | 1.58 | Desoxoglabrolide | 455.3531[C_30_H_46_O_3_+H]+(2.44)  437.3439[C_30_H_45_O_2_-e]+(5.60) | A, C |
| 87 | 8.00 | 473.3628 | M+Na | C_28_H_50_O_4_ | 5.83 | 6-Deoxocastasterone | 456.3609[C_28_H_49_O_3_+Na]+(7.56)  455.3491[C_28_H_48_O_3_+Na]+(-0.88)  437.3342[C_28_H_47_O_2_+Na-H]+(-10.96)  143.1064[C_8_H_14_O_2_-e+H]+(-2.08) | A |
| 88 | 8.16 | 285.0394 | M+H | C_15_H_8_O_6_ | 0.23 | Rhein | 285.0388[C_15_H_8_O_6_+H]+(-1.96)  270.0542[C_15_H_8_O_5_+H+H]+(7.01)  241.0539[C_14_H_7_O_4_+H+H]+(17.79) | E |
| 89 | 8.30 | 291.1954 | M+H | C_18_H_26_O_3_ | -0.16 | Octyl 4-methoxycinnamic acid | 273.1882[C_18_H_26_O_2_-e-H]+(11.90)  249.1448[C_15_H_19_O_3_+H+H]+(-14.85)  135.0805[C_9_H_9_O+H+H]+(15.24) | A, C, D |
| 90 | 8.57 | 419.3310 | M+Na | C_28_H_44_O | 6.58 | Ergosterol | 419.3268[C_28_H_44_O+Na]+(-3.91)  401.3190[C_28_H_42_+Na]+(2.61)  195.1149[C_13_H_16_+Na]+(2.45) | A |
| 91 | 8.60 | 473.3623 | M+H | C_30_H_48_O_4_ | -0.53 | 20beta-Hydroxyursolic acid | 455.3466[C_30_H_47_O_3_-e]+(-11.71)  437.3416[C_30_H_45_O_2_-e]+(0.37)  141.1272[C_9_H_16_O+H]+(-1.49) | A, C |
| 92 | 8.86 | 279.2319 | M+Na | C_16_H_32_O_2_ | 9.52 | Isopalmitic acid | 279.2336[C_16_H_32_O_2_+Na]+(15.12)  261.2194[C_16_H_30_O+Na]+(1.68)  209.1512[C_11_H_21_O_2_+Na+H]+(3.62) | A, C |
| 93 | 8.90 | 441.3731 | M+H | C_30_H_48_O_2_ | 0.78 | Soyasapogenol C | 441.3664[C_30_H_48_O_2_+H]+(-14.30)  424.3699[C_30_H_47_O+H]+(-0.10)  383.3314[C_27_H_42_O+H]+(1.26) | A |
| 94 | 9.01 | 455.3521 | M+H | C_30_H_46_O_3_ | 0.32 | beta-Elemonic acid | 437.3427[C_30_H_45_O_2_-e]+(2.81)  419.3308[C_30_H_44_O-e-H]+(-0.02)  143.1065[C_8_H_13_O_2_+H+H]+(-1.19) | A |
| 95 | 9.02 | 809.4713 | M+Na | C_41_H_70_O_14_ | 7.05 | Vinaginsenoside R11 | 791.4569[C_41_H_68_O_13_+Na]+(2.13)  629.3969[C_35_H_58_O_8_+Na]+(-8.75)  437.3378[C_28_H_45_O_2_+Na+H]+(-2.79)  297.2223[C_19_H_29_O+Na+H]+(11.44) | A |
| 96 | 9.52 | 277.2160 | M+Na | C_16_H_30_O_2_ | 8.76 | Palmitelaidic acid | 259.2074[C_16_H_28_O+Na]+(16.00)  221.1482[C_12_H_21_O_2_+Na+H]+(-13.55)  149.1309[C_9_H_17_+Na+H]+(5.46)  121.1003[C_7_H_14_+Na]+(12.06) | A, C |
| 97 | 9.52 | 295.2269 | M+H | C_18_H_30_O_3_ | 0.56 | 12,13-Epoxy-9,15-octadecadienoic acid | 295.2280[C_18_H_30_O_3_+H]+(4.03)  277.2123[C_18_H_29_O_2_-e]+(-9.92)  259.2050[C_18_H_28_O-e-H]+(-2.45) | A |
| 98 | 9.72 | 455.3518 | M+H | C_30_H_46_O_3_ | -0.36 | 9(11)-Dehydroglycyrrhetic acid | 455.3490[C_30_H_46_O_3_+H]+(-6.54)  419.3343[C_30_H_43_O-e]+(8.20) | A, C |
| 99 | 9.73 | 421.3460 | M+Na | C_28_H_46_O | 4.74 | 22,23-Dihydroergosterol | 421.3484[C_28_H_46_O+Na]+(10.27)  403.3310[C_28_H_44_+Na]+(-6.34)  363.3005[C_25_H_40_+Na]+(-4.80) | A |
| 100 | 10.12 | 271.0601 | M+H | C_15_H_10_O_5_ | -0.14 | 2'-Hydroxydaidzein | 271.0607[C_15_H_10_O_5_+H]+(2.30)  225.0531[C_14_H_8_O_3_+H]+(-6.76) | E |
| 101 | 10.56 | 279.2320 | M+Na | C_16_H_32_O_2_ | 9.80 | Palmitic acid | 209.1519[C_11_H_21_O_2_+Na+H]+(3.40)  137.1318[C_8_H_17_+Na+H]+(11.95) | A, C |
| 102 | 10.66 | 295.2270 | M+Na | C_16_H_32_O_3_ | 9.56 | 13-Hydroxyhexadecanoic acid | 277.2179[C_16_H_30_O_2_+Na]+(14.59)  179.1382[C_10_H_21_O+Na-H]+(-13.60)  105.0695[C_6_H_11_+Na-H]+(18.80) | A, B |
| 103 | 10.67 | 455.3525 | M+H | C_30_H_46_O_3_ | 1.14 | Glypallidifloric acid | 455.3551[C_30_H_46_O_3_+H]+(6.93)  191.1807[C_14_H_22_+H]+(6.45)  123.1165[C_9_H_14_+H]+(-3.05)  109.1015[C_8_H_11_+H+H]+(2.91) | A, C |
| 104 | 11.11 | 281.2473 | M+H | C_18_H_32_O_2_ | -0.58 | Linoleic acid | 281.2456[C_18_H_32_O_2_+H]+(-6.65)  245.2260[C_18_H_30_-e-H]+(-1.57)  179.1787[C_13_H_23_-e]+(-4.05) | A, C |
| 105 | 11.17 | 468.3099 | M+H | C_29_H_41_NO_4_ | -1.97 | N-Arachidonoyl tyrosine | 935.6143[C_29_H_41_NO_4_+H+M]+(-0.12)  468.3100[C_29_H_41_NO_4_+H]+(-1.93)  285.2430[C_20_H_30_N+H]+(-7.22)  166.0636[C_9_H_9_O_3_+H]+(6.84) | A |
| 106 | 11.24 | 427.2810 | 2M+H | C_11_H_19_NO_3_ | 1.81 | 2-nonenoylglycine | 409.2731[C_11_H_18_NO_2_-e+M]+(8.20)  381.2784[C_10_H_18_NO-e+M]+(9.70)  298.2379[C_6_H_13_-e+M]+(0.70) | F |
| 107 | 11.69 | 489.3582 | M+H | C_30_H_48_O_5_ | 1.62 | Tormentic acid | 444.3607[C_29_H_47_O_3_+H]+(1.98)  425.3416[C_29_H_45_O_2_-e]+(0.45)  373.2688[C_24_H_36_O_3_+H]+(-13.15)  201.1611[C_15_H_21_-e]+(-13.17) | C |
| 108 | 11.76 | 455.3523 | M+Na | C_28_H_48_O_3_ | 6.35 | beta-Tocopheryl quinone | 409.3437[C_27_H_46_O+Na]+(-1.05)  391.3616[C_26_H_47_O_2_-e]+(11.71)  177.1623[C_11_H_23_+Na-H]+(5.18) | A, C |
| 109 | 11.77 | 473.3622 | M+H | C_30_H_48_O_4_ | -0.62 | delta-Maslinic acid | 473.3693[C_30_H_48_O_4_-e]+(14.18)  427.3585[C_29_H_47_O_2_-e]+(3.29)  219.1728[C_15_H_22_O+H]+(-6.98)  133.1009[C_10_H_14_-e-H]+(-2.07) | C |
| 110 | 11.87 | 542.3225 | M+Na | C_26_H_50_NO_7_P | 1.52 | 2-linoleoyl-sn-glycero-3-phosphocholine | 542.3230[C_26_H_50_NO_7_P+Na]+(2.36)  483.2487[C_23_H_41_O_7_P+Na]+(0.97)  439.2142[C_20_H_36_NO_6_P+Na-H]+(10.96)  95.0835[C_5_H_11_+Na+H]+(3.87) | A, B, D, E, F |
| 111 | 11.88 | 471.3475 | M+H | C_30_H_46_O_4_ | 1.32 | Rubinic acid | 471.3476[C_30_H_46_O_4_+H]+(1.59)  235.1659[C_15_H_22_O_2_+H]+(-14.45)  217.1573[C_15_H_20_O+H]+(-6.40)  205.1584[C_14_H_20_O+H]+(-1.27) | C |
| 112 | 12.02 | 285.0758 | M+H | C_16_H_12_O_5_ | 0.05 | Emodin-3-methyl ether | 285.0758[C_16_H_12_O_5_+H]+(0.21)  267.0695[C_16_H_11_O_4_-e]+(16.35)  252.0425[C_15_H_7_O_4_+H]+(3.19) | E |
| 113 | 12.07 | 317.2089 | M+H | C_20_H_28_O_3_ | -7.07 | Cafestol | 257.1897[C_18_H_24_O+H]+(-0.97)  161.0961[C_11_H_14_O-e-H]+(0.11)  135.0779[C_9_H_10_O+H]+(-18.76) | A |
| 114 | 12.07 | 179.1419 | M+Na | C_10_H_20_O | 8.03 | p-Menthan-1-ol | 179.1442[C_10_H_20_O+Na]+(19.96)  133.1002[C_8_H_14_+Na]+(10.05)  113.0966[C_7_H_13_O-e]+(4.68) | A |
| 115 | 12.09 | 473.3626 | M+H | C_30_H_48_O_4_ | 0.12 | Maslinic acid | 473.3564[C_30_H_48_O_4_+H]+(-13.15)  428.3661[C_29_H_47_O_2_+H]+(2.83)  203.1801[C_15_H_22_+H]+(3.22) | C |
| 116 | 12.13 | 295.2269 | M+H | C_18_H_30_O_3_ | 0.49 | 12(13)-epoxy-6Z,9Z-octadecadienoic acid | 295.2254[C_18_H_30_O_3_+H]+(-4.55)  278.2238[C_18_H_29_O_2_+H]+(-0.94)  163.1482[C_12_H_17_+H+H]+(0.10)  81.0702[C_6_H_8_+H]+(4.44) | A, B, C, D, E |
| 117 | 12.22 | 277.2163 | M+Na | C_16_H_30_O_2_ | 9.85 | Palmitoleic acid | 277.2114[C_16_H_30_O_2_+Na]+(-8.86)  235.1668[C_13_H_23_O_2_+Na+H]+(-2.90)  179.1415[C_12_H_19_O-e]+(-8.86)  121.0995[C_7_H_14_+Na]+(5.82) | A, B, C, D, E |
| 118 | 12.34 | 317.2089 | M+Na | C_18_H_30_O_3_ | -0.55 | 2-Hydroxylinolenic acid | 317.2084[C_18_H_30_O_3_+Na]+(-1.09)  277.2159[C_18_H_29_O_2_-e]+(-1.33)  207.1385[C_13_H_19_O_2_-e]+(2.82)  145.0987[C_9_H_14_+Na]+(-0.56) | A, C |
| 119 | 12.64 | 297.2425 | M+H | C_18_H_32_O_3_ | 0.18 | 13S-hydroxyoctadecadienoic acid | 297.2439[C_18_H_32_O_3_+H]+(5.01)  183.1391[C_11_H_18_O_2_+H]+(5.88)  169.1607[C_11_H_19_O+H+H]+(11.91) | A, B |
| 120 | 12.85 | 641.3831 | M+Na | C_39_H_54_O_6_ | 2.98 | cis-p-Coumaroylcorosolic acid | 641.3834[C_39_H_54_O_6_+Na]+(3.34)  560.3434[C_34_H_49_O_5_+Na]+(-6.85)  459.2548[C_28_H_36_O_4_+Na]+(9.08) | C |
| 121 | 12.96 | 619.4013 | M+H | C_39_H_54_O_6_ | 3.17 | 3-O-cis-Coumaroylmaslinic acid | 619.3995[C_39_H_54_O_6_+H]+(0.32)  455.3542[C_30_H_47_O_3_-e]+(4.91)  235.1668[C_15_H_22_O_2_+H]+(-10.69)  147.0444[C_9_H_7_O_2_-e]+(2.39) | C |
| 122 | 13.08 | 671.3941 | M+Na | C_40_H_56_O_7_ | 6.13 | trans-3-Feruloylcorosolic acid | 671.3959[C_40_H_56_O_7_+Na]+(6.04)  437.3436[C_30_H_45_O_2_-e]+(4.98)  201.1667[C_15_H_21_-e]+(14.44)  177.0547[C_10_H_9_O_3_-e]+(0.38) | F |
| 123 | 13.08 | 455.3524 | M+H | C_30_H_46_O_3_ | 0.86 | Ursonic acid | 437.3426[C_30_H_45_O_2_-e]+(2.53)  409.3451[C_29_H_45_O-e]+(-3.32)  189.1639[C_14_H_19_+H+H]+(0.87)  135.1160[C_10_H_13_+H+H]+(-6.28) | A, C |
| 124 | 13.20 | 546.3547 | M+Na | C_26_H_54_NO_7_P | 3.22 | 2-acetyl-1-alkyl-sn-glycero-3-phosphocholine | 546.3553[C_26_H_54_NO_7_P+Na]+(4.15)  487.2819[C_23_H_45_O_7_P+Na]+(4.80)  443.2549[C_21_H_41_O_6_P+Na]+(3.66) | A, F |
| 125 | 13.47 | 439.3581 | M+Na | C_28_H_48_O_2_ | 8.36 | Vitamin-E | 439.3501[C_28_H_48_O_2_+Na]+(-10.23)  205.1927[C_13_H_27_+Na-H]+(-0.01)  179.1772[C_11_H_23_+Na+H]+(0.92)  109.0992[C_6_H_13_+Na+H]+(3.52) | C, D |
| 126 | 13.48 | 457.3678 | M+H | C_30_H_48_O_3_ | 0.47 | Ursolic acid | 457.3686[C_30_H_48_O_3_+H]+(2.15)  411.3670[C_29_H_47_O-e]+(11.80)  203.1773[C_15_H_22_+H]+(-10.60) | C |
| 127 | 13.49 | 263.2373 | M+H | C_18_H_30_O | 1.38 | Farnesyl acetone | 263.2411[C_18_H_30_O+H]+(15.85)  179.1802[C_13_H_22_+H]+(3.82)  141.1251[C_9_H_15_O+H+H]+(-15.97) | A, E |
| 128 | 13.78 | 305.2476 | M+H | C_20_H_32_O_2_ | 0.39 | Cis-8,11,14,17-Eicosatetraenoic acid | 305.2505[C_20_H_32_O_2_+H]+(9.87)  287.2342[C_20_H_31_O-e]+(-9.61)  221.1532[C_14_H_22_O_2_-e-H]+(-2.19)  121.1017[C_9_H_14_-e-H]+(4.53) | F |
| 129 | 13.81 | 282.2798 | M+H | C_18_H_35_NO | 2.17 | Oleamide | 282.2804[C_18_H_35_NO+H]+(4.40)  247.2408[C_18_H_32_-e-H]+(-4.86)  226.2139[C_14_H_26_NO+H+H]+(-11.64)  125.0958[C_8_H_13_O-e]+(-1.82) | A, B |
| 130 | 13.93 | 409.3466 | M+Na | C_27_H_46_O | 6.44 | Lathosterol | 409.3483[C_27_H_46_O+Na]+(10.13)  391.3342[C_27_H_44_+Na]+(1.65)  283.2435[C_19_H_32_+Na]+(13.59)  177.1607[C_11_H_22_+Na]+(-3.95) | C |
| 131 | 13.95 | 257.2469 | M+H | C_16_H_32_O_2_ | -2.34 | Ethyl tetradecanoate | 279.2337[C_16_H_32_O_2_+Na]+(15.23)  187.1688[C_11_H_21_O_2_+H+H]+(-2.75) | A, F |
| 132 | 14.06 | 265.2528 | M+H | C_18_H_32_O | 0.87 | (z)-9,17-octadecadienal | 265.2485[C_18_H_32_O+H]+(-15.35)  169.1602[C_11_H_19_O+H+H]+(8.84)  139.1113[C_9_H_16_O-e-H]+(-3.26) | C, E |
| 133 | 14.10 | 371.1004 | M+H | C_16_H_18_O_10_ | 8.52 | 5-Hydroxy-6-methoxycoumarin 7-glucoside | 356.0707[C_15_H_15_O_10_+H]+(-8.61)  207.0314[C_10_H_7_O_5_-e]+(12.81)  149.0442[C_5_H_10_O_5_-e-H]+(-1.45) | B, C, D, E |
| 134 | 14.15 | 443.3885 | M+H | C_30_H_50_O_2_ | 0.38 | Betulin | 443.3927[C_30_H_50_O_2_+H]+(9.68)  426.3810[C_30_H_49_O+H]+(-10.85)  235.2091[C_16_H_26_O+H]+(14.39)  191.1811[C_14_H_22_+H]+(8.69)  109.1004[C_8_H_11_+H+H]+(-7.16) | C |
| 135 | 14.36 | 427.3938 | M+H | C_30_H_50_O | 0.86 | Taraxerol | 427.3976[C_30_H_50_O+H]+(9.68)  409.3821[C_30_H_49_-e]+(-1.88)  191.1764[C_14_H_22_+H]+(-15.98) | C |
| 136 | 14.66 | 633.1500 | M+Na | C_27_H_30_O_16_ | 9.26 | Quercetin 7-rutinoside | 633.1356[C_27_H_30_O_16_+Na]+(-11.06)  434.0798[C_18_H_19_O_11_+Na]+(-4.95)  270.0132[C_12_H_7_O_6_+Na]+(-1.25) | B, D |
| 137 | 14.95 | 401.3425 | M+H | C_27_H_44_O_2_ | 2.85 | 7-Ketocholesterol | 401.3459[C_27_H_44_O_2_+H]+(11.31)  229.1554[C_16_H_20_O+H]+(-14.32)  175.1097[C_12_H_14_O+H]+(-11.88)  161.0939[C_11_H_12_O+H]+(-13.59) | F |
| 138 | 15.23 | 409.3835 | 2M+H | C_15_H_24_ | 1.41 | Benzylalcohol alpha-isobutyl-2,4,6-trimethyl(1-mestyl-3-methyl-1-butanol) | 409.3790[C_15_H_24_+H+M]+(-9.48)  205.1952[C_15_H_24_+H]+(0.56)  149.1324[C_11_H_15_+H+H]+(-0.41) | C |
| 139 | 15.23 | 469.4039 | M+H | C_32_H_52_O_2_ | -0.15 | alpha-Amyrin acetate | 409.3834[C_30_H_49_-e]+(1.25)  219.2084[C_16_H_26_+H]+(-10.82)  191.1787[C_14_H_23_-e]+(-3.79)  123.1159[C_9_H_16_-e-H]+(-7.49) | C |
| 140 | 17.26 | 786.6055 | M+H | C_44_H_84_NO_8_P | 6.10 | 1,2-dioleoyl-sn-glycero-3-phosphocholine | 808.5892[C_44_H_84_NO_8_P+Na]+(7.98)  749.5183[C_41_H_75_O_8_P+Na]+(12.13)  625.5184[C_39_H_71_O_4_+Na-H]+(2.80)  506.3579[C_26_H_51_NO_6_P+H+H]+(-5.01)  184.0748[C_5_H_13_NO_4_P+H+H]+(8.26) | A, B |

A: Astragaliradix; B: Pseudostellariae radix; C: Ligustri lucidi fructus; D: Lycii fructus; E: Rhei radix et rhizome; F: Hirudo

**Table S4. Components of KLX detected by UPLC-Q/TOF-MS in negative-ion mode.**

| **NO.** | **Rt / min** | **m/z** | **Adducts** | **Fomula** | **Error / ppm** | **Compouds Name** | **Mass Fragments** | **Source** |
| --- | --- | --- | --- | --- | --- | --- | --- | --- |
| 1 | 0.48 | 549.1642 | M+FA-H | C_18_H_32_O_16_ | -6.48 | Raffinose | 503.1584[C_18_H_32_O_16_+e-H]-(-6.74)  383.1166[C_14_H_23_O_12_+e]-(-7.64)  323.0958[C_12_H_19_O_10_+e]-(-8.00)  143.0344[C_6_H_8_O_4_-H]-(-4.03) | A, B |
| 2 | 0.49 | 711.2162 | M+FA-H | C_24_H_42_O_21_ | -2.63 | Stachyose | 665.2079[C_24_H_42_O_21_+e-H]-(-10.02)  485.1444[C_18_H_29_O_15_+e]-(-13.95)  383.1196[C_14_H_23_O_12_+e]-(0.21)  179.0550[C_6_H_11_O_6_+e]-(-6.20) | A, B, E |
| 3 | 0.88 | 341.1095 | M-H | C_12_H_22_O_11_ | 1.60 | Sophorose | 341.1072[C_12_H_22_O_11_+e-H]-(-5.14)  252.0858[C_9_H_16_O_8_+e]-(2.98)  131.0350[C_5_H_8_O_4_-H]-(-0.26) | A, B, E |
| 4 | 1.13 | 331.0672 | M-H | C_13_H_16_O_10_ | 0.28 | 3-Glucogallic acid | 331.0658[C_13_H_16_O_10_-H]-(-3.62)  271.0469[C_11_H_12_O_8_-H]-(3.74)  241.0359[C_10_H_10_O_7_-H]-(2.12)  151.0037[C_7_H_3_O_4_+e]-(0.59) | E |
| 5 | 1.41 | 353.0741 | M+FA-H | C_11_H_16_O_10_ | 5.07 | D-Erythroascorbic acid 1'-a-D-glucoside | 353.0743[C_11_H_16_O_10_+FA-H]-(5.03)  173.0068[C_5_H_5_O_4_+FA-H-H]-(-13.95)  155.0001[C_5_H_3_O_3_+FA-H-H]-(9.80) | D |
| 6 | 1.68 | 337.0779 | M-H | C_12_H_18_O_11_ | 0.88 | 2-O-alpha-D-Glucopyranosyl-L-ascorbic acid | 337.0746[C_12_H_18_O_11_-H]-(-8.83)  277.0553[C_10_H_13_O_9_+e]-(-4.40)  174.0163[C_6_H_7_O_6_-H]-(-3.92)  157.0145[C_6_H_5_O_5_+e]-(1.57) | D |
| 7 | 1.99 | 451.1203 | M-H | C_21_H_24_O_11_ | -9.52 | (+)-Catechin 6-C-glucoside | 451.1284[C_21_H_24_O_11_-H]-(8.48)  331.0812[C_17_H_16_O_7_-H]-(-3.37)  289.0718[C_15_H_13_O_6_+e]-(0.21)  151.0399[C_8_H_8_O_3_-H]-(-1.01) | E |
| 8 | 2.12 | 483.0747 | M-H | C_20_H_20_O_14_ | -6.96 | Gallic acid 3-O-(6-galloylglucoside) | 483.0724[C_20_H_20_O_14_-H]-(-11.73)  439.0862[C_19_H_19_O_12_+e]-(-4.71)  331.0685[C_13_H_15_O_10_+e]-(4.51)  271.0456[C_11_H_12_O_8_-H]-(-1.11) | E |
| 9 | 2.15 | 577.1330 | M-H | C_30_H_26_O_12_ | -3.76 | Procyanidin B2 | 577.1304[C_30_H_26_O_12_-H]-(-8.23)  451.1011[C_24_H_19_O_9_+e]-(-5.20)  407.0767[C_22_H_16_O_8_-H]-(-1.23)  289.0695[C_15_H_13_O_6_+e]-(-7.71) | E |
| 10 | 2.32 | 577.1333 | M-H | C_30_H_26_O_12_ | -3.17 | Epicatechin-(6'->8)-epicatechin | 451.1042[C_24_H_19_O_9_+e]-(1.77)  425.0839[C_22_H_18_O_9_-H]-(-9.32)  407.0761[C_22_H_16_O_8_-H]-(-2.81)  289.0719[C_15_H_13_O_6_+e]-(0.63) | E |
| 11 | 2.45 | 517.1531 | M+FA-H | C_21_H_28_O_12_ | -6.67 | 1-O-Cinnamoyl-beta-D-gentiobiose | 517.1539[C_21_H_28_O_12_+FA-H]-(-4.71)  337.0940[C_15_H_17_O_6_+FA-H-H]-(3.24)  235.0588[C_11_H_10_O_3_+FA-H]-(-10.39)  175.0397[C_9_H_7_O+FA-H-H]-(-2.20) | D |
| 12 | 2.73 | 547.1068 | M-H | C_25_H_24_O_14_ | -4.53 | Chrysoeriol 7-O-(6''-malonyl-glucoside) | 547.1081[C_25_H_24_O_14_-H]-(-2.25)  503.1181[C_24_H_23_O_12_+e]-(-2.87)  313.0579[C_13_H_14_O_9_-H]-(4.47) | E |
| 13 | 2.74 | 461.1055 | M+FA-H | C_21_H_20_O_9_ | -8.34 | Chrysophanol-8-O-β-D-glucopyranoside | 337.0752[C_19_H_13_O_6_+e]-(10.32)  299.0551[C_15_H_10_O_4_+FA-H]-(-3.30)  255.0649[C_14_H_9_O_2_+FA-H+H]-(-5.40) | E |
| 14 | 2.88 | 577.1344 | M-H | C_30_H_26_O_12_ | -1.24 | Procyanidin B1 | 577.1423[C_30_H_26_O_12_-H]-(12.39)  425.0912[C_22_H_18_O_9_-H]-(8.05)  289.0699[C_15_H_13_O_6_+e]-(-6.34) | E |
| 15 | 2.92 | 563.1382 | M-H | C_26_H_28_O_14_ | -4.24 | Isovitexin 2''-O-arabinoside | 563.1387[C_26_H_28_O_14_-H]-(-3.36)  503.1191[C_24_H_24_O_12_-H]-(-0.76)  443.0934[C_22_H_20_O_10_-H]-(-11.01) | B |
| 16 | 3.11 | 545.1266 | M+FA-H | C_25_H_24_O_11_ | -7.01 | Epicatechin pentaacetate | 545.1296[C_25_H_24_O_11_+FA-H]-(-0.96)  503.1228[C_23_H_21_O_10_+FA-H+H]-(6.50) | E |
| 17 | 3.29 | 881.1542 | M-H | C_44_H_34_O_20_ | -3.23 | Epiafzelechin 3-O-gallate-(4beta->6)-epigallocatechin 3-O-gallate | 881.1645[C_44_H_34_O_20_-H]-(8.47)  729.1427[C_37_H_29_O_16_+e]-(-4.73)  577.1025[C_29_H_22_O_13_-H]-(6.51)  407.0748[C_22_H_15_O_8_+e]-(-6.09) | E |
| 18 | 3.33 | 637.1759 | M+FA-H | C_31_H_28_O_12_ | -3.31 | 8,8'-Methylenebiscatechin | 637.1494[C_31_H_28_O_12_+FA-H]-(-10.81)  289.0700[C_15_H_13_O_6_+e]-(-6.02)  161.0236[C_9_H_6_O_3_+e-H]-(-5.05)  137.0257[C_7_H_6_O_3_-H]-(8.98) | A, E |
| 19 | 3.38 | 353.0876 | M-H | C_16_H_18_O_9_ | -0.61 | Chlorogenic acid | 191.0543[C_7_H_11_O_6_+e]-(-9.80)  135.0441[C_8_H_7_O_2_+e]-(-7.78) | D |
| 20 | 3.44 | 441.0789 | M-H | C_22_H_18_O_10_ | -8.69 | (-)-Epicatechin 3-O-gallate | 441.0769[C_22_H_18_O_10_-H]-(-13.26)  331.0428[C_16_H_13_O_8_-H-H]-(-9.39)  289.0694[C_15_H_13_O_6_+e]-(-8.03)  253.0498[C_15_H_11_O_4_-H-H]-(-3.24) | E |
| 21 | 3.45 | 609.1472 | M-H | C_27_H_30_O_16_ | 1.75 | Quercetin 3-(2-glucosylrhamnoside) | 609.1434[C_27_H_30_O_16_-H]-(-4.42)  343.0428[C_17_H_11_O_8_+e]-(-9.04)  271.0283[C_14_H_9_O_6_-H-H]-(12.90)  151.0026[C_7_H_4_O_4_-H]-(-7.38) | A |
| 22 | 3.51 | 729.1442 | M-H | C_37_H_30_O_16_ | -2.56 | Procyanidin B-5,3'-O-gallate | 729.1371[C_37_H_30_O_16_-H]-(-12.40)  577.0988[C_29_H_22_O_13_-H]-(0.05)  407.0797[C_22_H_16_O_8_-H]-(6.11)  289.0698[C_15_H_13_O_6_+e]-(-6.76) | E |
| 23 | 3.87 | 233.0825 | M-H | C_13_H_14_O_4_ | 2.35 | Aloesol | 233.0852[C_13_H_14_O_4_-H]-(3.95)  215.0748[C_13_H_12_O_3_-H]-(6.23)  189.0588[C_11_H_9_O_3_+e]-(16.10) | E |
| 24 | 4.01 | 477.1042 | M-H | C_22_H_22_O_12_ | 0.77 | 2-O-(4-Hydroxycinnamoyl)-1-O-galloyl-beta-D-glucopyranoside | 477.1016[C_22_H_22_O_12_-H]-(-4.68)  313.0556[C_13_H_13_O_9_+e]-(-2.92)  271.0419[C_11_H_12_O_8_-H]-(-14.73)  187.0391[C_11_H_8_O_3_-H]-(-5.14) | E |
| 25 | 4.36 | 447.0902 | M-H | C_21_H_20_O_11_ | -6.81 | Luteolin 7-glucoside | 447.0899[C_21_H_20_O_11_-H]-(-7.71)  327.0471[C_17_H_12_O_7_-H]-(-11.84)  285.0388[C_15_H_9_O_6_+e]-(-5.83)  256.0379[C_14_H_9_O_5_-H]-(0.70) | E |
| 26 | 4.45 | 189.0554 | M-H | C_11_H_10_O_3_ | -1.50 | (S)-2,3-Dihydro-5-hydroxy-2-methyl-1,4-naphthoquinone | 189.0592[C_11_H_10_O_3_-H]-(18.57)  162.0343[C_9_H_6_O_3_+e]-(12.67)  143.0528[C_10_H_8_O-H]-(18.09) | E |
| 27 | 4.50 | 431.0955 | M-H | C_21_H_20_O_10_ | -6.69 | Glucoemodin | 293.0447[C_17_H_10_O_5_-H]-(-2.78)  269.0433[C_15_H_9_O_5_+e]-(-8.45)  239.0379[C_14_H_9_O_4_-H-H]-(12.17) | E |
| 28 | 4.57 | 435.1062 | M-H | C_24_H_20_O_8_ | -5.34 | (-)-Epigallocatechin 3-cinnamate | 435.1076[C_24_H_20_O_8_-H]-(-2.04)  341.0664[C_18_H_13_O_7_+e]-(-0.62)  315.0852[C_17_H_14_O_6_+e+H]-(-7.12)  125.0245[C_6_H_5_O_3_+e]-(0.46) | E |
| 29 | 4.57 | 711.2175 | M+FA-H | C_31_H_38_O_16_ | 5.02 | Tetramethylquercetin 3-rutinoside | 665.1666[C_29_H_32_O_15_+FA-H]-(-8.53)  485.1445[C_25_H_25_O_10_+e]-(-1.81)  301.1079[C_17_H_17_O_5_+e]-(-0.79) | A |
| 30 | 4.67 | 461.1049 | M-H | C_22_H_22_O_11_ | -8.68 | 6-Cinnamoyl-1-galloylglucose | 461.1046[C_22_H_22_O_11_-H]-(-9.41)  271.0456[C_11_H_12_O_8_-H]-(-1.22)  169.0143[C_7_H_5_O_5_+e]-(0.47) | E |
| 31 | 4.99 | 435.1292 | M+FA-H | C_20_H_22_O_8_ | -1.33 | (Z)-Resveratrol 4'-glucoside | 435.1323[C_20_H_22_O_8_+FA-H]-(5.95)  315.0851[C_16_H_14_O_4_+FA-H]-(-7.36)  167.0347[C_7_H_6_O_2_+FA-H]-(-1.64) | E |
| 32 | 5.33 | 831.2184 | M+FA-H | C_34_H_42_O_21_ | -2.08 | Isorhamnetin 3-O-[b-D-glucopyranosyl-(1->2)-[a-L-rhamnopyranosyl-(1->6)]-b-D-glucopyranoside] | 831.2197[C_34_H_42_O_21_+FA-H]-(-0.53)  787.2256[C_34_H_42_O_21_+e+H]-(-5.92)  624.1750[C_28_H_31_O_16_+e+H]-(8.81) | E |
| 33 | 5.38 | 613.1147 | M-H | C_29_H_26_O_15_ | -8.53 | 6-Cinnamoyl-1,2-digalloylglucose | 465.0663[C_20_H_17_O_13_+e]-(-2.44)  241.0354[C_10_H_10_O_7_-H]-(2.63)  169.0142[C_7_H_5_O_5_+e]-(11.12) | E |
| 34 | 5.43 | 267.0671 | M-H | C_16_H_12_O_4_ | 3.16 | Isoformononetin | 267.0621[C_16_H_12_O_4_-H]-(-15.69)  252.0399[C_15_H_9_O_4_-H]-(-11.71) | A |
| 35 | 5.60 | 475.1262 | M+FA-H | C_22_H_22_O_9_ | 3.86 | Ononin | 475.1270[C_22_H_22_O_9_+FA-H]-(5.01)  329.0836[C_13_H_15_O_7_+FA-H+H]-(-12.62)  267.0659[C_16_H_11_O_4_+e]-(-1.47)  252.0661[C_12_H_12_O_6_+e]-(8.42) | A |
| 36 | 5.62 | 461.1098 | M-H | C_22_H_22_O_11_ | 1.90 | 2-Cinnamoyl-1-galloyl-beta-D-glucopyranose | 461.1055[C_22_H_22_O_11_-H]-(-7.49)  313.0540[C_13_H_13_O_9_+e]-(-7.99)  271.0440[C_11_H_12_O_8_-H]-(-7.07)  169.0130[C_7_H_5_O_5_+e]-(-7.29) | E |
| 37 | 5.84 | 903.2313 | M+FA-H | C_39_H_42_N_2_O_0_ | -0.05 | Betanidin 5-[E-feruloyl-(->5)-apiosyl-(1->2)-glucoside] | 903.2233[C_39_H_42_N_2_O_20_+FA-H]-(-8.83)  859.2123[C_37_H_38_N_2_O_19_+FA-H]- (8.42)  697.1974[C_34_H_35_NO_15_+e]-(-5.45) | E |
| 38 | 5.88 | 415.0995 | M-H | C_21_H_0_O | -9.61 | Pulmatin | 415.1059[C_21_H_20_O_9_-H]-(6.03)  397.0888[C_21_H_18_O_8_-H]-(-10.45)  253.0510[C_15_H_9_O_4_+e]-(1.55)  161.0456[C_6_H_9_O_5_+e]-(0.37) | E |
| 39 | 6.14 | 283.0616 | M-H | C_16_H_12_O_5_ | 1.55 | Biochanin A | 283.0605[C_16_H_12_O_5_-H]-(-2.39)  268.0377[C_15_H_9_O_5_-H]-(-0.13)  251.0342[C_15_H_7_O_4_+e]-(-3.00) | A, E |
| 40 | 6.14 | 613.1204 | M+FA-H | C_28_H_24_O_13_ | 0.93 | Catechin pentaacetate | 613.1196[C_28_H_24_O_13_+FA-H]-(-0.55)  401.0879[C_19_H_16_O_7_+FA-H]-(0.29)  313.0547[C_13_H_13_O_9_+e]-(-6.04)  271.0447[C_10_H_10_O_6_+FA-H]-(-4.41) | E |
| 41 | 6.22 | 517.0940 | M-H | C_24_H_22_O_13_ | -9.22 | 6''-Malonylgenistin | 517.1044[C_24_H_22_O_13_-H]-(10.92)  473.1032[C_23_H_21_O_11_+e]-(-12.19)  311.0582[C_17_H_12_O_6_-H]-(6.57)  269.0436[C_15_H_9_O_5_+e]-(-7.09) | A, E |
| 42 | 6.27 | 285.0389 | M-H | C_15_H_10_O_6_ | -5.57 | Kaempferol | 285.0435[C_15_H_10_O_6_-H]-(10.64)  268.0371[C_15_H_9_O_5_-H]-(-2.29)  211.0383[C_13_H_7_O_3_+e]-(-8.50) | A, E |
| 43 | 6.34 | 267.0673 | M-H | C_16_H_12_O_4_ | 3.79 | Formononetin | 267.0665[C_16_H_12_O_4_-H]-(0.54)  252.0428[C_15_H_9_O_4_-H]-(0.03) | A |
| 44 | 6.68 | 253.0494 | M-H | C_15_H_10_O_4_ | -4.93 | Phomarin | 225.0534[C_14_H_10_O_3_-H]-(-10.37)  238.0311[C_14_H_7_O_4_-H]-(-16.76)  236.0462[C_15_H_9_O_3_-H]-(-7.48)  105.0339[C_7_H_6_O-H]-(-7.20) | E |
| 45 | 6.90 | 473.1095 | M-H | C_23_H_22_O_11_ | 1.10 | Kaempferol 3-(2''-acetylrhamnoside) | 473.1057[C_23_H_22_O_11_-H]-(-6.72)  455.0926[C_23_H_20_O_10_-H]-(-12.60)  431.1017[C_21_H_19_O_10_+e]-(7.74)  269.0418[C_15_H_9_O_5_+e]-(-13.79) | E |
| 46 | 6.91 | 253.0511 | M-H | C_15_H_10_O_4_ | 1.86 | 1,8-dihydroxy-3-methylanthracene-9,10-dione | 253.0496[C_15_H_10_O_4_-H]-(-3.96)  236.0409[C_15_H_9_O_3_-H]-(-4.51)  225.0526[C_14_H_10_O_3_-H]-(-13.76)  207.0415[C_14_H_8_O_2_-H]-(-7.56) | E |
| 47 | 7.39 | 169.0154 | M-H | C_7_H_6_O_5_ | 6.85 | Gallic acid | 169.0136[C_7_H_6_O_5_-H]-(-3.44)  125.0231[C_6_H_5_O_3_+e]-(-10.59)  107.0159[C_6_H_3_O_2_+e]-(9.17) | E |
| 48 | 7.58 | 491.1202 | M+FA-H | C_22_H_22_O_10_ | 1.47 | Rheochrysin | 491.1152[C_22_H_22_O_10_+FA-H]-(-8.69)  283.0573[C_16_H_11_O_5_+e]-(-13.66)  268.0366[C_15_H_8_O_5_+e]-(-4.32) | E |
| 49 | 7.90 | 829.4584 | M+FA-H | C_41_H_68_O_14_ | -0.89 | Astragaloside IV | 829.4498[C_41_H_68_O_14_+FA-H]-(-11.18)  783.4264[C_39_H_62_O_13_+FA-H]-(11.73)  621.4026[C_35_H_57_O_9_+e]-(2.80)  161.0490[C_6_H_9_O_5_+e]-(21.60) | A |
| 50 | 7.91 | 491.1194 | M-H | C_23_H_24_O_12_ | -0.24 | Licoagroside A | 491.1159[C_23_H_24_O_12_-H]-(-7.43)  446.1155[C_22_H_22_O_10_+e]-(-14.28)  283.0854[C_13_H_15_O_7_+e]-(10.74) | A |
| 51 | 8.07 | 957.5086 | M-H | C_48_H_78_O_19_ | 2.20 | Soyasaponin A3 | 911.4944[C_47_H_76_O_17_-H]-(-7.22)  587.3937[C_35_H_54_O_7_+e+H]-(-2.85)  323.1017[C_12_H_21_O_10_-H-H]-(10.59) | A |
| 52 | 8.15 | 267.0662 | M-H | C_16_H_12_O_4_ | -0.14 | 1,3-Dimethoxyanthraquinone | 267.0641[C_16_H_12_O_4_-H]-(-8.37)  252.0425[C_15_H_9_O_4_-H]-(-1.36)  132.0211[C_8_H_4_O_2_+e]-(-4.02) | A |
| 53 | 8.42 | 523.1135 | M+FA-H | C_22_H_22_O_12_ | 8.72 | 6-Methoxyluteolin 3'-glucoside | 523.1086[C_22_H_22_O_12_+FA-H]-(-1.26)  479.1134[C_22_H_22_O_12_+e+H]-(-12.89)  463.0935[C_21_H_19_O_12_+e]-(11.42)  283.0795[C_13_H_15_O_7_+e]-(-9.96) | E |
| 54 | 8.70 | 311.0567 | M-H | C_17_H_12_O_6_ | 1.78 | Aloe emodin w-acetate | 311.0563[C_17_H_12_O_6_-H]-(0.58)  293.0427[C_17_H_10_O_5_-H]-(-9.55)  268.0374[C_15_H_9_O_5_-H]-(-1.04)  240.0430[C_14_H_7_O_4_+e+H]-(0.50) | E |
| 55 | 8.77 | 829.4607 | M+FA-H | C_41_H_68_O_14_ | 2.01 | Astragaloside III | 829.4575[C_41_H_68_O_14_+FA-H]-(-1.91)  783.4117[C_39_H_62_O_13_+FA-H]-(-6.97)  621.3975[C_35_H_57_O_9_+e]-(-5.35)  179.0555[C_6_H_11_O_6_+e]-(-3.73) | A |
| 56 | 8.99 | 861.1898 | M-H | C_42_H_38_O_20_ | 1.65 | Sennoside B | 861.1796[C_42_H_38_O_20_-H]-(-10.10)  700.1451[C_36_H_29_O_15_-H]-(2.51)  431.0941[C_21_H_19_O_10_+e]-(-9.89) | E |
| 57 | 9.08 | 523.1021 | M-H | C_30_H_20_O_9_ | -2.58 | Rheidin A | 463.0803[C_28_H_16_O_7_-H] -(-4.39)  268.0359[C_15_H_9_O_5_-H]-(-6.96)  253.0465[C_15_H_11_O_4_-H-H]-(-16.26) | E |
| 58 | 9.73 | 987.5213 | M+FA-H | C_48_H_78_O_18_ | 3.08 | Soyasaponin I | 941.5171[C_48_H_78_O_18_-H]-(5.94)  880.5207[C_47_H_75_O_15_+e]-(1.98)  733.4528[C_41_H_64_O_11_+e+H]-(-0.67)  616.3970[C_36_H_57_O_8_+e-H]-(-1.75) | A |
| 59 | 9.83 | 613.1204 | M-H | C_29_H_2_O_15_ | 0.80 | 2-Cinnamoyl-1,6-digalloyl-beta-D-glucopyranose | 613.1238[C_29_H_26_O_15_-H]-(6.39)  465.0667[C_20_H_17_O_13_+e]-(-1.59)  271.0456[C_11_H_12_O_8_-H]-(-1.11)  169.0148[C_7_H_5_O_5_+e]-(3.36) | E |
| 60 | 10.16 | 269.0432 | M-H | C_15_H_10_O_5_ | -8.86 | Aloeemodin | 269.0452[C_15_H_10_O_5_-H]-(-1.10)  239.0348[C_14_H_7_O_4_+e]-(-0.73)  223.0390[C_14_H_8_O_3_-H]-(-4.51) | E |
| 61 | 10.20 | 431.0993 | M-H | C_21_H_20_O_10_ | 2.06 | Apiolin-7-o-glocoside | 431.1009[C_21_H_20_O_10_-H]-(6.03)  311.0538[C_17_H_12_O_6_-H]-(-7.46)  293.0480[C_17_H_10_O_5_-H]-(8.46)  253.0501[C_15_H_9_O_4_+e]-(-2.07) | E |
| 62 | 10.21 | 811.4517 | M+FA-H | C_41_H_66_O_13_ | 4.13 | Soyasaponin IV | 765.4431[C_40_H_64_O_11_+FA-H]-(-2.77)  603.3914[C_35_H_56_O_8_+e-H]-(1.84)  161.0468[C_6_H_10_O_5_+e-H]-(7.95) | A |
| 63 | 10.96 | 685.2327 | M-H | C_31_H_42_O_17_ | -3.18 | nuezhenide | 685.2313[C_31_H_42_O_17_ -H]-(-5.25)  523.1808[C_25_H_31_O_12_+e]-(-2.56)  421.1482[C_20_H_24_O_7_+FA-H]-(-5.18)  223.0614[C_11_H_12_O_5_-H]-(0.95) | C |
| 64 | 11.36 | 315.2531 | M+FA-H | C_17_H_34_O_2_ | -3.67 | Methyl palmitate | 315.2509[C_17_H_34_O_2_+FA-H]-(-10.11)  239.2416[C_16_H_31_O+e]-(15.00)  157.1235[C_9_H_17_O_2_+e]-(0.36) | B, D, E, F |
| 65 | 11.46 | 277.1439 | M+FA-H | C_15_H_20_O_2_ | -2.66 | (7b,10a)-3-Hydroxy-1,3,5-cadinatrien-9-one | 277.1407[C_16_H_22_O_4_+FA-H]-(-13.66)  233.1511[C_15_H_20_O_2_+e+H]-(-15.48)  134.0370[C_7_H_4_+FA-H+H]-(-2.47) | B, D, E, F |
| 66 | 11.50 | 269.0461 | M-H | C_15_H_10_O_5_ | 1.92 | Emodin | 269.0449[C_15_H_10_O_5_-H]-(-2.46)  241.0502[C_14_H_10_O_4_-H]-(-1.69)  225.0552[C_14_H_8_O_3_+e+H]-(-2.55)  182.0347[C_12_H_5_O_2_+e+H]-(-4.64) | E |
| 67 | 11.75 | 311.2222 | M-H | C_18_H_32_O_4_ | -1.92 | 13-L-Hydroperoxylinoleic acid | 311.2206[C_18_H_32_O_4_-H]-(-7.30)  293.2085[C_18_H_30_O_3_-H]-(-12.71)  223.1682[C_14_H_25_O_2_-H-H]-(-9.46)  125.0970[C_8_H_13_O+e]-(-0.94) | B, F |
| 68 | 11.80 | 539.1754 | M-H | C_25_H_32_O_13_ | -3.05 | oleuropein | 539.1748[C_25_H_32_O_13_-H]-(-4.12)  377.1213[C_19_H_21_O_8_+e]-(-7.53)  307.0822[C_15_H_16_O_7_-H]-(-0.55)  275.0927[C_15_H_14_O_5_+e+H]-(0.96) | C |
| 69 | 12.01 | 507.1040 | M-H | C_30_H_20_O_8_ | -8.98 | Rheidin B | 463.1135[C_29_H_19_O_6_+e]-(-11.34)  445.1097[C_29_H_17_O_5_+e]-(3.582)  269.0437[C_15_H_9_O_5_+e]-(-6.75)  238.0633[C_15_H_11_O_3_-H]-(-1.03) | E |
| 70 | 12.08 | 253.0496 | M-H | C_15_H_10_O_4_ | -4.22 | Chrysophanol | 252.0425[C_15_H_10_O_4_-H-H]-(-1.30)  225.0574[C_14_H_10_O_3_-H]-(7.67)  207.0440[C_14_H_8_O_2_-H]-(-5.62)  182.0369[C_12_H_5_O_2_+e+H]-(-2.66) | E |
| 71 | 15.79 | 455.3532 | M-H | C_30_H_48_O_3_ | 0.38 | 3-Epikatonic acid | 455.3483[C_30_H_48_O_3_-H]-(-10.38)  409.3440[C_29_H_47_O-H-H]-(-8.79)  337.2879[C_25_H_38_-H]-(-6.49)  269.2296[C_20_H_30_-H]-(7.88) | A |
| 72 | 17.25 | 776.5470 | M+FA-H | C_40_H_77_NO_10_ | -8.14 | Araliacerebroside | 731.5124[C_38_H_72_NO_9_+FA-H]-(-8.90)  730.5558[C_40_H_77_NO_10_+e-H]-(11.41)  568.5005[C_34_H_66_NO_5_+e]-(10.27)  271.2268[C_15_H_31_O+FA-H-H]-(-3.93) | E |

A: Astragaliradix; B: Pseudostellariae radix; C: Ligustri lucidi fructus; D: Lycii fructus; E: Rhei radix et rhizome; F: Hirudo

**Table S5. Blood transitional components of KLX detected by UPLC-Q/TOF-MS in positive- and negative-ion modes.**

| **NO.** | **Rt / min** | **m/z** | **Adducts** | **Fomula** | **Error / ppm** | **Compouds Name** | **Mass Fragments** | **Source** |
| --- | --- | --- | --- | --- | --- | --- | --- | --- |
| 1 | 0.45 | 248.1125 | M+H | C_10_H_17_NO_6_ | -1.59 | Linamarin | 248.1127[C_10_H_17_NO_6_+H]+(-0.64)  128.0683[C_6_H_9_NO_2_+H]+(-17.43)  98.0606[C_5_H_7_NO+H]+(5.65)  70.0662[C_4_H_6_N+H+H]+(15.82) | A, B, E |
| 2 | 0.48 | 549.1642 | M+FA-H | C_18_H_32_O_16_ | -6.48 | Raffinose | 503.1584[C_18_H_32_O_16_+e-H]-(-6.74)  383.1166[C_14_H_23_O_12_+e]-(-7.64)  323.0958[C_12_H_19_O_10_+e]-(-8.00)  143.0344[C_6_H_8_O_4_-H]-(-4.03) | A, B |
| 3 | 0.49 | 711.2162 | M+FA-H | C_24_H_42_O_21_ | -2.63 | Stachyose | 665.2079[C_24_H_42_O_21_+e-H]-(-10.02)  485.1444[C_18_H_29_O_15_+e]-(-13.95)  383.1196[C_14_H_23_O_12_+e]-(0.21)  179.0550[C_6_H_11_O_6_+e]-(-6.20) | A, B, E |
| 4 | 0.50 | 527.1592 | M+Na | C_18_H_32_O_16_ | 1.82 | alpha-D-Glucopyranosyl-(1->6)-alpha-D-glucopyranosyl-(1->2)-D-glucose | 527.1580[C_18_H_32_O_16_+Na]+(-0.38)  437.1207[C_15_H_26_O_13_+Na]+(-13.39)  365.1118[C_14_H_21_O_11_-e]+(10.78)  203.0570[C_8_H_11_O_6_-e]+(9.71) | A, B, D |
| 5 | 0.88 | 341.1095 | M-H | C_12_H_22_O_11_ | 1.60 | Sophorose | 341.1072[C_12_H_22_O_11_+e-H]-(-5.14)  252.0858[C_9_H_16_O_8_+e]-(2.98)  131.0350[C_5_H_8_O_4_-H]-(-0.26) | A, B, E |
| 6 | 1.13 | 331.0672 | M-H | C_13_H_16_O_10_ | 0.28 | 3-Glucogallic acid | 331.0658[C_13_H_16_O_10_-H]-(-3.62)  271.0469[C_11_H_12_O_8_-H]-(3.74)  241.0359[C_10_H_10_O_7_-H]-(2.12)  151.0037[C_7_H_3_O_4_+e]-(0.59) | E |
| 7 | 1.85 | 367.1507 | M+H | C_17_H_22_N_2_O_7_ | 1.99 | Semilepidinoside B | 367.1504[C_17_H_22_N_2_O_7_+H]+(1.21)  350.1441[C_17_H_21_N_2_O_6_+H]+(-8.97)  276.1255[C_15_H_16_NO_4_+H+H]+(8.85)  188.0665[C_8_H_10_O_5_+H+H]+(-7.09) | A, B, D, E |
| 8 | 2.48 | 433.1128 | M+H | C_21_H_20_O_10_ | -0.30 | Emodin-6-O-beta-D-glucopyranoside | 397.0952[C_21_H_17_O_8_-e]+(8.57)  271.0581[C_15_H_9_O_5_+H+H]+(-7.38)  253.0497[C_15_H_9_O_4_-e]+(0.40) | E |
| 9 | 2.49 | 479.1154 | M-H | C_22_H_24_O_12_ | -8.45 | 3'-O-Methyl-(-)-epicatechin 7-O-glucuronide | 479.1195[C_22_H_24_O_12_-H]-(-5.19)  355.0670[C_15_H_17_O_10_-H-H]-(-13.94)  299.0408[C_12_H_12_O_9_-H]-(4.83)  255.0663[C_15_H_13_O_4_-H-H]-(-1.05) | Metabolite of E |
| 10 | 2.62 | 595.1667 | M+H | C_27_H_30_O_15_ | 1.64 | Apigenin 7-[galactosyl-(1->4)-mannoside] | 595.1584[C_27_H_30_O_15_+H]+(-12.42)  523.1378[C_24_H_25_O_13_+H+H]+(-13.04)  475.1057[C_19_H_24_O_14_-e-H]+(-5.47)  295.0566[C_17_H_10_O_5_+H]+(-11.75) | B, C |
| 11 | 2.66 | 409.1119 | M+Na | C_17_H_22_O_10_ | 3.48 | 1-O-Sinapoylglucose | 409.1074[C_17_H_22_O_10_+Na]+(-7.57)  247.0601[C_11_H_11_O_5_+Na+H]+(9.71)  185.0411[C_6_H_11_O_5_+Na-H]+(-5.10) | E |
| 12 | 2.74 | 461.1055 | M+FA-H | C_21_H_20_O_9_ | -8.34 | Chrysophanol-8-O-β-D-glucopyranoside | 337.0752[C_19_H_13_O_6_+e]-(10.32)  299.0551[C_15_H_10_O_4_+FA-H]-(-3.30)  255.0649[C_14_H_9_O_2_+FA-H+H]-(-5.40) | E |
| 13 | 2.88 | 577.1344 | M-H | C_30_H_26_O_12_ | -1.24 | Procyanidin B1 | 577.1423[C_30_H_26_O_12_-H]-(12.39)  425.0912[C_22_H_18_O_9_-H]-(8.05)  289.0699[C_15_H_13_O_6_+e]-(-6.34) | E |
| 14 | 2.90 | 565.1567 | M+H | C_26_H_28_O_14_ | 2.62 | Kaempferol 3-arabinofuranoside 7-rhamnofuranoside | 587.1369[C_26_H_28_O_14_+Na]+(-0.37)  565.1599[C_26_H_28_O_14_+H]+(8.38)  481.1165[C_25_H_22_O_10_-e-H]+(7.41)  349.0939[C_17_H_15_O_8_+H+H]+(6.00) | B |
| 15 | 3.32 | 271.0599 | M+H | C_15_H_10_O_5_ | -0.62 | 3,4',7-Trihydroxyflavone | 271.0577[C_15_H_10_O_5_+H]+(-8.85)  225.0538[C_14_H_8_O_3_+H]+(-3.44)  197.0584[C_13_H_7_O_2_+H+H]+(-6.42)  77.0376[C_6_H_3_+H+H]+(-12.71) | E |
| 16 | 3.46 | 579.1680 | M+Na | C_25_H_32_O_14_ | -0.83 | 10-Hydroxyoleuropein | 579.1690[C_25_H_32_O_14_+Na]+(1.01)  433.1180[C_21_H_22_O_10_-e-H]+(11.54)  399.1058[C_19_H_21_O_8_+Na-H]+(1.89)  301.0702[C_14_H_15_O_6_+Na-H]+(6.44) | C |
| 17 | 3.62 | 419.1344 | M+Na | C_19_H_24_O_9_ | 8.06 | Aloesol 7-glucoside | 419.1340[C_19_H_24_O_9_+Na]+(6.64)  401.1256[C_19_H_22_O_8_+Na]+(12.11)  217.0481[C_10_H_11_O_4_+Na-H]+(4.63) | E |
| 18 | 3.66 | 369.1175 | M+Na | C_15_H_22_O_9_ | 5.59 | 1-(3-Hydroxy-4-Methoxyphenyl)-1,2-ethanediol 3'-O-b-D-glucoside | 369.1174[C_15_H_22_O_9_+Na]+(4.82)  295.0823[C_12_H_16_O_7_+Na]+(11.91)  207.0609[C_9_H_11_O_4_+Na+H]+(-9.14)  147.0414[C_7_H_8_O_2_+Na]+(-1.52) | C |
| 19 | 3.87 | 233.0825 | M-H | C_13_H_14_O_4_ | 2.35 | Aloesol | 233.0852[C_13_H_14_O_4_-H]-(3.95)  215.0748[C_13_H_12_O_3_-H]-(6.23)  189.0588[C_11_H_9_O_3_+e]-(16.10) | E |
| 20 | 4.09 | 461.0709 | M-H | C_21_H_18_O_12_ | -3.53 | Kaempferol 3-glucuronide | 285.0404[C_15_H_9_O_6_+e]-(-1.22)  257.0455[C_14_H_9_O_5_+e]-(13.11)  211.0400[C_13_H_7_O_3_+e]-(7.47) | Metabolite of A and E |
| 21 | 4.12 | 591.1350 | M-H | C_27_H_28_O_15_ | -0.98 | Apigenin 7-[rhamnosyl-(1->2)-galacturonide] | 429.0829[C_21_H_17_O_10_+e]-(0.46)  253.0466[C_15_H_9_O_4_+e]-(-16.00)  163.0382[C_9_H_6_O_3_+e+H]-(-11.29)  85.0285[C_4_H_6_O_2_-H]-(-11.73) | Metabolite of E |
| 22 | 4.36 | 447.0902 | M-H | C_21_H_20_O_11_ | -6.81 | Luteolin 7-glucoside | 447.0899[C_21_H_20_O_11_-H]-(-7.71)  327.0471[C_17_H_12_O_7_-H]-(-11.84)  285.0388[C_15_H_9_O_6_+e]-(-5.83)  256.0379[C_14_H_9_O_5_-H]-(0.70) | E |
| 23 | 4.41 | 191.0697 | M+H | C_11_H_10_O_3_ | -3.24 | 7-Hydroxy-2,5-dimethyl-4H-1-benzopyran-4-one | 189.0562[C_11_H_10_O_3_-e-H]+(8.26)  176.0475[C_10_H_7_O_3_+H]+(3.85)  161.0597[C_10_H_9_O_2_-e]+(-0.08) | E |
| 24 | 4.50 | 431.0955 | M-H | C_21_H_20_O_10_ | -6.69 | Glucoemodin | 293.0447[C_17_H_10_O_5_-H]-(-2.78)  269.0433[C_15_H_9_O_5_+e]-(-8.45)  239.0379[C_14_H_9_O_4_-H-H]-(12.17) | E |
| 25 | 4.91 | 433.1119 | M+H | C_21_H_20_O_10_ | -2.40 | Anthraglycoside B | 415.1009[C_21_H_19_O_9_-e]+(-3.59)  397.0966[C_21_H_17_O_8_-e]+(12.18)  271.0595[C_15_H_9_O_5_+H+H]+(-2.20)  253.0487[C_15_H_9_O_4_-e]+(-3.52) | A, E |
| 26 | 5.27 | 445.1129 | M+Na | C_20_H_22_O_10_ | 5.71 | Rumexoside | 445.1185[C_20_H_22_O_10_+Na]+(-4.49)  427.0952[C_20_H_20_O_9_+Na]+(-10.95)  153.0166[C_5_H_6_O_4_+Na]+(5.20) | E |
| 27 | 5.43 | 267.0671 | M-H | C_16_H_12_O_4_ | 3.16 | Isoformononetin | 267.0621[C_16_H_12_O_4_-H]-(-15.69)  252.0399[C_15_H_9_O_4_-H]-(-11.71) | A |
| 28 | 5.60 | 475.1262 | M+FA-H | C_22_H_22_O_9_ | 3.86 | Ononin | 475.1270[C_22_H_22_O_9_+FA-H]-(5.01)  329.0836[C_13_H_15_O_7_+FA-H+H]-(-12.62)  267.0659[C_16_H_11_O_4_+e]-(-1.47)  252.0661[C_12_H_12_O_6_+e]-(8.42) | A |
| 29 | 5.62 | 461.1098 | M-H | C_22_H_22_O_11_ | 1.90 | 2-Cinnamoyl-1-galloyl-beta-D-glucopyranose | 461.1055[C_22_H_22_O_11_-H]-(-7.49)  313.0540[C_13_H_13_O_9_+e]-(-7.99)  271.0440[C_11_H_12_O_8_-H]-(-7.07)  169.0130[C_7_H_5_O_5_+e]-(-7.29) | E |
| 30 | 5.62 | 429.0800 | M-H | C_21_H_18_O_10_ | -6.27 | Daidzein 7-O-glucuronide | 429.0847[C_21_H_18_O_10_-H]-(4.57)  253.0493[C_15_H_9_O_4_+e]-(-5.32)  175.0268[C_6_H_7_O_6_+e]-(11.38) | Metabolite of A |
| 31 | 5.68 | 441.1167 | M+Na | C_21_H_22_O_9_ | 2.60 | Aloin | 423.1046[C_21_H_20_O_8_+Na]+(-1.10)  321.0737[C_17_H_14_O_5_+Na]+(1.28)  185.0419[C_6_H_11_O_5_+Na-H]+(-0.40) | E |
| 32 | 5.69 | 247.0963 | M+H | C_14_H_14_O_4_ | -0.72 | Torachrysone | 247.0938[C_14_H_14_O_4_+H]+(-10.73)  214.0623[C_13_H_9_O_3_+H]+(-0.75)  186.0712[C_12_H_9_O_2_+H]+(19.38)  147.0469[C_9_H_6_O_2_+H]+(19.24) | E |
| 33 | 6.14 | 283.0616 | M-H | C_16_H_12_O_5_ | 1.55 | Biochanin A | 283.0605[C_16_H_12_O_5_-H]-(-2.39)  268.0377[C_15_H_9_O_5_-H]-(-0.13)  251.0342[C_15_H_7_O_4_+e]-(-3.00) | A, E |
| 34 | 7.44 | 167.0693 | M+H | C_9_H_10_O_3_ | -6.12 | 3-Hydroxy-1-(4-hydroxyphenyl)-1-propanone | 167.0712[C_9_H_10_O_3_+H]+(5.24)  134.0362[C_8_H_7_O_2_-e-H]+(-0.20)  120.0196[C_7_H_5_O_2_-e-H]+(-8.05) | A |
| 35 | 7.90 | 829.4584 | M+FA-H | C_41_H_68_O_14_ | -0.89 | Astragaloside IV | 829.4498[C_41_H_68_O_14_+FA-H]-(-11.18)  783.4264[C_39_H_62_O_13_+FA-H]-(11.73)  621.4026[C_35_H_57_O_9_+e]-(2.80)  161.0490[C_6_H_9_O_5_+e]-(21.60) | A |
| 36 | 8.07 | 957.5086 | M-H | C_48_H_78_O_19_ | 2.20 | Soyasaponin A3 | 911.4944[C_47_H_76_O_17_-H]-(-7.22)  587.3937[C_35_H_54_O_7_+e+H]-(-2.85)  323.1017[C_12_H_21_O_10_-H-H]-(10.59) | A |
| 37 | 8.16 | 285.0394 | M+H | C_15_H_8_O_6_ | 0.23 | Rhein | 285.0388[C_15_H_8_O_6_+H]+(-1.96)  270.0542[C_15_H_8_O_5_+H+H]+(7.01)  241.0539[C_14_H_7_O_4_+H+H]+(17.79) | E |
| 38 | 8.86 | 279.2319 | M+Na | C_16_H_32_O_2_ | 9.52 | Isopalmitic acid | 279.2336[C_16_H_32_O_2_+Na]+(15.12)  261.2194[C_16_H_30_O+Na]+(1.68)  209.1512[C_11_H_21_O_2_+Na+H]+(3.62) | A, C |
| 39 | 9.72 | 455.3518 | M+H | C_30_H_46_O_3_ | -0.36 | 9(11)-Dehydroglycyrrhetic acid | 455.3490[C_30_H_46_O_3_+H]+(-6.54)  419.3343[C_30_H_43_O-e]+(8.20) | A, C |
| 40 | 9.83 | 613.1204 | M-H | C_29_H_26_O_15_ | 0.80 | 2-Cinnamoyl-1,6-digalloyl-beta-D-glucopyranose | 613.1199[C_29_H_26_O_15_-H]-(6.39)  465.0674[C_20_H_17_O_13_+e]-(-1.59)  271.0459[C_11_H_12_O_8_-H]-(-1.11)  169.0142[C_7_H_5_O_5_+e]-(3.36) | E |
| 41 | 10.16 | 269.0432 | M-H | C_15_H_10_O_5_ | -8.86 | Aloeemodin | 269.0452[C_15_H_10_O_5_-H]-(-1.10)  239.0348[C_14_H_7_O_4_+e]-(-0.73)  223.0390[C_14_H_8_O_3_-H]-(-4.51) | E |
| 42 | 10.19 | 457.3315 | M+FA-H | C_28_H_44_O_2_ | -2.06 | 25-Hydroxyvitamin D2 | 457.3323[C_28_H_44_O_2_+FA-H]-(5.55)  413.3061[C_26_H_40_O+FA-H]-(7.36)  411.3268[C_28_H_44_O_2_+e-H]-(4.22) | Metabolite |
| 43 | 10.21 | 811.4517 | M+FA-H | C_41_H_66_O_13_ | 4.13 | Soyasaponin IV | 765.4431[C_40_H_64_O_11_+FA-H]-(-2.77)  603.3914[C_35_H_56_O_8_+e-H]-(1.84)  161.0468[C_6_H_10_O_5_+e-H]-(7.95) | A |
| 44 | 10.67 | 455.3525 | M+H | C_30_H_46_O_3_ | 1.14 | Glypallidifloric acid | 455.3551[C_30_H_46_O_3_+H]+(6.93)  191.1807[C_14_H_22_+H]+(6.45)  123.1165[C_9_H_14_+H]+(-3.05)  109.1015[C_8_H_11_+H+H]+(2.91) | A, C |
| 45 | 11.50 | 269.0461 | M-H | C_15_H_10_O_5_ | 1.92 | Emodin | 269.0449[C_15_H_10_O_5_-H]-(-2.46)  241.0502[C_14_H_10_O_4_-H]-(-1.69)  225.0552[C_14_H_8_O_3_+e+H]-(-2.55)  182.0347[C_12_H_5_O_2_+e+H]-(-4.64) | E |
| 46 | 12.08 | 253.0496 | M-H | C_15_H_10_O_4_ | -4.22 | Chrysophanol | 252.0425[C_15_H_10_O_4_-H-H]-(-1.30)  225.0574[C_14_H_10_O_3_-H]-(7.67)  207.0440[C_14_H_8_O_2_-H]-(-5.62)  182.0369[C_12_H_5_O_2_+e+H]-(-2.66) | E |

A: Astragaliradix; B: Pseudostellariae radix; C: Ligustri lucidi fructus; D: Lycii fructus; E: Rhei radix et rhizome; F: Hirudo
